# PseudoFuN: Deriving functional potentials of pseudogenes from integrative relationships with genes and microRNAs across 32 cancers

**DOI:** 10.1093/gigascience/giz046

**Published:** 2019-04-26

**Authors:** Travis S Johnson, Sihong Li, Eric Franz, Zhi Huang, Shuyu Dan Li, Moray J Campbell, Kun Huang, Yan Zhang

**Affiliations:** 1 Department of Biomedical Informatics, College of Medicine, The Ohio State University, 1800 Cannon Drive, Columbus, OH 43210, USA; 2 Ohio Supercomputer Center, 1224 Kinnear Road, Columbus, OH 43212, USA; 3 School of Electrical and Computer Engineering, Purdue University, 465 Northwestern Avenue, West Lafayette, IN 47907, USA; 4 Department of Medicine, Indiana University School of Medicine, 545 Barnhill Drive, Indianapolis, IN 46202, USA; 5 Department of Genetics and Genomic Sciences, Icahn School of Medicine at Mount Sinai, One Gustave L. Levy Place, New York, NY 10029, USA; 6 Division of Pharmaceutics and Pharmaceutical Chemistry, College of Pharmacy, The Ohio State University, 500 West 12 ^th^ Avenue, Columbus, OH 43210, USA; 7 Regenstrief Institute, Indiana University, 1101 West 10 ^th^ Street, Indianapolis, IN 46262, USA; 8 The Ohio State University Comprehensive Cancer Center (OSUCCC - James), 460 West 10 ^th^ Avenue, Columbus, OH 43210, USA

**Keywords:** pseudogenes, database, functional prediction, gene regulation, network analysis, high-performance computing, graphics processing unit, competing endogenous RNA

## Abstract

**Background:**

Long thought “relics” of evolution, not until recently have pseudogenes been of medical interest regarding regulation in cancer. Often, these regulatory roles are a direct by-product of their close sequence homology to protein-coding genes. Novel pseudogene-gene (PGG) functional associations can be identified through the integration of biomedical data, such as sequence homology, functional pathways, gene expression, pseudogene expression, and microRNA expression. However, not all of the information has been integrated, and almost all previous pseudogene studies relied on 1:1 pseudogene–parent gene relationships without leveraging other homologous genes/pseudogenes.

**Results:**

We produce PGG families that expand beyond the current 1:1 paradigm. First, we construct expansive PGG databases by (i) CUDAlign graphics processing unit (GPU) accelerated local alignment of all pseudogenes to gene families (totaling 1.6 billion individual local alignments and >40,000 GPU hours) and (ii) BLAST-based assignment of pseudogenes to gene families. Second, we create an open-source web application (PseudoFuN [Pseudogene Functional Networks]) to search for integrative functional relationships of sequence homology, microRNA expression, gene expression, pseudogene expression, and gene ontology. We produce four “flavors” of CUDAlign-based databases (>462,000,000 PGG pairwise alignments and 133,770 PGG families) that can be queried and downloaded using PseudoFuN. These databases are consistent with previous 1:1 PGG annotation and also are much more powerful including millions of *de novo* PGG associations. For example, we find multiple known (e.g., *miR-20a*-*PTEN*-*PTENP1*) and novel (e.g., *miR-375*-*SOX15*-*PPP4R1L*) microRNA-gene-pseudogene associations in prostate cancer. PseudoFuN provides a “one stop shop” for identifying and visualizing thousands of potential regulatory relationships related to pseudogenes in The Cancer Genome Atlas cancers.

**Conclusions:**

Thousands of new PGG associations can be explored in the context of microRNA-gene-pseudogene co-expression and differential expression with a simple-to-use online tool by bioinformaticians and oncologists alike.

## Background

Pseudogenes were previously considered unimportant relics of evolution that played an unclear role in biological processes [1]. However, more pseudogenes have been discovered to be involved in gene regulation [2–4]. These regulatory relationships between pseudogenes and genes have increasingly been explored, such as the transcriptional regulation of *PTEN* by pseudogene *PTENP1* in several cancer conditions [5]. *PTEN* acts as a tumor suppressor gene, which is underexpressed in gastric cancer. However, by overexpressing *PTENP1* in gastric cancer, both *PTEN* underexpression and cell proliferation are mitigated via the regulatory relationship between *PTEN* and *PTENP1* [6]. Relationships between these pseudogenes and their parent genes have been found to play critical roles indicating functional potentials of these pseudogenes [7, 8]. This point can most clearly be seen in the importance of the role that sequence homology between pseudogenes and coding genes plays in competing endogenous RNA (ceRNA) networks [9, 10]. In ceRNA networks the pseudogenes act as decoy targets for the microRNAs (miRNAs) targeting a protein-coding gene. In short, researchers have made huge strides in understanding pseudogenes from genomic variation to functional potentials [11, 12], and from “deciphering” the mechanism of ceRNA networks [13] to experimental validation [14].

With this progress, there has been renewed interest in pseudogenes, especially in relation to cancer [15]. This interest has even uncovered biomarkers in human cancer including but not limited to *SUMO1P3* upregulation as a diagnostic biomarker in gastric cancer and *OCT4-pg4* expression as a prognostic biomarker in hepatocellular carcinoma [16–18]. Pseudogene expression has been used to stratify tumor subtypes in seven distinct cancer types [19]. However, owing to the close sequence homology between pseudogenes and their parent genes, identifying the expression profile unique to a pseudogene or highly homologous gene can be challenging. Efforts have been made to address these technical challenges in estimating pseudogene expression using modified alignment and quantification techniques [20]. Perhaps more intriguing is that pseudogenes can be somatically acquired in cancer development effectively “representing a new class of mutations” [21, p.1] that can be either activating or inactivating mutations which function as an on/off switch [22]. Specific pseudogenes have been implicated in specific cancers. For example, *FTH1* regulates tumorigenesis in prostate cancer [23], *TP73-AS1* regulates proliferation in esophageal squamous cell carcinoma [24], and pseudogenes *NKAPP1*, *MSTO2P*, and *RPLP0P2* are associated with poor prognosis in lung adenocarcinoma [25].

For these reasons, having a complete understanding of these pseudogene-gene (PGG) relationships is important. While studying these relationships, a common conception is to only consider the pseudogenes in relation to their parent genes with highest homology [7–9, 26]. There have also been pioneering studies probing pseudogene functions through aligning them to parent proteins (corresponding to the parent genes) and then to parent protein domains [7, 27, 28].

The conventional idea of single parent genes may not be comprehensive enough to model the complex phylogenetic relationships involving multiple genes and pseudogenes in a homolog family. While pseudogenes diverged from their parent genes distantly in the past, only the daughter protein-coding genes other than the original parent gene may now exist. The result is that aligning to the true phylogenetic parent gene itself may not be possible. For this reason, we advocate the use of homologous gene families rather than single parent genes to compare against pseudogenes. By viewing the homologies as a weighted network instead of a single scalar value, we believe that new relationships can be uncovered.

We build the PGG family databases using two methods: (i) CUDAlign [29] based local alignment of all pseudogenes to gene families (totaling 1.6 billion individual local alignments and >40,000 graphics processing unit [GPU] hours). By aligning all pseudogenes to all gene families (CUDAlign), we can study underlying sequence homology and more easily set cutoffs to assign pseudogenes to gene families. (ii) Basic Local Alignment and Search Tool (BLAST) [30] based assignment of pseudogenes to gene families. This provides a fast heuristic search option. BLAST derivative methods have been commonly used to find parent genes in previous pseudogene studies [31, 32]. Using these two methods, we show that these pseudogenes are usually assigned to the gene family of their parent genes but are often not exclusively so. Besides, most pseudogenes can be categorized into processed pseudogenes and unprocessed pseudogenes depending on whether they came from retrotranscription of messenger RNAs [11, 33, 34]. We take these differences into account using both of our methods (CUDAlign and BLAST).

Furthermore, we make these data publicly downloadable from GitHub [35]. We also created an R Shiny web application called PseudoFuN (Pseudogene Functional Networks) [36] that supports querying the PGG databases, interactive visualization and functional analysis of the PGG networks, and visualization of PGG co-expression and miRNA binding (including binding prediction with Miranda [37], PicTar [38], and TargetScan [39]) using The Cancer Genome Atlas (TCGA) and GTEx (Genotype-Tissue Expression) Project–derived public data [20, 40, 41]. Besides, we provide another interactive web application hosted by the Ohio Supercomputer Center (OSC), which supports querying novel sequences against any of our PGG databases and visualization of the resulting PGG networks.

The PGG databases can be used to study pseudogene-gene-miRNA co-expression indicative of ceRNA networks across the entire TCGA. With these diverse tools provided by PseudoFuN, it is possible to generate hypotheses regarding (i) the regulatory roles of pseudogenes across tumor and normal tissue, (ii) PGG relationships through *de novo* reassignment of pseudogenes to gene families, and (iii) functional annotation of pseudogenes. We expect these databases and tools to have more use in cancer studies.

## Methods

### Construction of PGG database

**Figure 1:**
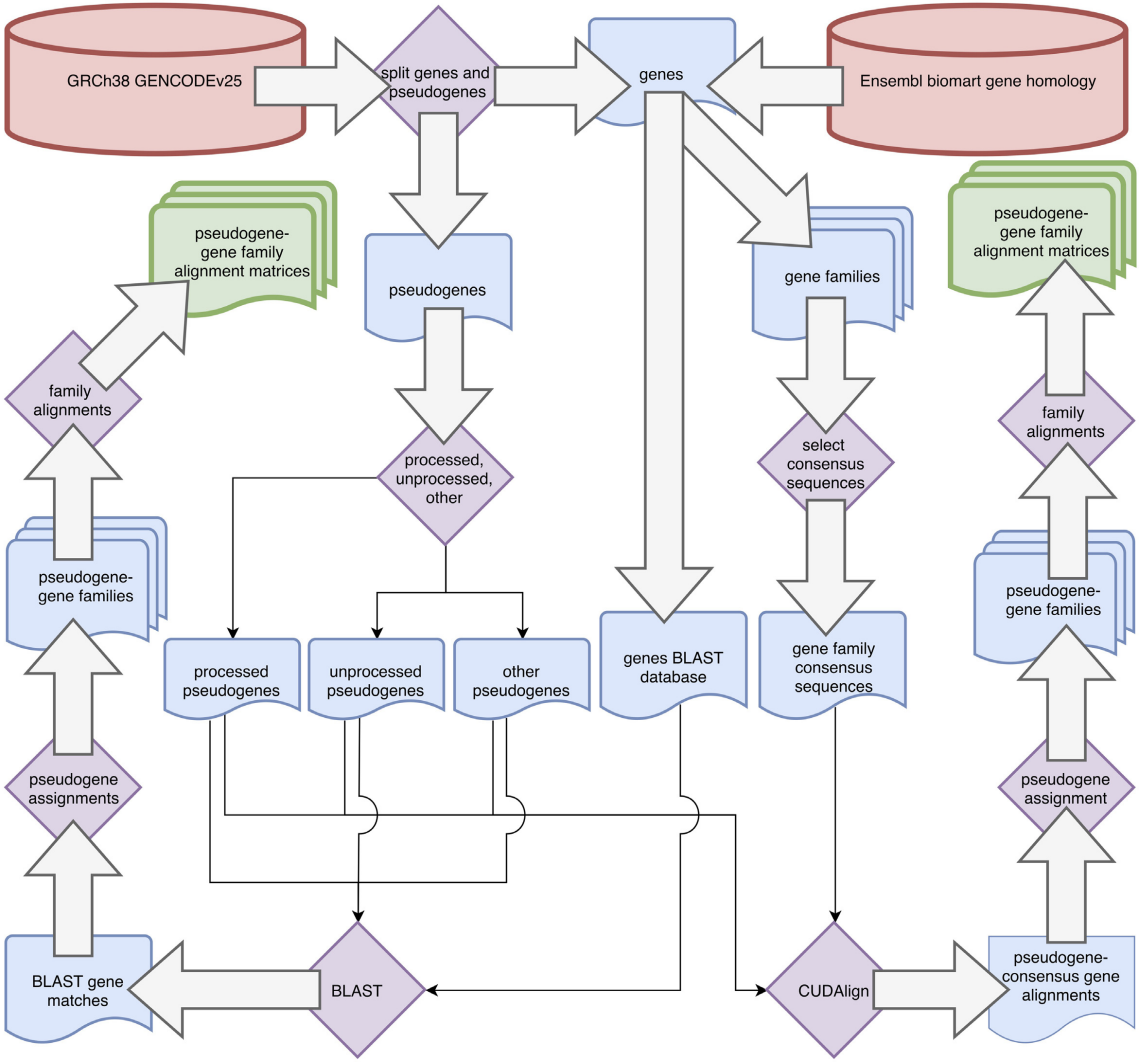
Workflow for both CUDAlign and BLAST databases. Left side PGG families are produced using the BLAST matches. Right side PGG families are produced using the PGG family alignment matrix with percentile cutoffs using CUDAlign.

To generate these gene families, we use two methods: (i) CUDAlign-based local alignment of pseudogenes against consensus sequences representing gene families and (ii) BLAST-based search of pseudogene sequences against all gene sequences (Fig. 1). These two approaches can be thought of as heuristic but different processes. The local sequence alignment approach is heuristic in that only two gene sequences are used from each gene family to reduce the search space. These sequences are the most similar and representative sequences to all the other gene sequences in the family. The BLAST-based approach is heuristic in that not all sequences are fully aligned during the process due to the seed-and-extend steps of BLAST [42]. The result is that not every relationship between pseudogene and gene family is recorded, which is an advantage in runtime but a disadvantage in studying underlying sequence homology.

#### CUDAlign-based local alignment of gene families

Gene homolog families were generated using the Ensembl biomart gene homolog database [43, 44]. The pairs of homologous genes were separated into connected components using the Python networkx package [45]. These connected component sub-graphs are considered gene families in this study. To reduce the number of alignments that needed to be performed, we selected consensus genes from each family that would be used to represent the entire family.

The consensus sequences were selected by aligning every member of the gene family to every other member using local alignment with CUDAlign [29]. The two members of the family with the largest sum alignment scores across all other family members were selected as the consensus sequences to increase the number of candidate sequences. If only one member existed in the family, then that member was the consensus sequence. Using the list of these consensus sequences we then aligned every consensus sequence to every pseudogene in the human genome GRCh38 annotated by GENCODE Release 25 (GENCODEv25) [46].

Specifically the pseudogenes are split up into processed, unprocessed, and other (unclear whether processed or unprocessed) on the basis of their mechanisms of formation [47]. We performed different alignment procedures for processed and unprocessed pseudogenes. The processed pseudogenes were aligned to all consensus gene transcripts with the highest local alignment score recorded. The unprocessed pseudogenes were aligned to the full genomic sequences of each of the consensus genes with the highest local alignment score recorded. Theoretically unprocessed pseudogenes can align to both exonic and intronic regions of DNA, while processed pseudogenes can only align to exonic regions. In our previous database we did not perform this two-procedure strategy in part to reduce the runtime of the problem [48]. These changes make the database much more complete and biologically relevant. The other pseudogenes were aligned to both the transcripts and the genomic sequence recording the highest score.

These scores, one for each combination of pseudogene to gene family, were stored for further analysis. Pseudogenes were assigned to families using a cutoff score (i.e., percentiles of the alignment scores per PGG alignment matrix) and a maximum number of assignments (i.e., the top four alignments above a cutoff). If greater than the top four alignments were used, the PGG families were too large to calculate the pairwise alignment matrix. The resulting sets of pseudogenes and genes are called PGG families. This method was used to allow a pseudogene to be assigned multiple families as well as prevent pseudogenes from being assigned families if their alignment score was low. We used the 99th percentile cutoff (corresponding alignment score 54), 99.9th percentile cutoff (135), and the 99.99th percentile cutoff (198) to generate three resultant databases named CUDAlign54, CUDAlign135, and CUDAlign198, respectively. A fourth database that is less stringent, CUDAlign18, is also included in the web applications using a 97.5th percentile cutoff (18). All these flavors of databases are available for search in our web applications.

#### BLAST-based generation of PGG families

In contrast to the local alignment of every combination of pseudogene to gene family, PGG families were also created by assigning the pseudogenes to the family containing its closest BLAST search match. This approach was used to contrast with the CUDAlign method, which uses up to the top four matches. The pseudogenes were separated into processed, unprocessed, and other. Then, all genes in the GENCODE Release 25 annotation were used to generate genomic, transcript, and combined BLAST databases (BlastDB). The processed pseudogenes would be BLAST searched against transcript BlastDB, unprocessed against the genomic sequence BlastDB, and the other pseudogenes were BLAST searched against the combined genomic/transcript BlastDB. The pseudogene was assigned to the gene family containing the best match from the BLAST search.

### Comparison between PGG families and pseudogene–parent gene pairs

We also conduct a comparison to the Pseudogene.org resource [49]. In this comparison, we consider pseudogenes and parent gene pairs from the Pseudogene.org psiDr [31] database (old) [50] and on GENCODE Release 10 from the Pseudogene.org psiCube [11] database (new) [51]. From our databases, we consider every combination of pseudogene to gene within a PGG family as a pair (e.g., a family with 3 genes and 2 pseudogenes would have $C_2^3 = \ 6$ pairs). Because we have multiple flavors of PGG databases including the BLAST-based version and the CUDAlign-based versions, we compare the intersections between two Pseudogene.org versions and our BLAST/CUDAlign-based versions. We show the intersections of PGG pairs in Venn diagrams.

### Development of PseudoFuN web applications

Aside from generating different flavors of the PGG databases, we assemble them into an online R Shiny application called PseudoFuN [36], which supports gene and pseudogene symbol queries against our PGG databases, generates dynamic networks, produces gene ontology [52] (GO) tables, and provides additional functional analysis features (Table 1). The functionalities, such as calculating the gene co-expression for any resultant PGG network in any of the TCGA [53] cancer types, are important for ceRNA network hypothesis generation in human cancers. More information can be found in the README file and tutorial on the PseudoFuN website.

**Table 1:** Summary of features that are freely available at the PseudoFuN website

PseudoFuN feature	Additional description
Interactive visualization of PGG family networks including the query pseudogene/gene	Users can query any single gene or pseudogene symbol, e.g., PTENP1. Nodes are colored by sub-clusters within the network.
Functional enrichment analysis of PGG family	Functional enrichment can be conducted on the genes within the PGG family on Biological Process, Molecular Function, or Cellular Components annotations. The GO functional enrichment is calculated with (i) Fisher exact test [55], (ii) Kolmogorov-Smirnov Classic [56], or (iii) Kolmogorov-Smirnov Elim [56].
Genomic loci mapping of PGG family	The genes in the PGG family can be mapped back to the genome using a circus plot to identify potential loci of interest.
Data download for all of the figures	Users can also download results including (i) the DPgE table for all pseudogenes in the selected cancer, (ii) the gene and pseudogene expression, (iii) miRNA correlation table.
Links to other gene databases for more information	By directly clicking the node in the network, users can open the GeneCards and Ensembl websites [43, 57] for detailed gene information.
Gene/pseudogene co-expression analysis across the entire TCGA	Once a PGG family has been identified the gene/pseudogene co-expression matrix is calculated across 1 of the 32 available TCGA cancer types.
Tumor vs normal DE of genes/pseudogenes across all TCGA cancer types	The gene/pseudogene DE is calculated for all members of the selected PGG family. There is also an option to run DE on a specified cancer for all pseudogenes, which can be viewed or downloaded as a table.
Predicted miRNA targets involved in the PGG families across all TCGA cancer types	The miRNA targets involved in the selected cancer and PGG family are displayed to show which miRNAs could regulate the PGG family members using the miRNA correlation tables from TCGA.
DPgE analysis	Differential pseudogene expression is calculated for each of the pseudogenes in TCGA cancers using dreamBase expression information [20]. The online tool allows for manipulation and download of the table.

DE: differential expression; DPgE: differential pseudogene expression.

Additionally we created another web application hosted by the OSC OnDemand [54] platform. This application has multiple functionalities including the query of Ensembl gene ID or a novel sequence against one selected flavor of our databases. For each of these features we provide a simple-to-use interface that allows users to select which database to query, allows download of the query hits, and allows users to interactively explore the PGG family networks including GO information.

### Use cases in multiple cancers

Furthermore, three use cases are provided to show the potential utility of PseudoFuN to researchers and oncologists looking for functional relationships between pseudogenes, genes, and miRNAs. Use Case I validates known PGG functional relationships. Use Case II identifies high-confidence novel miRNA-pseudogene-gene relationships. Use Case III is primarily focused on agreement with a validation study. We focused on pseudogenes/genes that were differentially expressed (DE) in low *RARG*/low *TACC1*/high *miR-96* compared to the reverse in prostate cancer cell lines and also DE in our PGG networks in TCGA prostate cancer samples.

## Results

### Local alignment of gene families

We performed 1.6 billion local alignments between all pseudogenes and all gene family consensus sequences. The process required >40,000 GPU hours on the Oakley cluster at the OSC. The highest scores for each gene family and pseudogene were stored in a 17,273 × 26,754 matrix of pseudogene-to-gene-family alignment scores (∼462 million elements). From this matrix, we are able to explore global PGG family homology relationships and assign pseudogenes to ≥1 gene families with high sequence homology.

**Figure 2:**
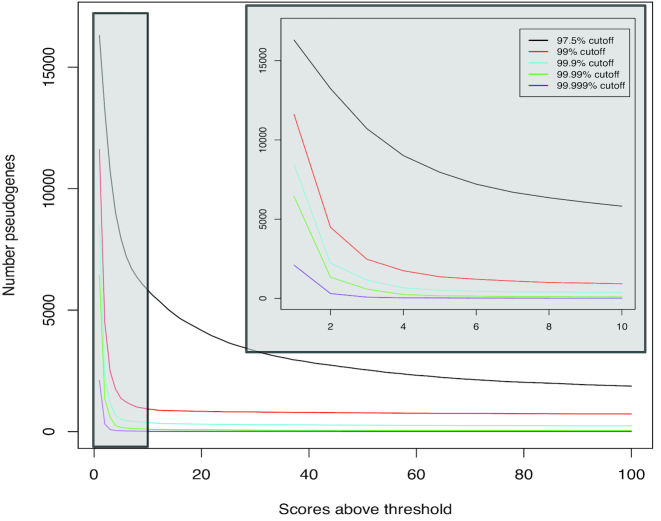
The number pseudogenes that align to gene families. The x-axis is the number of gene families, which have an alignment score above a specified cutoff (the different colored lines). The y-axis is the number of pseudogenes with an alignment score higher than the cutoff to the number of gene families on the x-axis. The inset gray box is a closer view of the low-range gene family numbers (1–10) to show higher-resolution patterns.

As one might expect, the number of pseudogenes with high alignments (defined as above a percentile threshold) to many gene families is relatively low. It can be seen that the majority of pseudogenes will align to one gene family in the CUDAlign databases (Fig.   2). We evaluate alignment of pseudogenes to genes using the Smith-Waterman local pairwise alignment score [58] between a pseudogene and a gene. These scores indicate the highest score possible for two sequences based on their specific dynamic programing matrix, which is solved by the Smith-Waterman algorithm. The cutoffs we use, 18, 54, 135, and 198, indicate the 97.50th, 99.0th, 99.90th, and 99.99th percentiles of alignment scores in our alignment matrix between all pseudogenes and consensus sequences. Another feature of note is that there are some pseudogenes that align to many gene families (e.g., nine pseudogenes, *UBE2Q2P1*, *RP11-313J2.1*, *TPTEP1*, *BMS1P1*, *CTD-2245F17.3*, *SCAND2P*, *GTF2IP7*, *WHAMMP3*, and *IGLV3-2*, have alignment scores >54 in 15,000 gene families and 571 pseudogenes [see Supplementary Table 2] have alignment scores >54 in 1,000 gene families).

**Table 2:** Benchmarking analysis of PseudoFuN databases

Gene	BlastDB	CUDAlign18	CUDAlign54	CUDAlign135	CUDAlign198	PMID
*PTEN*	Yes	No	No	No	No	26442270
*TUSC*	No	No	No	No	No	26442270
*INTS6*	Yes	No	No	No	No	26442270
*OCT4*	Yes	Yes	Yes	Yes	Yes	26442270
*HMGA1*	Yes	Yes	Yes	Yes	Yes	26442270
*CYP4Z1*	No	No	No	No	No	26442270
*BRAF*	Yes	No	No	No	No	26442270
*KLK4*	No	No	No	No	No	22726445
*ATP8A2*	No	Yes	Yes	No	No	22726445
*CXADR*	No	Yes	Yes	Yes	Yes	22726445
*CALM2*	Yes	Yes	Yes	Yes	Yes	22726445
*TOMM40*	Yes	Yes	Yes	Yes	Yes	22726445
*NONO*	Yes	Yes	Yes	Yes	Yes	22726445
*PERP*	No	Yes	Yes	Yes	Yes	22726445
*DUSP8*	Yes	Yes	No	No	No	22726445
*YES1*	Yes	Yes	No	No	No	22726445
*GJA1*	Yes	No	No	No	No	22726445
*AURKA*	Yes	Yes	Yes	Yes	Yes	22726445
*RHOB*	No	No	No	No	No	22726445
*HMGB1*	Yes	Yes	Yes	Yes	Yes	22726445
*EIF4A1*	Yes	Yes	No	No	No	22726445
*EIF4H*	Yes	Yes	Yes	Yes	Yes	22726445
*SNRP6*	Yes	Yes	Yes	Yes	Yes	22726445
*RAB1*	Yes	No	No	No	No	22726445
*VDAC1*	Yes	Yes	No	No	No	22726445
*RCC2*	Yes	No	No	No	No	22726445
*PTMA*	Yes	Yes	Yes	Yes	Yes	22726445
*NDUFA9*	Yes	Yes	Yes	Yes	Yes	22726445
*CES7*	Yes	No	No	No	No	22726445
*EPCAM*	Yes	Yes	Yes	Yes	Yes	22726445
*FTH1*	Yes	Yes	Yes	Yes	Yes	29240947
Hits	24/31	20/31	16/31	15/31	15/31	
Total hits	27/31					

Genes indicate the gene with which the pseudogenes are associated in the literature. BLAST and CUDAlign columns indicate the specific databases. PMID indicates the literature from which the PGG relationship was derived. Benchmark totals are included at the bottom of the table.

In contrast to previous belief in single PGG homology, some pseudogenes are related to many genes. It is worth considering that these high-homology pseudogenes (e.g., *FTLP10* with 3,006 gene family pairwise alignments over a 54 threshold) may play a role in regulating major biological processes [59] and disease [60]. Of the nine highest homology pseudogenes (Supplementary Table 2), one, *RP11-313J2.1*, is a zinc finger pseudogene and two, *CTD-2245F17.3* and *SCAND2P*, are located in the promoters of zinc finger genes. Four pseudogenes in the nine highest homology pseudogenes ( *RP11-313J2.1*, *CTD-2245F17.3*, *SCAND2P*, and *WHAMMP3*) also have 92–96% sequence identity with zinc finger genes (*ZNF72P*, *ZNF518A*, *ZNF37A*, and *ZNF788P*/ *ZNF20*, respectively) when BLAST searched against the human genome. Of the 571 highest homology pseudogenes (Supplementary Table 2), we found 27 zinc finger pseudogenes. Using EnrichR [61] we identified enrichment in GO Molecular Function GO:0 004430 1-phosphatdylinositol 4-kinase activity (Fisher exact test *P*-value = 0.001), and enrichment for GO Biological Process GO:00 70475 rRNA base methylation (Fisher exact test *P*-value = 0.003). In the ARCHS4 database [62] 324 transcription factors were significantly co-expressed (Benjamini-Hochberg adjusted Fisher exact test *P*-value < 0.05) with members of the 571 highest homology pseudogenes. Of those 324 transcription factors, 228 were zinc finger genes. These findings show that the highest homology pseudogenes, like zinc finger genes, likely contain repetitive elements that align to many genomic loci.

### BLAST generation of PGG families

The BLAST-generated database was larger than the CUDAlign-generated databases, with 68,578 total connections. This database was also much simpler to compute because it was not an exhaustive search. These conclusions make it a simple method for quickly estimating the pseudogene-to-gene relationships.

### Direct comparison to pseudogene parents

**Figure 3:**
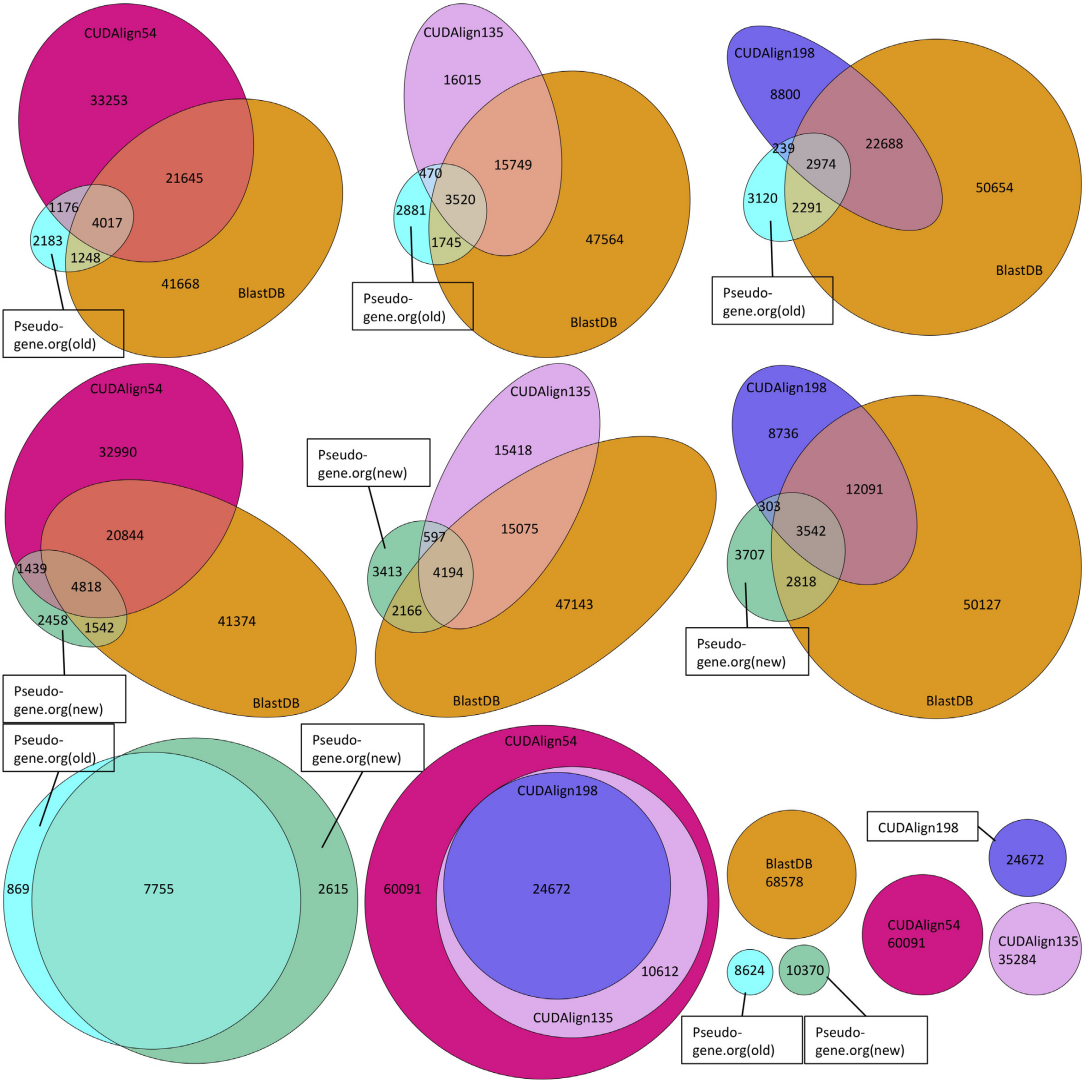
Comparison of database members. The top six plots are comparisons between the CUDAlign databases using different cutoffs, the BLAST database, and the Pseudogene.org parent genes. The bottom row shows intra-database comparisons, left: Pseudogene.org, middle: CUDAlign database of different alignment score cutoffs, right: relative size of all databases.

We compare our databases to the previous pseudogene–parent gene databases retrieved from Pseudogene.org resources (Fig. 3). This shows that our methods reconstruct most of the pseudogene–parent gene relationships identified by Pseudogene.org. The overall consistency of our databases (BLAST and CUDAlign) with both Pseudogene.org databases (new and old) was 75% (i.e., all our databases combined). Individually, the BLAST-based database contained 61% of the Pseudogene.org relationships (both new and old) and the CUDAlign 54 cutoff contained 60% of the Pseudogene.org relationships (both new and old). Our databases also generated a larger pool of possible interactions. It is worth noting that 391 pseudogenes and 152 genes in the new Pseudogene.org (GENCODE Release 10) are not present in the GENCODE Release 25 annotation used in our analysis. These genes and pseudogenes together account for 1,030 edges that were used in our comparison. Accounting for these differences in the annotation, we are able to reconstruct 85% of the PGG relationships in the new Pseudogene.org database. Because these associations were generated without prior PGG relationship information and the annotations have changed slightly since Pseudogene.org, our methods prove to independently identify known and unknown PGG relationships at a high rate.

### Development of a pseudogene query tool

The R Shiny application is a comprehensive hypothesis-generating tool that is freely available on the internet [36]. This tool provides a wide array of functionality that a researcher can access quickly and download results as the raw data for more in-depth analysis. These features are outlined in detail in Table 1.

*Use cases: Assisting functional study of ceRNA networks in cancer*

To illustrate the utility of our databases and tools we present three use cases.

Use Case I: to validate known PGG relationships, we first identified 31 benchmark PGG relationships from three studies [ 15, 16, 23] and query our databases. These studies represent prominent regulatory pseudogenes in cancers by established laboratories. We query a gene/pseudogene name one at a time, and PseudoFuN will return the top PGG network(s) that contain the query (Table   2). In general, we found that our databases together were able to identify 87% of the benchmarking cases (Table 2) and the CUDAlign versions were able to identify 65% of the benchmarking cases. Perhaps most importantly, three of the cases identified by CUDAlign ( *ATP8A2*, *CXADR*, *PERP*) were not identified by the more traditional BLAST approach (Table 2), showing that consensus sequence alignment can identify some overlooked relationships. Next, individual benchmark cases were evaluated in more detail (Supplementary Fig. 2).

*PTENP1* is a processed pseudogene homologous to *PTEN*, a tumor suppressor gene. *PTENP1* is selectively lost in cancer and may regulate *PTEN* expression as a miRNA decoy target [5, 6]. We have observed differential co-expression patterns of PGG families in tumor vs normal tissue for *PTENP1* network in multiple cancers including breast cancer (Supplementary Fig. 3B and C). We identified known miRNAs (*hsa-miR-93* targets *PTEN* in breast cancer [63]) targeting *PTEN* PGG network nodes, providing insights into ceRNA regulation (Supplementary Fig. 3D). These insights are important because some pseudogenes competitively bind to miRNAs and thus regulate gene expression. We also identify *hsa-miR-103a-3p*, known to regulate *PTEN* in endometrial [64] and colorectal cancers [65], in breast cancer (Supplementary Fig. 3D). The miRNA *hsa-miR-20a*, known to regulate *PTEN* by the ceRNA mechanism in prostate cancer [66], was also identified in breast cancer. The ceRNA network regulatory relationship is governed by effect modulation of miRNA on gene expression by pseudogene expression (Supplementary Fig. 1A, C, E). This leads to a correlation between pseudogene (miRNA decoy targets) and gene (miRNA targets) expression (Supplementary Fig. 1D), where pseudogenes and homologous genes competitively bind to miRNAs. *KRAS*-*KRASP1* regulatory network was also identified by our database (Supplementary Fig. 2). *KRAS* and *KRASP1* are known to be involved in ceRNA network regulation [5, 10, 66]. PseudoFuN query of *KRAS* identified co-expression patterns in prostate cancer consistent with ceRNA network regulation by *hsa-miR-145*, a known modulator of *KRAS* in prostate cancer [67]. The *FTH1* query also resulted in the identification of pseudogenes (*FTH1P2*, *FTH1P8*, *FTH1P11*, *FTH1P16*) that regulate *FTH1* in prostate cancer [23] as well novel miRNAs that may be involved in ceRNA network regulation of *FTH1* in prostate cancer. *GBP1* is an IFN-α–induced transcript that is involved in immune response in prostate cancer [68]. The *GBP1*-involved PGG network also contained the pseudogene *GBP1P1*, which may have a ceRNA regulatory role in breast cancer [69] and in some neurodegenerative diseases [70].

Use Case II: We wanted to identify possible gene-miRNA relationships of interest within our database. We chose to study these relationships with respect to miR-96, a known cancer regulator miRNA in prostate cancer [71]. Through DE analysis between tumors in the TCGA-PRAD cohort with lower expression of *RARG* and *TACC1* (also a *miR-96* target) and high expression of *miR-96* (low *RARG*/low *TACC1*/high *miR-96*), compared to the reverse, we previously identified that altered *SOX15* gene expression is significantly associated with worse disease-free survival. We visualized expression patterns of *SOX15* PGG families, and corresponding miRNA associations, where *miR-96* is included as a validation.

**Figure 4:**
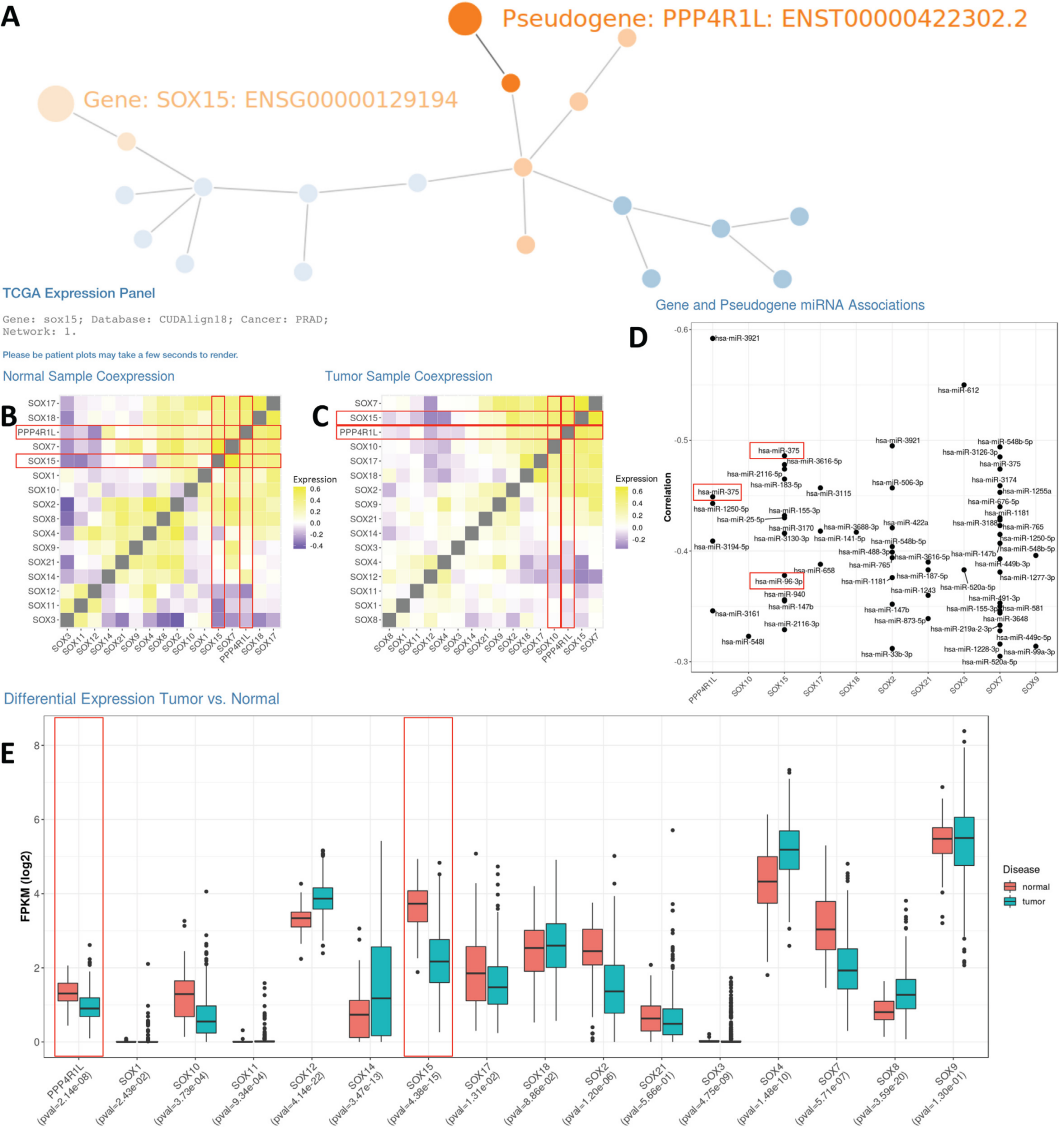
PseudoFuN online output for *SOX15* PGG family. A, Interactive graph visualization of the *SOX15* PGG network. B, TCGA prostate co-expression matrix for *SOX15* PGG family genes and pseudogenes across normal samples. C, TCGA prostate co-expression matrix for *SOX15* PGG family genes and pseudogenes across tumor samples. D, Negatively correlated miRNAs for all members of the *SOX15* PGG family. E, Differential gene and pseudogene expression for tumor and normal samples for each member of the *SOX15* PGG family in the prostate cancer TCGA dataset. FPKM: fragments per kilobase million.

Interestingly we identified the pseudogene *PPP4R1L* as a potential member of a *SOX15* ceRNA network (Fig. 4A). *PPP4R1L* and *SOX15* are both significantly DE between tumor and normal controls (Bonferroni-corrected *P*-value = 3.42 × 10^−7^, 2.01 × 10^−14^, respectively; Fig. 4E). *PPP4R1L* and *SOX15* are significantly co-expressed (Pearson correlation coefficient = 0.51, *P*-value < 2.2 × 10^−16^) in tumor tissue but much less correlated in normal controls in prostate cancer (Pearson correlation coefficient = 0.24, *P*-value = 0.09; Fig. 4B and C). Positively correlated expression is an assumption when determining ceRNA network relationships [72] (Supplementary Fig. 1). Both *SOX15* and *PPP4R1L* are likely regulated by *hsa-miR-375* based on the TCGA prostate cancer dataset. *hsa-miR-375* is associated with docetaxel resistance in prostate cancer [73, 74] and *PPP4R1L* knock-down in HeLa cells induces taxol resistance [75]. These findings are intriguing because taxol and docetaxel are closely related chemical compounds. *PPP4R1L* is also located in a region associated with high mutation rates in cancer cell lines [75], which could be indicative of mutational “on/off switches” in pseudogene regulation.

Use Case III: We were most interested in the DE genes (and related pseudogenes) that both appeared in our PGG database and were contained in networks with genes DE in low *RARG*/low *TACC1*/high *miR-96* compared to vice versa. We searched the DE genes in our PGG database and identified the top networks with enriched number of DE genes. As a result, parent genes *HTR7*, *CNN2*, *MSN*, and *TAGLN2* are DE; they generate pseudogenes, which are specifically expressed in prostate cancer samples [16]. These four parent genes are also detected in our five top PGG families involving *miR-96*–regulated (direct or indirect) DE genes. We identified *HTR7P1* pseudogene in the same PGG family as *HTR7* gene, which is potentially regulated by *hsa-miR-607* and *hsa-miR-3654* in the TCGA prostate cancer dataset (Supplementary Fig. 4). Eleven *CNN2* pseudogenes (*CNN2P1*-*CCN2P4*, *CNN2P6*-*CNN2P12*) were identified in the *CNN2* PGG family along with *TAGLN2* and *TAGLN2P1*. *TAGLN2P1*is DE between the tumor and normal samples in the prostate dataset (Supplementary Fig. 5; Bonferroni-corrected *P*-value = 6.23 × 10^−4^). *MSN* and *MSNP1* were in the same PGG family and *hsa-miR-96* potentially regulates *MSN* in the TCGA prostate cancer dataset (Supplementary Fig. 5). In addition, although our DE genes were detected from prostate cancer, we further compared them with DE pseudogenes identified in four other cancer types and we observed interesting results (see Supplementary Materials—Potential regulatory roles in cancer).

## Discussion

We identify 133,770 PGG families that have significant potential to reveal important information about regulatory PGG relationships in health and disease. Within these families we identify both new and existing regulatory networks that contain pseudogenes such as *PTENP1*, *KRAS1P*, *FTH1P8*/*11*/*16*, and *GBP1P1* (Fig. 4). Because all genes and all pseudogenes are included in our database, there are thousands of opportunities to identify new regulatory relationships. These thousands of opportunities can be easily stratified using gene name, pseudogene name, and cancer type. Our PseudoFuN web application makes it a simple and intuitive process to query pseudogenes (or genes) to identify which gene families they may be regulating as well as the functions that are attributed to the members of the network. We also have an application hosted by the OSC that allows the querying of novel sequences against our database.

From these networks, we can also identify possible relationships of DE pseudogenes in various cancers. For instance, both *PPP4R1L* pseudogene and *SOX15* are DE in prostate cancer and associated with *hsa-miR-375*. These types of relationships should be further evaluated along with more complex regulation with multiple miRNAs, pseudogenes, and genes. It is experimentally shown that *SOX15*is regulated by *hsa-miR-96* [71] and it may be important to include *hsa-miR-96* in the *hsa-miR-375*-*SOX15*-*PPP4R1L* potential ceRNA network. Aside from PGG family-specific differential pseudogene expression, the PseudoFuN application allows for comprehensive differential pseudogene expression (DPgE) analysis in any of the TCGA cancer datasets.

The use of this database also has utility in integrative analysis where the databases can be used as a mask for other data modalities. Some examples would be using the nodes (genes and pseudogenes) in each of the PGG families as groups in gene expression experiments. Similarly, these groups could be used for feature reduction when visualizing data. We hope researchers can use these relationships we have identified to reduce large numbers of candidate associations down to numbers that can be easily validated and generate new candidates when querying novel sequences. For instance, miRNA-gene pairs filtered through the sets of PGG families would identify high-priority ceRNA candidates.

## Conclusions

We generate multiple large databases of PGG family relationships and the tools to study them for use by biomedical researchers. These databases are more comprehensive than previous PGG databases by including many more homology relationships in PGG families, thus more powerful for experiment validation and knowledge discovery. These databases are useful in identifying PGG regulatory relationships in 32 cancer types and show high similarity with known PGG relationships. Aside from the known relationships we identify many unknown relationships. Furthermore, these databases and associated analyses can be easily accessed online or through the OSC OnDemand platform, allowing for novel hypotheses to be assessed quickly by biomedical researchers. We find evidence of both known regulatory PGG relationships and novel hypothesized relationships that we plan to validate. PseudoFuN is a comprehensive, dynamic tool that allows any bioinformatician or oncologist to find novel regulatory pseudogenes within their cancer or gene network of interest.

## Availability of supporting data and materials

We have made the PGG family data publicly downloadable from GitHub [35]. We also created an R Shiny web application called PseudoFuN [36] that supports querying the PGG databases, interactive visualization and functional analysis of the PGG networks, and visualization of PGG co-expression and miRNA binding. Apache License 2.0 is associated with PseudoFuN (R Shiny web application). These data are also available in GigaDB [76]. In addition, we provide another interactive web application hosted on Ohio Supercomputer Center (OSC) OnDemand, which supports querying novel sequences against any of our PGG databases and visualization of the resulting PGG networks.

## Availability of supporting source code and requirements

Project name: PseudoFuN

Project home page: https://github.com/yanzhanglab/PseudoFuN_app, https://github.com/OSC/pseudofun, https://integrativeomics.shinyapps.io/pseudofun_app/

Operating system: platform independent

Programming language: R, Python, JavaScript

Other requirements: not applicable

License: CC, MIT

RRID:SCR_017095

OSC OnDemand application access: contact yan.zhang@osumc.edu.

## Additional files

There is an additional Supplementary Materials file containing additional information on the data and additional analyses. It includes the following figures and tables:

**Supplementary Figure 1**. Example of ceRNA network regulation of gene expression. A, A graphical view of how pseudogene expression can regulate gene expression. B, A cellular view of ceRNA network regulation. C, Equations used to model the correlation between gene and pseudogene expression in a ceRNA network. D, The distribution of the PGG correlations based on the models in C. E, The effect that pseudogene expression has on the miRNA-induced change in gene expression.

**Supplementary Figure 2**. PseudoFuN online output for *PTEN* PGG family. A, Interactive graph visualization of the *PTEN* PGG network. B, TCGA prostate co-expression matrix for *PTEN* PGG family genes and pseudogenes across normal samples. C, TCGA prostate co-expression matrix for *PTEN* PGG family genes and pseudogenes across tumor samples. D, Negatively correlated miRNAs for all members of the *PTEN* PGG family. E, Differential gene and pseudogene expression for tumor and normal samples for each member of the *PTEN* PGG family in the prostate cancer TCGA dataset.

**Supplementary Figure 3**. PseudoFuN online output for *HTR7* PGG family. A, Interactive graph visualization of the *HTR7* PGG network. B, TCGA breast cancer co-expression matrix for *HTR7* PGG family genes and pseudogenes across normal samples. C, TCGA breast cancer co-expression matrix for *HTR7* PGG family genes and pseudogenes across tumor samples. D, Negatively correlated miRNAs for all members of the *HTR7* PGG family in breast cancer. E, Differential gene and pseudogene expression for tumor and normal samples for each member of the *HTR7* PGG family in the breast cancer TCGA dataset.

**Supplementary Figure 4**. PseudoFuN online output for *CNN2*/*TAGLN2* PGG family. A, Interactive graph visualization of the *CNN2*/*TAGLN2* PGG network. B, TCGA prostate co-expression matrix for *CNN2*/*TAGLN2* PGG family genes and pseudogenes across normal samples. C, TCGA prostate co-expression matrix for *CNN2*/*TAGLN2* PGG family genes and pseudogenes across tumor samples. D, Negatively correlated miRNAs for all members of the *CNN2*/*TAGLN2* PGG family. E, Differential gene and pseudogene expression for tumor and normal samples for each member of the *CNN2*/*TAGLN2* PGG family in the prostate cancer TCGA dataset.

**Supplementary Figure 5**. PseudoFuN online output for *MSN* PGG family. A, Interactive graph visualization of the *MSN* PGG network. B, TCGA prostate co-expression matrix for *MSN* PGG family genes and pseudogenes across normal samples. C, TCGA prostate co-expression matrix for *MSN* PGG family genes and pseudogenes across tumor samples. D, Negatively correlated miRNAs for all members of the *MSN* PGG family. E, Differential gene and pseudogene expression for tumor and normal samples for each member of the *MSN* PGG family in the prostate cancer TCGA dataset.

**Supplementary Figure 6**. The PGG families in our network with the most DE genes after *miR-96* treatment. The line weights indicate the sequence homology between members of the PGG family. Red nodes indicate *miR-96* targets. Yellow nodes with names indicate other genes contained in the PGG family. Yellow nodes without names are pseudogenes contained within the network.

**Supplementary Figure 7**. The user interface of the OSC OnDemand web application. A, Main query page where a user can search either sequences or Ensembl gene IDs. B, Representative output of one of the gene searches. This includes an interactive network and the GO information.

**Supplementary Figure 8**. *GBP1P1* DE in TCGA prostate cancer (information retrieved from Han et al.).

**Supplementary Table 1**. DE parent gene/pseudogenes potentially regulated by *miR-96* in prostate cancer vs TCGA-derived DE pseudogenes.

## Abbreviations

BLAST: Basic Local Alignment and Search Tool; ceRNA: competing endogenous RNA; DE: differential expression/differentially expressed; DPgE: differential pseudogene expression; FPKM: fragments per kilobase million; GO: gene ontology; GPU: graphics processing unit; miRNA: microRNA; OSC: Ohio Supercomputer Center; PGG: pseudogene-gene; PseudoFuN: Pseudogene Functional Networks; TCGA: The Cancer Genome Atlas.

## Competing interests

The authors declare that they have no competing interests.

## Funding

This work is partially supported by NIH-NLM MIDAS Training Fellowship (4T15LM011270-05) and NIH-NLM Ruth L. Kirschstein Predoctoral Individual National Research Service Award (1F31LM013056-01) awarded to Travis Johnson. It is also supported by The Ohio State University Startup Funds and OSU Comprehensive Cancer Center Support Grant (P30CA016058) to Yan Zhang.

## Author contributions

T.S.J., S.L., Z.H., and Y.Z. performed data analyses. T.S.J., E.F., and Z.H. developed the web applications. Y.Z. and T.S.J. conceived and initiated this project. Y.Z. and K.H. supervised the project. M.J.C. provided experimental data. All authors contributed to biological interpretation. T.S.J., Y.Z., M.J.C., and S.D.L. wrote the manuscript. All authors read and approved the manuscript.

## Supplementary Material

GIGA-D-18-00369_Original_Submission.pdf

GIGA-D-18-00369_Revision_1.pdf

GIGA-D-18-00369_Revision_2.pdf

Response_to_Reviewer_Comments_Original_Submission.pdf

Response_to_Reviewer_Comments_Revision_1.pdf

Reviewer_1_Report_Original_Submission -- Cameron Bracken 10/21/2018 Reviewed

Reviewer_2_Report_Original_Submission -- Jennifer Harrow 11/10/2018 Reviewed

Reviewer_2_Report_Revision_1 -- Jennifer Harrow 2/10/2019 Reviewed

Supplemental File
