# Supplementary material for: PseudoFuN: Deriving functional potentials of pseudogenes from integrative relationships with genes and microRNAs across 32 cancers
Source: Gigascience. 2019 Apr 26;8(5):giz046. doi: 10.1093/gigascience/giz046 (PMC6486473; doi:10.1093/gigascience/giz046)
Supplement: GIGA-D-18-00369_Revision_1.pdf [file giz046_giga-d-18-00369_revision_1.pdf]

# GigaScience

# PseudoFuN: Deriving functional potentials of pseudogenes from integrative relationships with genes and miRNAs across 32 cancers

--Manuscript Draft--

| | |
|---|---|
| **Manuscript Number:** | GIGA-D-18-00369R1 |
| **Full Title:** | PseudoFuN: Deriving functional potentials of pseudogenes from integrative relationships with genes and miRNAs across 32 cancers |
| **Article Type:** | Technical Note |
| **Funding Information:** | U.S. National Library of Medicine (US) (4T15LM011270-05) — Mr. Travis Johnson<br>Ohio State Univerisy (Startup Funds) — Dr. Yan Zhang |
| **Abstract:** | Background: Long thought “relics” of evolution, not until recently have pseudogenes been of medical interest regarding regulation in cancer. Often, these regulatory roles are a direct byproduct of their close sequence homology to protein coding genes. Novel pseudogene-gene functional associations can be identified through the integration of biomedical data, such as sequence homology, functional pathways, gene expression, pseudogene expression, and miRNA expression. However, not all of the information has been integrated, and the vast majority of previous pseudogene studies relied on 1:1 pseudogene-parent gene relationships without leveraging other homologous genes/pseudogenes. Results: We produce pseudogene-gene (PGG) families that expand beyond the current 1:1 paradigm. Firstly, we construct expansive PGG databases by i) CUDAlign GPU accelerated local alignment of all pseudogenes to gene families (totaling 1.6 billion individual local alignments and more than 40,000 GPU hours) and ii) BLAST-based assignment of pseudogenes to gene families. Secondly, we create an open-source web application (PseudoFuN) to search for integrative functional relationships of sequence homology, miRNA expression, gene expression, pseudogene expression, and gene ontology. We produce four “flavors” of databases (>462,000,000 pseudogene-gene pairwise alignments and 133,770 PGG families) that can be queried and downloaded using PseudoFuN. These databases are consistent with previous 1:1 pseudogene-gene annotation and also are much more powerful including millions of de novo pseudogene-gene associations. For example, we find multiple known (e.g., miR-20a-PTEN-PTENP1) and novel (e.g., miR-375-SOX15- PPP4R1L) miRNA-gene-pseudogene associations in prostate cancer. PseudoFuN provides a “one stop shop” for identifying and visualizing thousands of potential regulatory relationships related to pseudogenes in TCGA cancers. Conclusions: Thousands of new pseudogene-gene associations can be explored in the context of miRNA-gene-pseudogene co-expression and differential expression with a simple-to-use online tool by bioinformaticians and oncologists alike. |
| **Corresponding Author:** | Yan Zhang<br>Ohio State University<br>Columbus, Ohio UNITED STATES |
| **Corresponding Author Secondary Information:** | |
| **Corresponding Author's Institution:** | Ohio State University |
| **Corresponding Author's Secondary Institution:** | |
| **First Author:** | Travis Johnson |
| **First Author Secondary Information:** | |
| **Order of Authors:** | Travis Johnson |
| | Sihong Li |
| | Eric Franz |
| | Zhi Huang |

Powered by Editorial Manager® and ProduXion Manager® from Aries Systems Corporation

| | |
|---|---|
| | Shuyu Dan Li |
| | Moray J Campbell |
| | Kun Huang |
| | Yan Zhang |
| **Order of Authors Secondary Information:** | |
| **Response to Reviewers:** | Response Letter (We recommend reading the pdf version Giga_reviews_20181212.pdf for clearer format.)<br><br>We thank the reviewers for their insightful comments and believe that after addressing each comment the manuscript is stronger. Please see the reviewers' comments and our responses below to see specifically how all of the concerns were addressed. We have highlighted all our answers in red color. We also highlight in red color all the changes in the main text.<br><br>Reviewer #1: The role of pseudogenes in the modulation of gene regulation is a burgeoning field that is ideally placed to benefit from integrative approaches that utilise "big data" that is currently available. A user friendly tool such as PseudoFun is therefore of use as a possible discovery mechanism for new relationships. Not having used PsuedoFun at this stage, it is difficult to fully evaluate its performance, though the approach described appears useful and the presentation of new relationships such as that suggested between PPP4RiL, SOX15 and miR-375 highlight a potential to identify new avenues for further investigation. I have only minor suggestions for improvement in presentation.<br>1) In Figure 5 (and much of the supplementary figures presented in a similar fashion), is the miRNA associated directly targeting the gene/pseudogene. Visually, only a correlative expression relationship is indicated.<br><br>Answer: We appreciate the reviewer's feedback. In Figure 5 (currently Figure 4), the miRNAs associated with gene/pseudogene were determined by not only expression correlation but also miRNA target prediction databases: Miranda, PicTar and TargetScan. We downloaded the predictions from http://gdac.broadinstitute.org. According to the reviewer's comment, we have improved the miRNA section on the TCGA Expression panel of the website. The website now allows the user to select how many algorithms predict regulation of the gene/pseudogene by the miRNA. This value is used as a threshold for the displayed miRNAs. The default is 0 meaning that the miRNA and gene/pseudogene are significantly negatively correlated indicative of possible regulation. The value can be changed from 0-3 indicating the number of algorithms (Miranda, PicTar, and TargetScan) predict regulation of gene/pseudogene by the specified miRNA.<br><br>2) Figure 4 does little to add clarity. If the goal is to highlight regulatory relationships, the ENSTxxx labelling does not lend for easy interpretation and the miRNAs are not shown. If the intended purpose is to illustrate a style at which data is outputted, perhaps this is better served by a user friendly series of screenshots illustrating a beginning to end data query - result flow?<br><br>Answer: We agree with the reviewer that containing only ENSTxxx labelling does not facilitate illustration. We use easy-to-interpret gene names and links to other gene databases (e.g. GeneCards, Ensembl) to improve the usability of our PseudoFuN website (https://integrativeomics.shinyapps.io/pseudofun_app/), and we have improved the visualization according the reviewers' suggestions. The old Figure 4 is not from our public PseudoFuN version, instead it is from our supercomputer version located on the Ohio Supercomputer Center clusters and is meant mainly for research purposes. Since it is not exactly the version we mainly presented in the main text, which has much more user-friendly interface with interpretable gene names, we moved the old Figure 4 to the supplementary materials so that it does not detract from the usability of the main application freely available online.<br><br>3) In Figure 5 and some supplementary figures, co-expression visually is not well represented by the colour scheme. ie: the tumour relationship between PPP4R1L and SOX15. The stats support this, the visual representation less so. Perhaps blanking out |

the 1:1 same gene : same gene diagonal would allow re-setting of the colour scheme to better represent co-expression?

Answer: This insightful comment by the reviewer caused us to rethink our visualization. We changed the same gene/pseudogene correlation (1.0) along the diagonal line in the heatmap to NaN so that the visualization ignores those values. This allows the range of color for the other correlations to be more diverse and more informative to the users.

4) In paragraph 2 of the results, I was unclear what the "alignment score above 54" means... What degree of alignment is this? I found understanding this to be hard to gauge. Relating to this, could the authors comment more extensively on their findings of tremendous levels of alignment for some pseudogenes?

Answer: We agree with the reviewer that we should more fully explain the alignment scores in the manuscript and as a result explain in more detail what the alignment scores represent in paragraph 2 of the results:
“We evaluate alignment of pseudogenes to genes using the Smith-Watermann local pairwise alignment score56 between a pseudogene and a gene. These scores indicate the highest score possible for two sequences based on their specific dynamic programing matrix which is solved by the Smith-Watermann algorithm. The cutoffs we use, 18, 54, 135, and 198, indicate the 97.50th, 99.0th, 99.90th and 99.99th percentiles of alignment scores in our alignment matrix between all pseudogenes and consensus sequences.”

We have also performed more in-depth analysis on the high homology pseudogenes and described these findings in more detail in paragraph 2-3 of Results. Specifically, we found zinc finger pseudogenes and other domain binding patterns in the highest homology pseudogenes. We found large bodies of evidence that the high homology pseudogenes have either direct or indirect relationships with zinc finger genes.
In Results paragraph 2:
“Another feature of note is that there are some pseudogenes that align to many gene families (e.g., 9 pseudogenes, UBE2Q2P1, RP11-313J2.1, TPTEP1, BMS1P1, CTD-2245F17.3, SCAND2P, GTF2IP7, WHAMMP3, IGLV3-2, have alignment scores above 54 in 15,000 gene families and 571 pseudogenes, see Supplementary Table 2, have alignment scores above 54 in 1,000 gene families).”
In Results paragraph 3:
“Of the 9 highest homology pseudogenes (Supplementary Table 2), one, RP11-313J2.1, is a zinc finger pseudogene and two, CTD-2245F17.3 and SCAND2P, are located in the promoters of zinc finger genes. Four pseudogenes in the 9 highest homology pseudogenes (RP11-313J2.1, CTD-2245F17.3, SCAND2P, and WHAMMP3) also have 92-96% sequence identity with zinc finger genes (ZNF72P, ZNF518A, ZNF37A and ZNF788P/ZNF20 respectively) when BLAST searched against the human genome. Of the 571 highest homology pseudogenes (Supplementary Table 2), we found 27 zinc finger pseudogenes. Using EnrichR59 we identified enrichment in GO Molecular Function GO:0004430 1-phosphatdylinositol 4-kinase activity (Fisher’s exact test p-value = 0.001), and enrichment for GO Biological Process GO:0070475 rRNA base methylation (Fisher’s exact test p-value = 0.003). In the ARCHS4 database60 324 transcription factors were significantly co-expressed (Benjamini-Hochberg adjusted Fisher’s exact test p-value < 0.05) with members of the 571 highest homology pseudogenes. Of those 324 transcription factors, 228 were zinc finger genes. These findings show that the highest homology pseudogenes, like zinc finger genes, likely contain repetitive elements that align to many genomic loci.”

Reviewer #2: The authors have presented an overview of their new analysis and data resource to identify novel pseudogene-gene network interactions that could lead to new hypothesis around their role in regulation of cancer using TCGA cancer expression data and miRNA expression. The unique element of this analysis is using a consensus sequence representing gene families and examining the local alignment of pseudogenes against this consensus to identify new potential interactions.

The major criticism of the paper is that a thorough benchmarking evaluation of their different alignment cutoffs has not been clearly presented, to guide the user when interpreting the network data and deciding which pseudogene appearing in the

different networks is worth looking into more depth or reject as being a  false positive result. This probably could be done with their validated use cases example taken for the literature such as PTEN /PTENP1 etc.

Answer: The reviewer brings up an important point and as a result we include a benchmarking analysis for 31 gene-pseudogene groups that are involved in cancer. We extracted the benchmark dataset from PMID: 26442270, PMID: 22726445, and PMID: 29240947. PMID:26442270 is a review of well documented pseudogenes and their functions by a well-known researcher Dr. Poliseno. PMID: 22726445 is a Cell article detailing expressed pseudogenes across 13 human cancers and their targets. PMID: 29240947 is a bench science paper about FTH1 regulation by its pseudogenes. This article also describes some of the other pseudogene-gene relationships described by the previous two papers.

We use this benchmarking experiment in place of Figure 4 because it contains much more information. We derived these associations from well-known studies on the subject and found that we can identify 87% of the groups using all databases, 65% using consensus sequences, and identify 3 benchmark gene-pseudogene pairs using consensus sequences that did not appear using BLAST. The examples found by the consensus sequence method but not by BLAST show that the CUDAlign method is useful. Since best practice would have a researcher try multiple databases, a researcher will identify most of the benchmarks. We believe it is also worth noting that we identified these relationships independently of known relationships. As a result, there will inevitably be subtle differences due to the data and methods used during the generation of different flavors of databases.

Benchmarking table
GeneBLASTCUDAlign18CUDAlign54CUDAlign135CUDAlign198PMID
PTENYesNoNoNoNo26442270
TUSCNoNoNoNoNo26442270
INTS6YesNoNoNoNo26442270
OCT4YesYesYesYesYes26442270
HMGA1YesYesYesYesYes26442270
CYP4Z1NoNoNoNoNo26442270
BRAFYesNoNoNoNo26442270
KLK4NoNoNoNoNo22726445
ATP8A2NoYesYesNoNo22726445
CXADRNoYesYesYesYes22726445
CALM2YesYesYesYesYes22726445
TOMM40YesYesYesYesYes22726445
NONOYesYesYesYesYes22726445
PERPNoYesYesYesYes22726445
DUSP8YesYesNoNoNo22726445
YES1YesYesNoNoNo22726445
GJA1YesNoNoNoNo22726445
AURKAYesYesYesYesYes22726445
RHOBNoNoNoNoNo22726445
HMGB1YesYesYesYesYes22726445
EIF4A1YesYesNoNoNo22726445
EIF4HYesYesYesYesYes22726445
SNRP6YesYesYesYesYes22726445
RAB1YesNoNoNoNo22726445
VDAC1YesYesNoNoNo22726445
RCC2YesNoNoNoNo22726445
PTMAYesYesYesYesYes22726445
NDUFA9YesYesYesYesYes22726445
CES7YesNoNoNoNo22726445
EPCAMYesYesYesYesYes22726445
FTH1YesYesYesYesYes29240947
Hits24/3120/3116/3115/3115/31
Total hits27/31

1) Pg 10 highlighted 9 pseudogenes aligned to 15000 gene families and could highlight potential errors in the annotation or if they are collagen-like pseudogenes or

Powered by Editorial Manager® and ProduXion Manager® from Aries Systems Corporation

znf-pseudogenes with repetitive features that align everywhere would be interesting to highlight and give a list of the genes in a table.

Answer: We agree with the reviewer on this point and as a result further elaborate upon the high homology pseudogenes, and described these findings in more detail in paragraph 2-3 of Results. Specifically, we found zinc finger pseudogenes and other domain binding patterns in the highest homology pseudogenes. We found large bodies of evidence that the high homology pseudogenes have either direct or indirect relationships with zinc finger genes.
In Results paragraph 2:
"Another feature of note is that there are some pseudogenes that align to many gene families (e.g., 9 pseudogenes, UBE2Q2P1, RP11-313J2.1, TPTEP1, BMS1P1, CTD-2245F17.3, SCAND2P, GTF2IP7, WHAMMP3, IGLV3-2, have alignment scores above 54 in 15,000 gene families and 571 pseudogenes, see Supplementary Table 2, have alignment scores above 54 in 1,000 gene families)."
In Results paragraph 3:
"Of the 9 highest homology pseudogenes (Supplementary Table 2), one, RP11-313J2.1, is a zinc finger pseudogene and two, CTD-2245F17.3 and SCAND2P, are located in the promoters of zinc finger genes. Four pseudogenes in the 9 highest homology pseudogenes (RP11-313J2.1, CTD-2245F17.3, SCAND2P, and WHAMMP3) also have 92-96% sequence identity with zinc finger genes (ZNF72P, ZNF518A, ZNF37A and ZNF788P/ZNF20 respectively) when BLAST searched against the human genome. Of the 571 highest homology pseudogenes (Supplementary Table 2), we found 27 zinc finger pseudogenes. Using EnrichR59 we identified enrichment in GO Molecular Function GO:0004430 1-phosphatdylinositol 4-kinase activity (Fisher's exact test p-value = 0.001), and enrichment for GO Biological Process GO:0070475 rRNA base methylation (Fisher's exact test p-value = 0.003). In the ARCHS4 database60 324 transcription factors were significantly co-expressed (Benjamini-Hochberg adjusted Fisher's exact test p-value < 0.05) with members of the 571 highest homology pseudogenes. Of those 324 transcription factors, 228 were zinc finger genes. These findings show that the highest homology pseudogenes, like zinc finger genes, likely contain repetitive elements that align to many genomic loci."

2) Fig 3 show the different CUDAlign cutoff and overlap with Pseudogene.org. However there is no detailed explanation why there are over 3500 pseudogenes are not detected by this method of alignment using blast or CDUAlign and is there anything specific about these pseudogenes, are they all 1:1 relationship with parent gene?

Answer: The reviewer identifies an important area, which we have been working on since the initial submission. We have found that a significant portion of these pseudogenes/genes that are in the newer version of the Pseudogenes.org database are not contained in our GENCODEv25 annotation. These missing genes and pseudogenes account for 1030 of the 2458 pseudogene-gene pairs that are in Pseudogenes.org but not in our databases. If these are excluded we recreate 85% of the Pseudogenes.org pseudogene-gene pairs (1:1 relationships). Furthermore this 85% accuracy is similar to our benchmarking accuracy (87%) on genes whose annotation will likely not change drastically between annotation builds. Alternatively, since these genes and pseudogenes were from a different annotation version the sequences themselves could be slightly different causing differences between our database and Pseudogenes.org. These results can be found in the Results section "Direct comparison to pseudogene parents".
In Results paragraph 5:
"Our databases also generate a larger pool of possible interactions. It is worth noting that 391 pseudogenes and 152 genes in the new Pseudogene.org (GENCODEv10) are not present in the GENCODEv25 annotation used in our analysis. These genes and pseudogenes together account for 1030 edges that were used in our comparison. Accounting for these differences in the annotation, we are able to reconstruct 85% of the pseudogene-gene relationships in the new Pseudogene.org database. Since these associations were generated without prior pseudogene-gene relationship information and the annotations have changed slightly since Pseudogenes.org, our methods prove to independently identify known and unknown pseudogene-gene relationships at a high rate."

3) For the use case example, I do not fully understand why the CDUAlign18 was

| | |
|---|---|
| | used for PPPARIL identification in sox15 and not detected in the CDUAlign54 or CDUAlign135. Looking at the sox15 network using CDUAlign135 an alternative pseudogene PIN2 pseudogene can be found. Can the authors explain why this is not also considered as potential regulator and why it does not appear in the TGCA expression panel with the rest of the sox genes ?<br><br>Answer: We thank the reviewer for their insight and have further evaluated the SOX15 network in response. RP11-506B6.5, the pseudogene located next to PIN2, is retained in the more stringent databases (e.g, CUDAlign135) and as such should also be considered. However, RP11-506B6.5 lacks enough annotation from existing literature to make it a promising candidate. The PPPARIL gene has more supporting literature and is a larger more complex pseudogene containing 19 exons opposed to 1 exon in RP11-506B6.5.<br><br>4) Since the usability of the web app is highlighted in the paper, I would recommend a direct link from the Ensembl Identifiers to Ensembl rather than Genecards eg ENST00000428294 does not have a Genecard entry but is classified as a transcribed unprocessed pseudogene by GENCODE/Ensembl.<br><br>Answer: We thank the reviewer for this suggestion and as a result have added this functionality to the website. When a user selects a network node, a tab for GeneCards and a tab for Ensembl appears for the specified gene/pseudogene.<br><br>5) Also the network in the webapp would be easier to navigate if the HGNC identifier was used as default name rather than the ENSG ID (as this should be relatively easy to code) and therefore recommend figure 4 be redrawn as looks extremely hard to interpret.<br><br>Answer: We appreciate these suggestions and have focused on our main application that is available online (https://integrativeomics.shinyapps.io/pseudofun_app/). In this web application HGNC identifiers are used throughout. The old Figure 4 is from another application we developed for research purposes through the Ohio Supercomputer Center. As a result, we moved the old Figure 4 to the supplementary material so that it does not detract from the usability of the main application (which has much more improved user-friendly visualization) freely available online.<br><br>6) Fig 4 should have details of the CDU align cut off used in the legend for the network graphs similar to fig 3<br><br>Answer: We thank the reviewers for their concerns and have added this information to Figure 4, which has been moved to the supplementary material as Supplementary Figure 2. We feel that this figure is of less importance after running the benchmarking experiment, shown in Table 2.<br><br>Minor issues:<br>* Pg12 line 12 "regulation" typo<br><br>* Pg 16 sentence should have "network" inserted before gene on line<br><br>Answer: We appreciate the help from the reviewer for identifying language errors in the manuscript and have made the changes. |
| **Additional Information:** | |
| **Question** | **Response** |
| Are you submitting this manuscript to a special series or article collection? | No |
| **Experimental design and statistics**<br><br>Full details of the experimental design and statistical methods used should be given | Yes |

Powered by Editorial Manager® and ProduXion Manager® from Aries Systems Corporation

| | |
|---|---|
| in the Methods section, as detailed in our Minimum Standards Reporting Checklist. Information essential to interpreting the data presented should be made available in the figure legends.<br><br>Have you included all the information requested in your manuscript? | |
| **Resources**<br><br>A description of all resources used, including antibodies, cell lines, animals and software tools, with enough information to allow them to be uniquely identified, should be included in the Methods section. Authors are strongly encouraged to cite Research Resource Identifiers (RRIDs) for antibodies, model organisms and tools, where possible.<br><br>Have you included the information requested as detailed in our Minimum Standards Reporting Checklist? | Yes |
| **Availability of data and materials**<br><br>All datasets and code on which the conclusions of the paper rely must be either included in your submission or deposited in publicly available repositories (where available and ethically appropriate), referencing such data using a unique identifier in the references and in the “Availability of Data and Materials” section of your manuscript.<br><br>Have you have met the above requirement as detailed in our Minimum Standards Reporting Checklist? | Yes |

Manuscript Click here to access/download;Manuscript;PseudoFuN_Giga_20181212.pdf
Click here to view linked References

# PseudoFuN: Deriving functional potentials of pseudogenes from integrative relationships with genes and miRNAs across 32 cancers

Travis S Johnson[1], Sihong Li[1], Eric Franz[2], Zhi Huang[3,4], Shuyu Dan Li[5], Moray J Campbell[6], Kun Huang[4,7], Yan Zhang[1]*

[1] Department of Biomedical Informatics, College of Medicine, The Ohio State University, Columbus, OH 43210, USA

[2] Ohio Supercomputer Center (OSC), Columbus, OH 43212, USA

[3] Department of Electrical and Computer Engineering, Purdue University, West Lafayette, IN 47907, USA

[4] School of Medicine, Indiana University, Indianapolis, IN 46202, USA

[5] Department of Genetics and Genomic Sciences, Icahn School of Medicine at Mount Sinai, New York, NY 10029, USA

[6] Division of Pharmaceutics and Pharmaceutical Chemistry, College of Pharmacy, The Ohio State University, Columbus, OH 43210, USA

[7] School of Informatics and Computing, Indiana University, Indianapolis, IN 46262, USA

* Correspondence: yan.zhang@osumc.edu

## Abstract

**Background:** Long thought "relics" of evolution, not until recently have pseudogenes been of medical interest regarding regulation in cancer. Often, these regulatory roles are a direct byproduct of their close sequence homology to protein coding genes. Novel pseudogene-gene functional associations can be identified through the integration of biomedical data, such as sequence homology, functional pathways, gene expression, pseudogene expression, and miRNA expression. However, not all of the information has been integrated, and the vast majority of previous pseudogene studies relied on 1:1 pseudogene-parent gene relationships

without leveraging other homologous genes/pseudogenes. **Results:** We produce pseudogene-gene (PGG) families that expand beyond the current 1:1 paradigm. Firstly, we construct expansive PGG databases by i) CUDAlign GPU accelerated local alignment of all pseudogenes to gene families (totaling 1.6 billion individual local alignments and more than 40,000 GPU hours) and ii) BLAST-based assignment of pseudogenes to gene families. Secondly, we create an open-source web application (PseudoFuN) to search for integrative functional relationships of sequence homology, miRNA expression, gene expression, pseudogene expression, and gene ontology. We produce four "flavors" of databases (>462,000,000 pseudogene-gene pairwise alignments and 133,770 PGG families) that can be queried and downloaded using PseudoFuN. These databases are consistent with previous 1:1 pseudogene-gene annotation and also are much more powerful including millions of *de novo* pseudogene-gene associations. For example, we find multiple known (e.g., miR-20a-PTEN-PTENP1) and novel (e.g., miR-375-SOX15- PPP4R1L) miRNA-gene-pseudogene associations in prostate cancer. PseudoFuN provides a "one stop shop" for identifying and visualizing thousands of potential regulatory relationships related to pseudogenes in TCGA cancers. **Conclusions:** Thousands of new pseudogene-gene associations can be explored in the context of miRNA-gene-pseudogene co-expression and differential expression with a simple-to-use online tool by bioinformaticians and oncologists alike.

**Keywords**: Pseudogenes, database, functional prediction, gene regulation, network analysis, high performance computing, graphics processing unit, competing endogenous RNA

## Background

Pseudogenes were previously considered unimportant relics of evolution that played an unclear role in biological processes[1]. However, more pseudogenes have been discovered to be involved in gene regulation[2-4]. These regulatory relationships between pseudogenes and genes have increasingly been explored, such as the transcriptional regulation of PTEN by pseudogene PTENP1 in several cancer conditions[5]. PTEN acts as a tumor suppressor gene, which is underexpressed in gastric cancer. However by overexpressing PTENP1 in gastric cancer, both PTEN underexpression and cell proliferation are mitigated via the regulatory relationship between PTEN and PTENP1[6]. Relationships between these pseudogenes and their parent genes have been found to play critical roles indicating functional potentials of these pseudogenes[7,8]. This point can most clearly be seen in the importance of sequence homology between pseudogenes and coding genes plays in competing endogenous RNA (ceRNA) networks[9,10]. In ceRNA networks the pseudogenes act as decoy targets for the miRNAs targeting a protein-coding gene. In short, researchers have made huge strides in understanding pseudogenes from genomic variation to functional potentials[11,12], and from “deciphering” the mechanism of ceRNA networks[13] to experimental validation[14].

With this progress, there has been renewed interest in pseudogenes, especially in relation to cancer[15]. This interest has even uncovered biomarkers in human cancer including but not limited to SUMO1P3 upregulation as a diagnostic biomarker in gastric cancer and OCT4-pg4 expression as a prognostic biomarker in hepatocellular carcinoma (HCC)[16-18]. Pseudogene expression has been used to stratify tumor subtypes in 7 distinct cancer types[19]. However, due to the close sequence homology between pseudogenes and their parent genes, identifying the expression profile unique to a pseudogene or highly homologous gene can be challenging. Efforts have been made to address these technical challenges in estimating pseudogene expression using modified alignment and quantification techniques[20]. Perhaps more intriguingly

is that pseudogenes can be somatically acquired in cancer development effectively "representing a new class of mutations" that can be either activating or inactivating mutations which function as an "on/off switch"[21,22]. Specific pseudogenes have been implicated in specific cancers. For example, FTH1 regulates tumorigenesis in prostate cancer[23], TP73-AS1 regulates proliferation in esophageal squamous cell carcinoma[24], and pseudogenes NKAPP1, MSTO2P and RPLP0P2 are associated with poor prognosis in lung adenocarcinoma[25].

For these reasons, having a complete understanding of these pseudogene-gene relationships is important. While studying these relationships, a common conception is to only consider the pseudogenes in relation to their parent genes with highest homology[7-9,26]. There have also been pioneer studies probing pseudogene functions through aligning them to parent proteins (corresponding to the parent genes) and then to parent protein domains[7,27,28].

The conventional idea of single parent genes may not be comprehensive enough to model the complex phylogenetic relationships involving multiple genes and pseudogenes in a homolog family. While pseudogenes diverged from their parent genes distantly in the past, only the daughter protein-coding genes other than the original parent gene may now exist. The result is that aligning to the true phylogenetic parent gene itself may not be possible. For this reason, we advocate the use of homologous gene families rather than single parent genes to compare against pseudogenes. By viewing the homologies as a weighted network instead of a single scalar value we believe that new relationships can be uncovered.

We build the pseudogene-gene (PGG) family databases using two methods: i) CUDAlign[29] based-local alignment of all pseudogenes to gene families (totaling 1.6 billion individual local alignments and more than 40,000 GPU hours). By aligning all pseudogenes to all gene families (CUDAlign), we can study underlying sequence homology and more easily set cutoffs to assign

pseudogenes to gene families. ii) BLAST [30]-based assignment of pseudogenes to gene families. This provides a fast heuristic search option. BLAST derivative methods have been commonly used to find parent genes in previous pseudogene studies[31,32]. Using these two methods we show that these pseudogenes are usually assigned to the gene family of their parent genes but are often not exclusively so. Besides, most pseudogenes can be categorized into processed pseudogenes and unprocessed pseudogenes depending on whether they came from retrotranscription of mRNAs[11,33,34]. We take these differences into account using both of our methods (CUDAlign and BLAST).

Furthermore, we make these data publicly downloadable from GitHub[35]. We also create an R Shiny web application called PseudoFuN[36] that supports querying the PGG databases, interactive visualization and functional analysis of the PGG networks, and visualization of pseudogene-gene co-expression and miRNA binding (including binding prediction with Miranda[37], PicTar[38], and TargetScan[39]) using The Cancer Genome Atlas and GTEx (Genotype-Tissue Expression) project derived public data[20,40,41]. Besides, we provide another interactive web application hosted by the Ohio Supercomputer Center[42] (OSC), which supports querying novel sequences against any of our PGG databases and visualization of the resulting PGG networks.

The PGG databases can be used to study pseudogene-gene-miRNA co-expression indicative of ceRNA networks across the entire Cancer Genome Atlas. With these diverse tools provided by PseudoFuN, it is possible to generate hypotheses regarding i) the regulatory roles of pseudogenes across tumor and normal tissue, ii) pseudogene-gene relationships through *de novo* reassignment of pseudogenes to gene families and iii) functional annotation of pseudogenes. We expect these databases and tools to have more use in cancer studies.

## Methods

### *Construction of Pseudogene-Gene (PGG) Database*

To generate these gene families, we use two methods: i) CUDAlign-based local alignment of pseudogenes against consensus sequences representing gene families, and ii) BLAST-based search of pseudogene sequences against all gene sequences (Figure 1). These two approaches can be thought of as heuristic but different processes. The local sequence alignment approach is heuristic in that only two gene sequences are used from each gene family to reduce the search space. These sequences are the most similar and representative sequences to all the other gene sequences in the family. The BLAST-based approach is heuristic in that not all sequences are fully aligned during the process due to the seed-and-extend steps of BLAST[43]. The result is that not every relationship between pseudogene and gene family is recorded which is an advantage in runtime but a disadvantage in studying underlying sequence homology.

*i) CUDAlign-based local alignment of gene families*

Gene homolog families were generated using the Ensembl biomart gene homolog database[44,45]. The pairs of homologous genes were separated into connected components using python networkx package[46]. These connected component sub-graphs are considered gene families in this study. To reduce the number of alignments that needed to be performed, we selected consensus genes from each family that would be used to represent the entire family.

The consensus sequences were selected by aligning every member of the gene family to every other member using local alignment with CUDAlign[29]. The two members of the family with the largest sum alignment scores across all other family members were selected as the consensus sequences to increase the number of candidate sequences. If only one member existed in the family, then that member was the consensus sequence. Using the list of these consensus

sequences we then aligned every consensus sequence to every pseudogene in the human genome GRCh38 annotated by GENCODE Release 25 (GENCODEv25)[47].

Specifically the pseudogenes are split up into processed, unprocessed and other (unclear whether processed or unprocessed), based on their mechanisms of formation[48]. We performed different alignment procedures for processed and unprocessed pseudogenes respectively. The processed pseudogenes were aligned to all consensus gene transcripts with the highest local alignment score recorded. The unprocessed pseudogenes were aligned to the full genomic sequences of each of the consensus genes with the highest local alignment score recorded. Theoretically unprocessed pseudogenes can align to both exonic and intronic regions of DNA, while processed pseudogene can only align to exonic regions. In our previous database we did not perform this two-procedure strategy in part to reduce the runtime of the problem[49]. These changes make the database much more complete and biologically relevant. The other pseudogenes were aligned to both the transcripts and the genomic sequence recording the highest score.

These scores, one for each combination of pseudogene to gene family, were stored for further analysis. Pseudogenes were assigned to families using a cutoff score (i.e., percentiles of the alignment scores per PGG alignment matrix) and a maximum number of assignments (i.e., the top four alignments above a cutoff). If greater than top four alignments were used, the PGG families were too large to calculate the pairwise alignment matrix. The resulting sets of pseudogenes and genes are called pseudogene-gene (PGG) families. This method was used to allow a pseudogene to be assigned multiple families as well as prevent pseudogenes from being assigned families if their alignment score was low. We used the 99$^{th}$ percentile cutoff (corresponding alignment score 54), 99.9$^{th}$ percentile cutoff (135), and the 99.99$^{th}$ percentile cutoff (198) to generate three resultant databases named CUDAlign54, CUDAlign135, and

CUDAlign198 respectively. A fourth database that is less stringent, CUDAlign18, is also included in the web applications using a 97.5$^{th}$ percentile cutoff (18). All these flavors of databases are available for search in our web applications.

*ii) BLAST-based generation of PGG families*

In contrast to the local alignment of every combination of pseudogene to gene family, PGG families were also created by assigning the pseudogenes to the family containing its closest BLAST search match. This approach was used to contrast with the CUDAlign method, which uses up to the top 4 matches. The pseudogenes were separated into processed, unprocessed and other. Then, all genes in the GENCODE Release 25 annotation were used to generate genomic, transcript, and combined BLAST databases (BlastDB). The processed pseudogenes would be BLAST searched against transcript BlastDB, unprocessed against the genomic sequence BlastDB, and the rest pseudogenes were BLAST searched against the combined genomic/transcript BlastDB. The pseudogene was assigned to the gene family containing the best match from the BLAST search.

***Comparison between PGG families and pseudogene-parent gene pairs***

We also conduct a comparison to the Pseudogene.org resource[50]. In this comparison, we consider pseudogenes and parent gene pairs from pseudogene.org psiDr[31] database (old)[51] and on GENCODE Release 10 from pseudogene.org psiCube[11] database (new)[52]. From our databases, we consider every combination of pseudogene to gene within a PGG family as a pair (for example, a family with 3 genes and 2 pseudogenes would have $C_2^3 = 6$ pairs). Since we have multiple flavors of PGG databases including the BLAST-based version and the CUDAlign-based versions, we compare the intersections between two Pseudogene.org versions and our

BLAST/CUDAlign-based versions. We show the intersections of pseudogene-gene pairs in Venn Diagrams.

### *Development of PseudoFuN web applications*

Aside from generating different flavors of the PGG databases, we assemble them into an online R Shiny application called PseudoFuN[36] which supports gene and pseudogene symbol queries against out PGG databases, generates dynamic networks, produces Gene Ontology[53] (GO) tables and additional functional analysis features (Table 1). The functionalities, such as calculating the gene co-expression for any resultant PGG network in any of the TCGA[54] cancers types, are important for ceRNA network hypothesis generation in human cancers. For more information, please visit the PseudoFuN website and follow the README and tutorial.

Additionally we create another web application hosted by the Ohio Supercomputer Center (OSC) OnDemand[55] platform. This application has multiple functionalities including the query of Ensembl gene ID or a novel sequence against one selected flavor of our databases. For each of these features we provide a simple-to-use interface that allows users to select which database to query, allows download of the query hits, and allows users to interactively explore the PGG family networks including GO information.

### *Use cases in multiple cancers*

Furthermore three use cases are provided to show the potential utility of PseudoFuN to researchers and oncologists looking for functional relationships between pseudogenes, genes, and miRNAs. Use Case I validates known pseudogene-gene functional relationships. Use Case II identifies high confidence novel miRNA-pseudogene-gene relationships. Use Case III is primarily focused on agreement with a validation study. We focused on pseudogenes/genes that were differentially expressed in low RARG/low TACC1/high miR-96 compared to the reverse in

prostate cancer cell lines and also differentially expressed in our PGG networks in TCGA prostate cancer samples.

## Results

*Local alignment of gene families*

We performed 1.6 billion local alignments between all pseudogenes and all gene family consensus sequences. The process required over 40,000 GPU hours on the Oakley cluster at the OSC. The highest scores for each gene family and pseudogene were stored in a 17,273x26,754 matrix of pseudogene-to-gene-family alignment scores (~462 million elements). From this matrix, we are able to explore global pseudogene-gene family homology relationships and assign pseudogenes to one or more gene families with high sequence homology.

As one might expect, the number of pseudogenes with high alignments (defined as above a percentile threshold) to many gene families is relatively low. It can be seen that the majority of pseudogenes will align to one gene family in the CUDAlign databases (Figure 2). We evaluate alignment of pseudogenes to genes using the Smith-Watermann local pairwise alignment score[56] between a pseudogene and a gene. These scores indicate the highest score possible for two sequences based on their specific dynamic programing matrix which is solved by the Smith-Watermann algorithm. The cutoffs we use, 18, 54, 135, and 198, indicate the 97.50$^{th}$, 99.0$^{th}$, 99.90$^{th}$ and 99.99$^{th}$ percentiles of alignment scores in our alignment matrix between all pseudogenes and consensus sequences. Another feature of note is that there are some pseudogenes that align to many gene families (e.g., 9 pseudogenes, UBE2Q2P1, RP11-313J2.1, TPTEP1, BMS1P1, CTD-2245F17.3, SCAND2P, GTF2IP7, WHAMMP3, IGLV3-2, have alignment scores above 54 in 15,000 gene families and 571 pseudogenes, see Supplementary Table 2, have alignment scores above 54 in 1,000 gene families).

In contrast to previous belief in single gene-pseudogene homology, some pseudogenes are related to many genes. It is worth considering that these high homology pseudogenes (e.g., FTLP10 with 3,006 gene family pairwise alignments over a 54 threshold) may have a role in regulating major biological processes[57] and disease[58]. Of the 9 highest homology pseudogenes (Supplementary Table 2), one, RP11-313J2.1, is a zinc finger pseudogene and two, CTD-2245F17.3 and SCAND2P, are located in the promoters of zinc finger genes. Four pseudogenes in the 9 highest homology pseudogenes (RP11-313J2.1, CTD-2245F17.3, SCAND2P, and WHAMMP3) also have 92-96% sequence identity with zinc finger genes (ZNF72P, ZNF518A, ZNF37A and ZNF788P/ZNF20 respectively) when BLAST searched against the human genome. Of the 571 highest homology pseudogenes (Supplementary Table 2), we found 27 zinc finger pseudogenes. Using EnrichR[59] we identified enrichment in GO Molecular Function GO:0004430 1-phosphatdylinositol 4-kinase activity (Fisher's exact test p-value = 0.001), and enrichment for GO Biological Process GO:0070475 rRNA base methylation (Fisher's exact test p-value = 0.003). In the ARCHS4 database[60] 324 transcription factors were significantly co-expressed (Benjamini-Hochberg adjusted Fisher's exact test p-value < 0.05) with members of the 571 highest homology pseudogenes. Of those 324 transcription factors, 228 were zinc finger genes. These findings show that the highest homology pseudogenes, like zinc finger genes, likely contain repetitive elements that align to many genomic loci.

*BLAST generation of PGG families*

The BLAST generated database was larger than the CUDAlign generated databases with 68,578 total connections. This database was also much simpler to compute with since it was not an exhaustive search. These conclusions make it a simple method to quickly estimate the pseudogene-to-gene relationships.

*Direct comparison to pseudogene parents*

We compare our databases to the previous pseudogene-parent gene databases retrieved from Pseudogene.org resources (Figure 3). It shows that our methods reconstruct most of the pseudogene-parent-gene relationships identified by Pseudogene.org. The overall consistency of our databases (BLAST and CUDAlign) with both Pseudogenes.org databases (new and old) was 75% (i.e., all our databases combined). Individually, the BLAST-based database contained 61% of the Pseudogene.org relationships (both new and old) and the CUDAlign 54 cutoff contained 60% of the Pseudogene.org relationships (both new and old). Our databases also generate a larger pool of possible interactions. It is worth noting that 391 pseudogenes and 152 genes in the new Pseudogene.org (GENCODE Release 10) are not present in the GENCODE Release 25 annotation used in our analysis. These genes and pseudogenes together account for 1030 edges that were used in our comparison. Accounting for these differences in the annotation, we are able to reconstruct 85% of the pseudogene-gene relationships in the new Pseudogene.org database. Since these associations were generated without prior pseudogene-gene relationship information and the annotations have changed slightly since Pseudogenes.org, our methods prove to independently identify known and unknown pseudogene-gene relationships at a high rate.

*Development of a pseudogene query tool*

The R Shiny application is a comprehensive hypothesis generating tool that is freely available on the internet[36]. This tool provides a wide array of functionality that a researcher can access quickly and download results as the raw data for more in-depth analysis. These features are outlined in detail in Table 1.

*Use Cases: Assisting functional study of ceRNA networks in cancer*

To illustrate the utility of our databases and tools we present three use cases.

Use Case I: To validate known pseudogene-gene relationships, we first identified 31 benchmark pseudogene-gene relationships from three studies[15,16,23] and query our databases. These studies represent prominent regulatory pseudogenes in cancers by established laboratories. We query a gene/pseudogene name one at a time and PseudoFuN will return the top PGG network(s) that contain the query (Table 2). In general, we found that our databases together were able to identify 87% of the benchmarking cases (Table 2) and the CUDAlign versions were able to identify 65% of the benchmarking cases. Perhaps most importantly, three of the cases identified by CUDAlign (ATP8A2, CXADR, PERP) were not identified by the more traditional BLAST approach (Table 2) showing that consensus sequence alignment can identify some overlooked relationships. Next, individual benchmark cases were evaluated in more detail (Supplementary Figure 2).

PTENP1 is a processed pseudogene homologous to PTEN, a tumor suppressor gene. PTENP1 is selectively lost in cancer and may regulate PTEN expression as a miRNA decoy target[5,6]. We have observed differential co-expression patterns of PGG families in tumor vs. normal for PTENP1 network in multiple cancers including breast cancer (Supplementary Figure 3B,C). We identified known miRNAs (has-miR-93 targets PTEN in breast cancer[61]) targeting PTEN PGG network nodes providing insights into ceRNA regulation (Supplementary Figure 3D). These insights are important since some pseudogenes competitively bind to miRNAs thus regulate gene expression. We also identify hsa-miR-103a-3p, known to regulate PTEN in endometrial[62] and colorectal cancers[63], in breast cancer (Supplementary Figure 3D). The miRNA hsa-miR-20a, known to regulate PTEN by the ceRNA mechanism in prostate cancer[64], was also identified in breast cancer. The ceRNA network regulatory relationship is governed by effect modulation of miRNA on gene expression by pseudogene expression (Supplementary Figure 1A,C,E). This leads to a correlation between pseudogene (miRNA decoy targets) and gene (miRNA targets) expression (Supplementary Figure 1D). That means both these pseudogenes

and homologous genes competitively bind to miRNAs. KRAS-KRASP1 regulatory network was also identified by our database (Supplementary Figure 2). KRAS and KRASP1 are known to be involved in ceRNA network regulation[5,10,64]. PseudoFuN query of KRAS identified co-expression patterns in prostate cancer consistent with ceRNA network regulation by hsa-miR-145, a known modulator of KRAS in prostate cancer[65]. The FTH1 query also resulted in the identification of pseudogenes (FTH1P2, FTH1P8, FTH1P11, FTH1P16) that regulate FTH1 in prostate cancer[23] as well novel miRNAs that may be involved in ceRNA network regulation of FTH1 in prostate cancer. GBP1 is an IFN-α induced transcript that is involved in immune response in prostate cancer[66]. The GBP1 involved PGG network also contained the pseudogene GBP1P1 which may have a ceRNA regulatory role in breast cancer[67] and in some neurodegenerative diseases[68].

Use Case II: We wanted to identify possible gene-miRNA relationships of interest within our database. We chose to study these relationships with respect to miR-96, a known cancer regulator microRNA in prostate cancer[69]. Through differential expression analysis between tumors in the TCGA-PRAD cohort with lower expression of RARG and TACC1 (also a miR-96 target) and high expression miR-96 (low RARG/low TACC1/high miR-96), compared to the reverse, we previously identified altered SOX15 gene expression is significantly associated with worse disease-free survival. We visualized expression patterns of SOX15 PGG families, and corresponding miRNA associations. miR-96 is included as a validation.

Interestingly we identified the pseudogene PPP4R1L as a potential member of a SOX15 ceRNA network (Figure 4A). PPP4R1L and SOX15 are both significantly differentially expressed between tumor and normal controls (Bonferroni corrected p-value = $3.42\times10^{-7}$, $2.01\times10^{-14}$ respectively, Figure 4E). PPP4R1L and SOX15 are significantly co-expressed (Pearson correlation coefficient (PCC)=0.51, p-value<$2.2\times10^{-16}$) in tumor tissue but much less correlated in normal controls in prostate cancer (PCC=0.24, p-value=0.09, Figure 4B,C). Positively

correlated expression is an assumption when determining ceRNA network relationships[70] (Supplementary Figure 1). Both SOX15 and PPP4R1L are likely regulated by hsa-miR-375 based on the TCGA prostate cancer dataset. hsa-miR-375 is associated with docetaxel resistance in prostate cancer[71,72] and PPP4R1L knock-down in HeLa cells induces taxol resistance[73]. These findings are intriguing since taxol and docetaxel are closely related chemical compounds. PPP4R1L is also located in a region associated with high mutation rates in cancer cell lines[73] which could be indicative of mutational "on/off switches" in pseudogene regulation.

Use Case III: We were most interested in the deferentially expressed (DE) genes (and related pseudogenes) that both appeared in our PGG database and were contained in networks with genes differentially expressed in low RARG/low TACC1/high miR-96 compared to vice versa. We searched the DE genes in our PGG database, and identified the top networks with enriched number of DE genes. As a result, parent genes HTR7, CNN2, MSN and TAGLN2 are differentially expressed; they generate pseudogenes, which are specifically expressed in prostate cancer samples[16]. These four parent genes are also detected in our 5 top PGG families involving miR-96 regulated (direct or indirect) DE genes. We identified HTR7P1 pseudogene in the same PGG family as HTR7 gene, which is potentially regulated by hsa-miR-607 and has-miR-3654 in the TCGA prostate cancer dataset (Supplementary Figure 4). 11 CNN2 pseudogenes (CNN2P1-CCN2P4, CNN2P6-CNN2P12) were identified in the CNN2 PGG family along with TAGLN2 and TAGLN2P1. TAGLN2P1 is differentially expressed between the tumor and normal samples in the prostate dataset (Supplementary Figure 5, Bonferroni corrected p-value = 6.23×10-4). MSN and MSNP1 were in the same PGG family and hsa-miR-96 potentially regulates MSN in the TCGA prostate cancer dataset (Supplementary Figure 5). In addition, although our DE genes were detected from prostate cancer, we further compared them with DE pseudogenes identified in four other cancer types and we observed interesting results (see Supplementary Materials - *Potential regulatory roles in cancer*).

## Discussion

We identify 133,770 PGG families that have significant potential to reveal important information about regulatory pseudogene-gene relationships in health and disease. Within these families we identify both new and existing regulatory networks that contain pseudogenes such as PTENP1, KRAS1P, FTH1P8/11/16, and GBP1P1 (Figure 4). Since all genes and all pseudogenes are included in our database there are thousands of opportunities to identify new regulatory relationships. These thousands of opportunities can be easily stratified using gene name, pseudogene name and cancer type. Our PseudoFuN web application makes it a simple and intuitive process to query pseudogenes (or genes) to identify which gene families they may be regulating as well as the functions that are attributed to the members of the network. We also have an application hosted by the OSC that allows the querying of novel sequences against our database.

From these networks, we can also identify possible relationships of differentially expressed pseudogenes in various cancers. For instance, both PPP4R1L pseudogene and SOX15 are differentially expressed in prostate cancer and associated with hsa-miR-375. These types of relationships should be further evaluated along with more complex regulation with multiple miRNAs, pseudogenes, and genes. It is experimentally shown that SOX15 is regulated by hsa-miR-96[69]. It may be important to include hsa-miR-96 in the hsa-miR-375-SOX15-PPP4R1L potential ceRNA network. Aside from PGG family specific differential pseudogene expression, the PseudoFuN application allows for comprehensive differential pseudogene expression (DPgE) analysis in any of the TCGA cancer datasets.

The use of this database also has utility in integrative analysis where the databases can be used as a mask for other data modalities. Some examples would be using the nodes (genes

and pseudogenes) in each of the PGG families as groups in gene expression experiments. Similarly, these groups could be used for feature reduction when visualizing data. We hope researchers can use these relationships we have identified to reduce large numbers of candidate associations down to numbers that can be easily validated and generate new candidates when querying novel sequences. For instance, miRNA-gene pairs filtered through the sets of PGG families would identify high priority ceRNA candidates.

## Conclusions

We generate multiple large databases of pseudogene gene family relationships and the tools to study them for use by biomedical researchers. These databases are more comprehensive than previous pseudogene-gene databases by including many more homology relationships in PGG families, thus more powerful for experiment validation and knowledge discovery. These databases are useful in identifying pseudogene-gene regulatory relationships in 32 cancer types and show high similarity with known pseudogene-gene relationships. Aside from the known relationships we identify many unknown relationships. Furthermore, these databases and associated analyses can be easily accessed online or through the OSC OnDemand platform, allowing for novel hypotheses to be assessed quickly by biomedical researchers. We find evidence of both known regulatory pseudogene-gene relationships and novel hypothesized relationships that we plan to validate. PseudoFuN is a comprehensive, dynamic tool that allows any bioinformatician or oncologist to find novel regulatory pseudogenes within their cancer or gene network of interest.

## Availability of Supporting Data

We have made the PGG family data publicly downloadable from GitHub[35]. We also created an R Shiny web application called PseudoFuN[36] that supports querying the PGG databases,

interactive visualization and functional analysis of the PGG networks, and visualization of pseudogene-gene co-expression and miRNA binding. Apache License 2.0 is associated with PseudoFuN (R Shiny web application). Besides, we provide another interactive web application hosted on Ohio Supercomputer Center (OSC) OnDemand, which supports querying novel sequences against any of our PGG databases and visualization of the resulting PGG networks.

## Additional Files

There is an additional Supplementary Materials file containing additional information on the data and additional analyses. It includes the following figures and tables:

**Supplementary Figure 1. Example of ceRNA network regulation of gene expression.** A) A graphical view of how pseudogene expression can regulate gene expression. B) A cellular view of ceRNA network regulation. C) Equations used to model the correlation between gene and pseudogene expression in a ceRNA network. D) The distribution of the gene-pseudogene correlations based on the models in C. E) The effect that pseudogene expression has on the miRNA induced change in gene expression.

**Supplementary Figure 2. PseudoFuN online output for PTEN PGG family.** A) Interactive graph visualization of the PTEN PGG network. B) TCGA prostate co-expression matrix for PTEN PGG family genes and pseudogenes across normal samples. C) TCGA prostate co-expression matrix for PTEN PGG family genes and pseudogenes across tumor samples. D) Negatively correlated miRNAs for all members of the PTEN PGG family. E) Differential gene and pseudogene expression for tumor and normal samples for each member of the PTEN PGG family in the prostate cancer TCGA dataset.

**Supplementary Figure 3. PseudoFuN online output for HTR7 PGG family.** A) Interactive graph visualization of the HTR7 PGG network. B) TCGA breast cancer co-expression matrix for HTR7 PGG family genes and pseudogenes across normal samples. C) TCGA breast cancer co-

expression matrix for HTR7 PGG family genes and pseudogenes across tumor samples. D) Negatively correlated miRNAs for all members of the HTR7 PGG family in breast cancer. E) Differential gene and pseudogene expression for tumor and normal samples for each member of the HTR7 PGG family in the breast cancer TCGA dataset.

**Supplementary Figure 4. PseudoFuN online output for CNN2/TAGLN2 PGG family.** A) Interactive graph visualization of the CNN2/TAGLN2 PGG network. B) TCGA prostate co-expression matrix for CNN2/TAGLN2 PGG family genes and pseudogenes across normal samples. C) TCGA prostate co-expression matrix for CNN2/TAGLN2 PGG family genes and pseudogenes across tumor samples. D) Negatively correlated miRNAs for all members of the CNN2/TAGLN2 PGG family. E) Differential gene and pseudogene expression for tumor and normal samples for each member of the CNN2/TAGLN2 PGG family in the prostate cancer TCGA dataset.

**Supplementary Figure 5. PseudoFuN online output for MSN PGG family.** A) Interactive graph visualization of the MSN PGG network. B) TCGA prostate co-expression matrix for MSN PGG family genes and pseudogenes across normal samples. C) TCGA prostate co-expression matrix for MSN PGG family genes and pseudogenes across tumor samples. D) Negatively correlated miRNAs for all members of the MSN PGG family. E) Differential gene and pseudogene expression for tumor and normal samples for each member of the MSN PGG family in the prostate cancer TCGA dataset.

**Supplementary Figure 6. The PGG families in our network with the most DE genes after miR-96 treatment.** The line weights indicate the sequence homology between members of the PGG family. Red nodes indicate miR-96 targets. Yellow nodes with names indicate other genes contained in the PGG family. Yellow nodes without names are pseudogenes contained within the network.

**Supplementary Figure 7. The user interface of the OSC OnDemand web application.** A) is the main query page where a user can search either sequences or Ensembl gene IDs. B) is a representative output of one of the gene searches. This includes an interactive network and the GO information.

**Supplementary Figure 8. GBP1P1 DE in TCGA prostate cancer** (information retrieved from Han et al.)**.**

**Supplementary Table 1. DE parent gene/pseudogenes potentially regulated by miR-96 in prostate cancer vs. TCGA derived DE pseudogenes.**

## Abbreviations

PseudoFuN: Pseudogene Functional Networks

PGG: Pseudogene-Gene (i.e., PGG families)

TCGA: The Cancer Genome Atlas

ceRNA: Competing Endogenous RiboNucleic Acid

HCC: HepatoCellular Carcinoma

BLAST: Basic Local Alignment and Search Tool

OSC: Ohio Supercomputer Center

GO: Gene Ontology

DE: Differential Expression

DGE: Differential Gene Expression

DPgE: Differential Pseudogene Expression


## *Acknowledgments*

This work is partially supported by NIH-NLM MIDAS Training Fellowship (4T15LM011270-05) awarded to Travis Johnson and The Ohio State University Startup Funds to Yan Zhang. The

authors also thank the Ohio Supercomputer Center (OSC) for providing computing resources.

### *Author contributions*

TSJ, SL, ZH and YZ performed data analyses. TSJ, EF and ZH developed the web applications. YZ and TSJ conceived and initiated this project. YZ and KH supervised the project. MJC provided experimental data. All authors contributed to biological interpretation. TSJ, YZ, MJC and SDL wrote the manuscript. All authors read and approved the manuscript.

### *Ethics, consent and permissions*

Not applicable.

### *Consent to publish*

Not applicable.

### *Competing interests*

The authors declare that they have no competing interests.

## Figure Captions

**Figure 1. Workflow for both CUDAlign and BLAST databases.** Left side PGG families are produced using the BLAST matches. Right side PGG families are produced using the pseudogene-gene-family alignment matrix with percentile cutoffs using CUDAlign.

**Figure 2. The number pseudogenes that align to gene families.** The x-axis is the number of gene families, which have an alignment score above a specified cutoff (the different colored lines). The y-axis is the number of pseudogenes with an alignment score higher than the cutoff to the number of gene families on the x-axis. The inset grey box is a closer view of the low range gene family numbers (1-10) to show more granular patterns.

**Figure 3. Comparison of database members.** The top 6 plots are comparisons between the CUDAlign databases using different cutoffs, the BLAST database, and the Pseudogene.org parent genes. The bottom row shows intra-database comparisons, left: Pseudogene.org, middle: CUDAlign databased of different alignment score cutoffs, right: relative size of all databases.

**Figure 4. PseudoFuN online output for SOX15 PGG family.** A) Interactive graph visualization of the SOX15 PGG network. B) TCGA prostate co-expression matrix for SOX15 PGG family genes and pseudogenes across normal samples. C) TCGA prostate co-expression matrix for SOX15 PGG family genes and pseudogenes across tumor samples. D) Negatively correlated miRNAs for all members of the SOX15 PGG family. E) Differential gene and pseudogene expression for tumor and normal samples for each member of the SOX15 PGG family in the prostate cancer TCGA dataset.

## Tables

**Table 1 Summary of features that are freely available at the PseudoFuN website.**

| PseudoFuN features | Additional description |
| --- | --- |

| | |
|---|---|
| Interactive visualization of PGG family networks including the query pseudogene/gene | Users can query any single gene or pseudogene symbol, e.g., PTENP1. Nodes are colored by sub-clusters within the network. |
| Functional enrichment analysis of PGG family | Functional enrichment can be conducted on the genes within the PGG family on Biological Process, Molecular Function or Cellular Components annotations. The GO functional enrichment is calculated with:<br>1. Fisher's exact test[74]<br>2. Kolmogorov-Smirnov (KS) Classic[75]<br>3. Kolmogorov-Smirnov (KS) Elim[75] |
| Genomic loci mapping of PGG family | The genes in the PGG family can be mapped back to the genome using a circus plot to identify potential loci of interest. |
| Data download for all of the figures | Users can also download results including:<br>1. the differential pseudogene expression (DPgE) table for all pseudogenes in the selected cancer<br>2. the gene and pseudogene expression<br>3. miRNA correlation table |
| Links to other gene databases for more information | By directly clicking the node in the network, users can open the GeneCards and Ensembl websites[44,76] for detailed gene information. |
| Gene/pseudogene co-expression analysis across the entire TCGA | Once a PGG family has been identified the gene/pseudogene co-expression matrix is calculated across one of the 32 available TCGA cancer types. |
| Tumor vs. normal differential expression of genes/pseudogenes across all TCGA cancer types | The gene/pseudogene differential expression is calculated for all members of the selected PGG family. There is also an option to run differential expression on a specified cancer for all pseudogenes which can be viewed or downloaded as a table. |
| Predicted miRNA targets involved in the PGG families across all TCGA cancer types | The miRNA targets involved in the selected cancer and PGG family are displayed to show which miRNAs could regulate the PGG family members. This is by using the miRNA correlation tables from the TCGA. |
| Differential Pseudogene Expression (DPgE) Analysis | Differential pseudogene expression is calculated for each of the pseudogenes in TCGA cancers using dreamBase |

| | |
|---|---|
| | expression information[20]. The online tool allows for manipulation and download of the table. |

**Table 2. Benchmarking analysis of PseudoFuN databases. Genes indicate the gene with which the pseudogenes are associated in the literature.** BLAST and CUDAlign columns indicate the specific databases. PMID indicates the literature from which the gene-psuedogene relationship was derived. Yellow highlighting indicates gene-pseudogene relationships found using BLAST but not CUDAlign. Green highlighting indicate indicates gene-pseudogene relationships found by CUDAlign but not by BLAST. Orange highlighting indicate where neither type of database identified the benchmark gene-pseudogene relationship. Benchmark totals are included at the bottom of the table.

| Gene | BlastDB | CUDAlign18 | CUDAlign54 | CUDAlign135 | CUDAlign198 | PMID |
|---|---|---|---|---|---|---|
| PTEN | Yes | No | No | No | No | 26442270 |
| TUSC | No | No | No | No | No | 26442270 |
| INTS6 | Yes | No | No | No | No | 26442270 |
| OCT4 | Yes | Yes | Yes | Yes | Yes | 26442270 |
| HMGA1 | Yes | Yes | Yes | Yes | Yes | 26442270 |
| CYP4Z1 | No | No | No | No | No | 26442270 |
| BRAF | Yes | No | No | No | No | 26442270 |
| KLK4 | No | No | No | No | No | 22726445 |
| ATP8A2 | No | Yes | Yes | No | No | 22726445 |
| CXADR | No | Yes | Yes | Yes | Yes | 22726445 |
| CALM2 | Yes | Yes | Yes | Yes | Yes | 22726445 |
| TOMM40 | Yes | Yes | Yes | Yes | Yes | 22726445 |
| NONO | Yes | Yes | Yes | Yes | Yes | 22726445 |
| PERP | No | Yes | Yes | Yes | Yes | 22726445 |
| DUSP8 | Yes | Yes | No | No | No | 22726445 |
| YES1 | Yes | Yes | No | No | No | 22726445 |
| GJA1 | Yes | No | No | No | No | 22726445 |
| AURKA | Yes | Yes | Yes | Yes | Yes | 22726445 |
| RHOB | No | No | No | No | No | 22726445 |
| HMGB1 | Yes | Yes | Yes | Yes | Yes | 22726445 |
| EIF4A1 | Yes | Yes | No | No | No | 22726445 |
| EIF4H | Yes | Yes | Yes | Yes | Yes | 22726445 |
| SNRP6 | Yes | Yes | Yes | Yes | Yes | 22726445 |
| RAB1 | Yes | No | No | No | No | 22726445 |
| VDAC1 | Yes | Yes | No | No | No | 22726445 |
| RCC2 | Yes | No | No | No | No | 22726445 |
| PTMA | Yes | Yes | Yes | Yes | Yes | 22726445 |
| NDUFA9 | Yes | Yes | Yes | Yes | Yes | 22726445 |
| CES7 | Yes | No | No | No | No | 22726445 |
| EPCAM | Yes | Yes | Yes | Yes | Yes | 22726445 |
| FTH1 | Yes | Yes | Yes | Yes | Yes | 29240947 |
| Hits | 24/31 | 20/31 | 16/31 | 15/31 | 15/31 | |
| Total hits | 27/31 | | | | | |

## References


1. Vanin EF: Processed pseudogenes: characteristics and evolution. Annu Rev Genet 19:253-72, 1985
2. Mighell AJ, Smith NR, Robinson PA, et al: Vertebrate pseudogenes. FEBS Lett 468:109-14, 2000
3. Pink RC, Wicks K, Caley DP, et al: Pseudogenes: pseudo-functional or key regulators in health and disease? RNA 17:792-8, 2011
4. Chan JJ, Tay Y: Noncoding RNA:RNA Regulatory Networks in Cancer. Int J Mol Sci 19, 2018
5. Poliseno L, Salmena L, Zhang J, et al: A coding-independent function of gene and pseudogene mRNAs regulates tumour biology. Nature 465:1033-8, 2010
6. Zhang R, Guo Y, Ma Z, et al: Long non-coding RNA PTENP1 functions as a ceRNA to modulate PTEN level by decoying miR-106b and miR-93 in gastric cancer. Oncotarget 8:26079-26089, 2017
7. Lam HY, Khurana E, Fang G, et al: Pseudofam: the pseudogene families database. Nucleic Acids Res 37:D738-43, 2009
8. Zheng D, Gerstein MB: A computational approach for identifying pseudogenes in the ENCODE regions. Genome Biol 7 Suppl 1:S13 1-10, 2006
9. An Y, Furber KL, Ji S: Pseudogenes regulate parental gene expression via ceRNA network. J Cell Mol Med 21:185-192, 2017
10. Poliseno L, Pandolfi PP: PTEN ceRNA networks in human cancer. Methods 77-78:41-50, 2015
11. Sisu C, Pei B, Leng J, et al: Comparative analysis of pseudogenes across three phyla. Proc Natl Acad Sci U S A 111:13361-6, 2014
12. Zhang Y, Li S, Abyzov A, et al: Landscape and variation of novel retroduplications in 26 human populations. PLoS Comput Biol 13:e1005567, 2017
13. Cesana M, Daley GQ: Deciphering the rules of ceRNA networks. Proc Natl Acad Sci U S A 110:7112-3, 2013
14. Chiu HS, Martinez MR, Bansal M, et al: High-throughput validation of ceRNA regulatory networks. BMC Genomics 18:418, 2017
15. Poliseno L, Marranci A, Pandolfi PP: Pseudogenes in Human Cancer. Front Med (Lausanne) 2:68, 2015
16. Kalyana-Sundaram S, Kumar-Sinha C, Shankar S, et al: Expressed pseudogenes in the transcriptional landscape of human cancers. Cell 149:1622-34, 2012
17. Mei D, Song H, Wang K, et al: Up-regulation of SUMO1 pseudogene 3 (SUMO1P3) in gastric cancer and its clinical association. Med Oncol 30:709, 2013
18. Wang L, Guo ZY, Zhang R, et al: Pseudogene OCT4-pg4 functions as a natural micro RNA sponge to regulate OCT4 expression by competing for miR-145 in hepatocellular carcinoma. Carcinogenesis 34:1773-81, 2013
19. Han L, Yuan Y, Zheng S, et al: The Pan-Cancer analysis of pseudogene expression reveals biologically and clinically relevant tumour subtypes. Nat Commun 5:3963, 2014
20. Zheng LL, Zhou KR, Liu S, et al: dreamBase: DNA modification, RNA regulation and protein binding of expressed pseudogenes in human health and disease. Nucleic Acids Res 46:D85-D91, 2018

21. Cooke SL, Shlien A, Marshall J, et al: Processed pseudogenes acquired somatically during cancer development. Nat Commun 5:3644, 2014

22. Shukla R, Upton KR, Munoz-Lopez M, et al: Endogenous retrotransposition activates oncogenic pathways in hepatocellular carcinoma. Cell 153:101-11, 2013

23. Chan JJ, Kwok ZH, Chew XH, et al: A FTH1 gene:pseudogene:microRNA network regulates tumorigenesis in prostate cancer. Nucleic Acids Res 46:1998-2011, 2018

24. Zang W, Wang T, Wang Y, et al: Knockdown of long non-coding RNA TP73-AS1 inhibits cell proliferation and induces apoptosis in esophageal squamous cell carcinoma. Oncotarget 7:19960-74, 2016

25. Wei Y, Chang Z, Wu C, et al: Identification of potential cancer-related pseudogenes in lung adenocarcinoma based on ceRNA hypothesis. Oncotarget 8:59036-59047, 2017

26. Milligan MJ, Lipovich L: Pseudogene-derived lncRNAs: emerging regulators of gene expression. Front Genet 5:476, 2014

27. Bateman A, Birney E, Durbin R, et al: The Pfam protein families database. Nucleic Acids Res 28:263-6, 2000

28. Finn RD, Mistry J, Schuster-Bockler B, et al: Pfam: clans, web tools and services. Nucleic Acids Res 34:D247-51, 2006

29. Chirag Jain SK: Fine-grained GPU parallelization of pairwise local sequence alignment. Presented at the 21st International Conference on High Performance Computing (HiPC, 2014

30. Soroceanu L, Matlaf L, Khan S, et al: Cytomegalovirus Immediate-Early Proteins Promote Stemness Properties in Glioblastoma. Cancer Res 75:3065-76, 2015

31. Pei B, Sisu C, Frankish A, et al: The GENCODE pseudogene resource. Genome Biology 13:R51, 2012

32. Zhang Z, Carriero N, Zheng D, et al: PseudoPipe: an automated pseudogene identification pipeline. Bioinformatics 22:1437-1439, 2006

33. Lynch M, Conery JS: The evolutionary fate and consequences of duplicate genes. Science 290:1151-5, 2000

34. Baertsch R, Diekhans M, Kent WJ, et al: Retrocopy contributions to the evolution of the human genome. BMC Genomics 9:466, 2008

35. Zhang Y: PseudoFuN GitHub. https://github.com/yanzhanglab/PseudoFuN_app, 2018

36. Johnson TS, Li S, Franz E, et al: PseudoFuN. https://integrativeomics.shinyapps.io/pseudofun_app/, 2018

37. Miranda KC, Huynh T, Tay Y, et al: A pattern-based method for the identification of MicroRNA binding sites and their corresponding heteroduplexes. Cell 126:1203-17, 2006

38. Krek A, Grun D, Poy MN, et al: Combinatorial microRNA target predictions. Nat Genet 37:495-500, 2005

39. Agarwal V, Bell GW, Nam JW, et al: Predicting effective microRNA target sites in mammalian mRNAs. Elife 4, 2015

40. Grossman RL, Heath AP, Ferretti V, et al: Toward a Shared Vision for Cancer Genomic Data. N Engl J Med 375:1109-12, 2016

41. Carithers LJ, Moore HM: The Genotype-Tissue Expression (GTEx) Project. Biopreserv Biobank 13:307-8, 2015

42. Center OS: Ohio Supercomputer Center. Columbus OH, Ohio Supercomputer Center, 1987
43. Altschul SF, Gish W, Miller W, et al: Basic local alignment search tool. J Mol Biol 215:403-10, 1990
44. Zerbino DR, Achuthan P, Akanni W, et al: Ensembl 2018. Nucleic Acids Res 46:D754-D761, 2018
45. Ensembl: Ensembl Biomart. ensembl.org/biomart/martview, 2018
46. Hagberg A, Swart P, S Chult D: Exploring network structure, dynamics, and function using NetworkX, Los Alamos National Lab.(LANL), Los Alamos, NM (United States), 2008
47. Harrow J, Frankish A, Gonzalez JM, et al: GENCODE: the reference human genome annotation for The ENCODE Project. Genome Res 22:1760-74, 2012
48. Echols N, Harrison P, Balasubramanian S, et al: Comprehensive analysis of amino acid and nucleotide composition in eukaryotic genomes, comparing genes and pseudogenes. Nucleic Acids Res 30:2515-23, 2002
49. Johnson TS, Li S, Kho JR, et al: Network analysis of pseudogene-gene relationships: from pseudogene evolution to their functional potentials. Pac Symp Biocomput 23:536-547, 2018
50. Karro JE, Yan Y, Zheng D, et al: Pseudogene.org: a comprehensive database and comparison platform for pseudogene annotation. Nucleic Acids Res 35:D55-60, 2007
51. pseudogenes.org: psiDr. pseudogenes.org/psidr/similarity.dat
52. pseudogenes.org: psiCube. http://pseudogene.org/psicube/
53. Ashburner M, Ball CA, Blake JA, et al: Gene Ontology: tool for the unification of biology. Nature genetics 25:25, 2000
54. Cancer Genome Atlas Research N, Weinstein JN, Collisson EA, et al: The Cancer Genome Atlas Pan-Cancer analysis project. Nat Genet 45:1113-20, 2013
55. Hudak D, Johnson D, Chalker A, et al: Open OnDemand: A web-based client portal for HPC centers.
56. Smith TF, Waterman MS: Identification of common molecular subsequences. J Mol Biol 147:195-7, 1981
57. Carmona U, Li L, Zhang L, et al: Ferritin light-chain subunits: key elements for the electron transfer across the protein cage. Chem Commun (Camb) 50:15358-61, 2014
58. Wu T, Li Y, Liu B, et al: Expression of Ferritin Light Chain (FTL) Is Elevated in Glioblastoma, and FTL Silencing Inhibits Glioblastoma Cell Proliferation via the GADD45/JNK Pathway. PLoS ONE 11:e0149361, 2016
59. Kuleshov MV, Jones MR, Rouillard AD, et al: Enrichr: a comprehensive gene set enrichment analysis web server 2016 update. Nucleic Acids Res 44:W90-7, 2016
60. Lachmann A, Torre D, Keenan AB, et al: Massive mining of publicly available RNA-seq data from human and mouse. Nat Commun 9:1366, 2018
61. Li N, Miao Y, Shan Y, et al: MiR-106b and miR-93 regulate cell progression by suppression of PTEN via PI3K/Akt pathway in breast cancer. Cell Death Dis 8:e2796, 2017
62. Guo C, Song WQ, Sun P, et al: LncRNA-GAS5 induces PTEN expression through inhibiting miR-103 in endometrial cancer cells. J Biomed Sci 22:100, 2015
63. Geng L, Sun B, Gao B, et al: MicroRNA-103 promotes colorectal cancer by targeting tumor suppressor DICER and PTEN. Int J Mol Sci 15:8458-72, 2014

64. Yang C, Wu D, Gao L, et al: Competing endogenous RNA networks in human cancer: hypothesis, validation, and perspectives. Oncotarget 7:13479-90, 2016

65. Cui SY, Wang R, Chen LB: MicroRNA-145: a potent tumour suppressor that regulates multiple cellular pathways. J Cell Mol Med 18:1913-26, 2014

66. Persano L, Moserle L, Esposito G, et al: Interferon-alpha counteracts the angiogenic switch and reduces tumor cell proliferation in a spontaneous model of prostatic cancer. Carcinogenesis 30:851-60, 2009

67. Welch JD, Baran-Gale J, Perou CM, et al: Pseudogenes transcribed in breast invasive carcinoma show subtype-specific expression and ceRNA potential. BMC Genomics 16:113, 2015

68. Costa V, Esposito R, Aprile M, et al: Non-coding RNA and pseudogenes in neurodegenerative diseases: "The (un)Usual Suspects". Front Genet 3:231, 2012

69. Long MD, Singh PK, Russell JR, et al: The miR-96 and RARgamma signaling axis governs androgen signaling and prostate cancer progression. Oncogene, 2018

70. Xu J, Feng L, Han Z, et al: Extensive ceRNA-ceRNA interaction networks mediated by miRNAs regulate development in multiple rhesus tissues. Nucleic Acids Res 44:9438-9451, 2016

71. Costa-Pinheiro P, Ramalho-Carvalho J, Vieira FQ, et al: MicroRNA-375 plays a dual role in prostate carcinogenesis. Clin Epigenetics 7:42, 2015

72. Wang Y, Lieberman R, Pan J, et al: miR-375 induces docetaxel resistance in prostate cancer by targeting SEC23A and YAP1. Mol Cancer 15:70, 2016

73. MacKeigan JP, Murphy LO, Blenis J: Sensitized RNAi screen of human kinases and phosphatases identifies new regulators of apoptosis and chemoresistance. Nat Cell Biol 7:591-600, 2005

74. F.R.S. RAF: Tests of significance in harmonic analysis. Proceedings of the Royal Society of London. Series A 125:54, 1929

75. Alexa A RJ: Gene set enrichment analysis with topGO. http://www.bioconductor.org, Bioconductor, 2009

76. Stelzer G, Rosen N, Plaschkes I, et al: The GeneCards Suite: From Gene Data Mining to Disease Genome Sequence Analyses. Curr Protoc Bioinformatics 54:1 30 1-1 30 33, 2016

Figure 1

Click here to access/download;Figure;Figure1_pressrdy.pdf

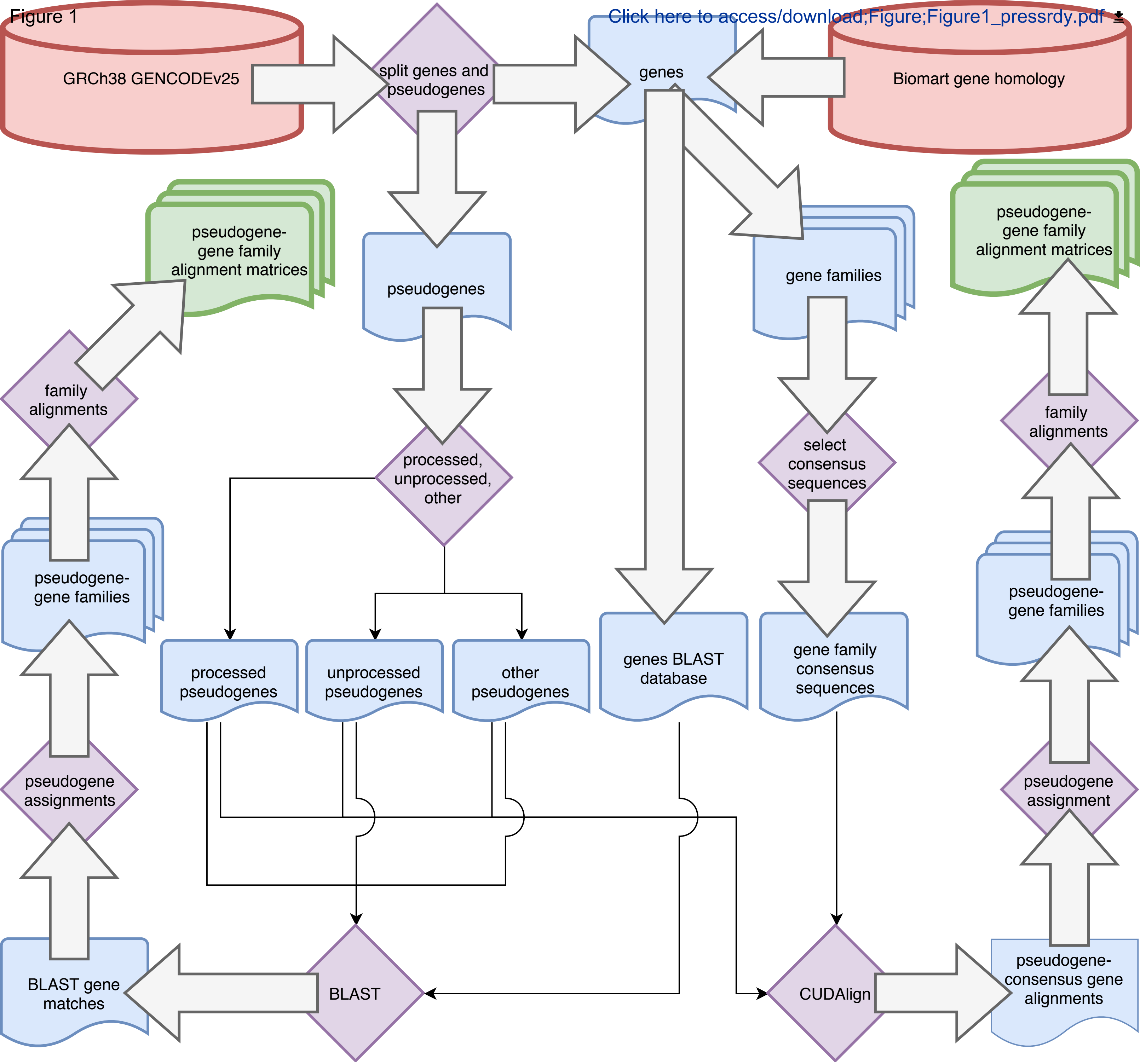

Figure 2

Click here to access/download;Figure;Figure2_pressrdy.pdf

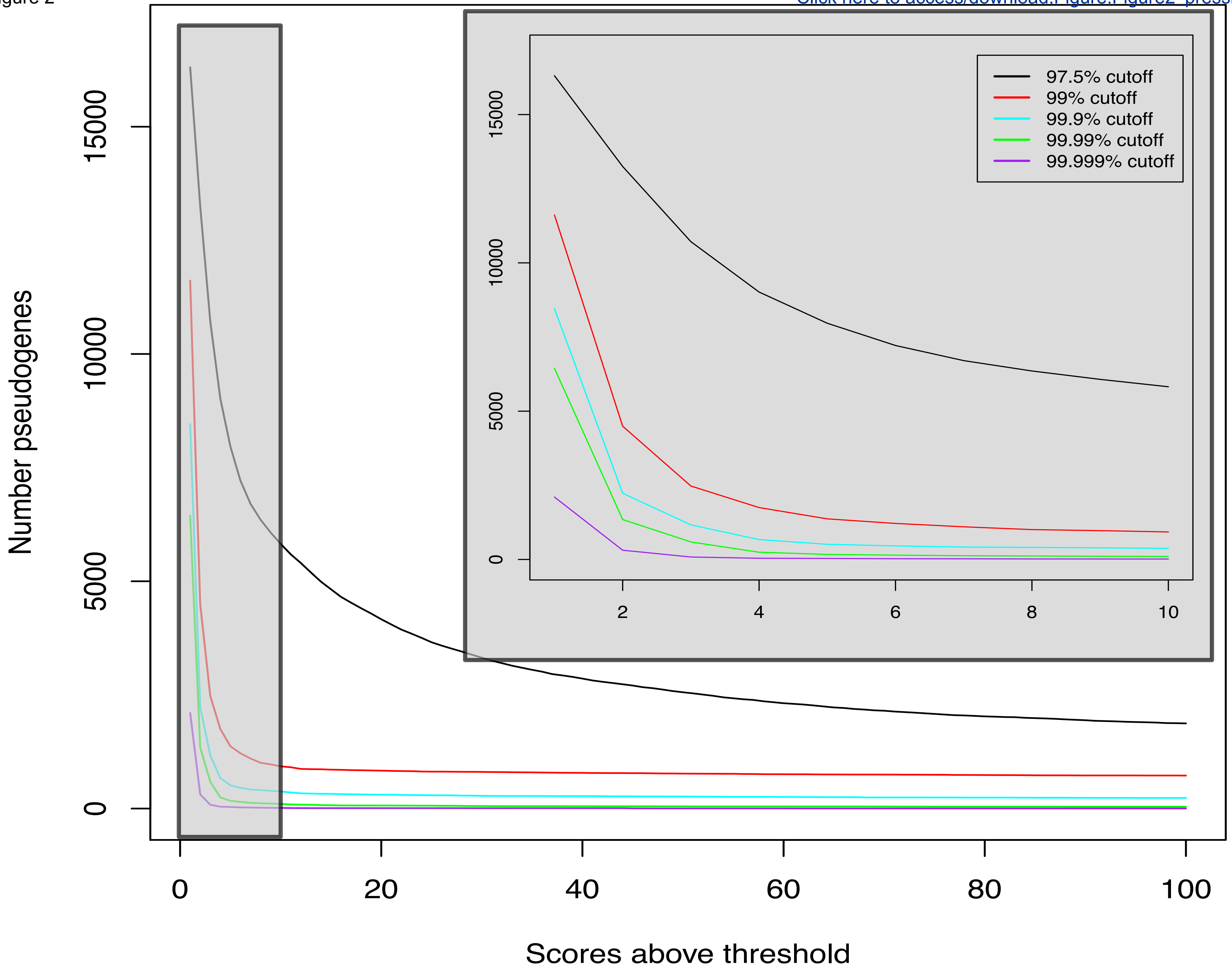

Figure 3

Click here to access/download;Figure;Figure3_pressrdy.pdf

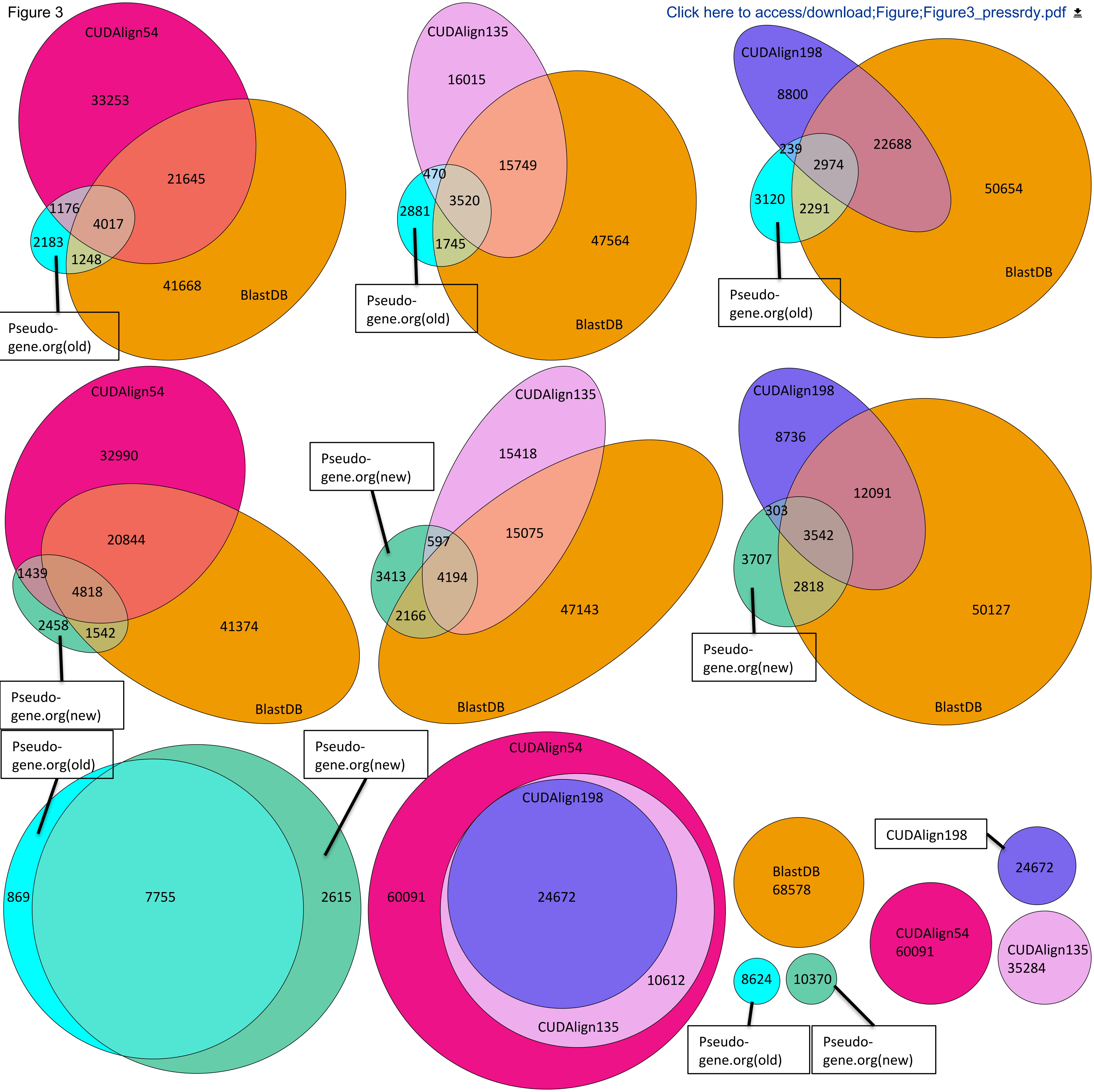

Figure 4

Click here to access/download;Figure;Figure4_pressrdy.pdf

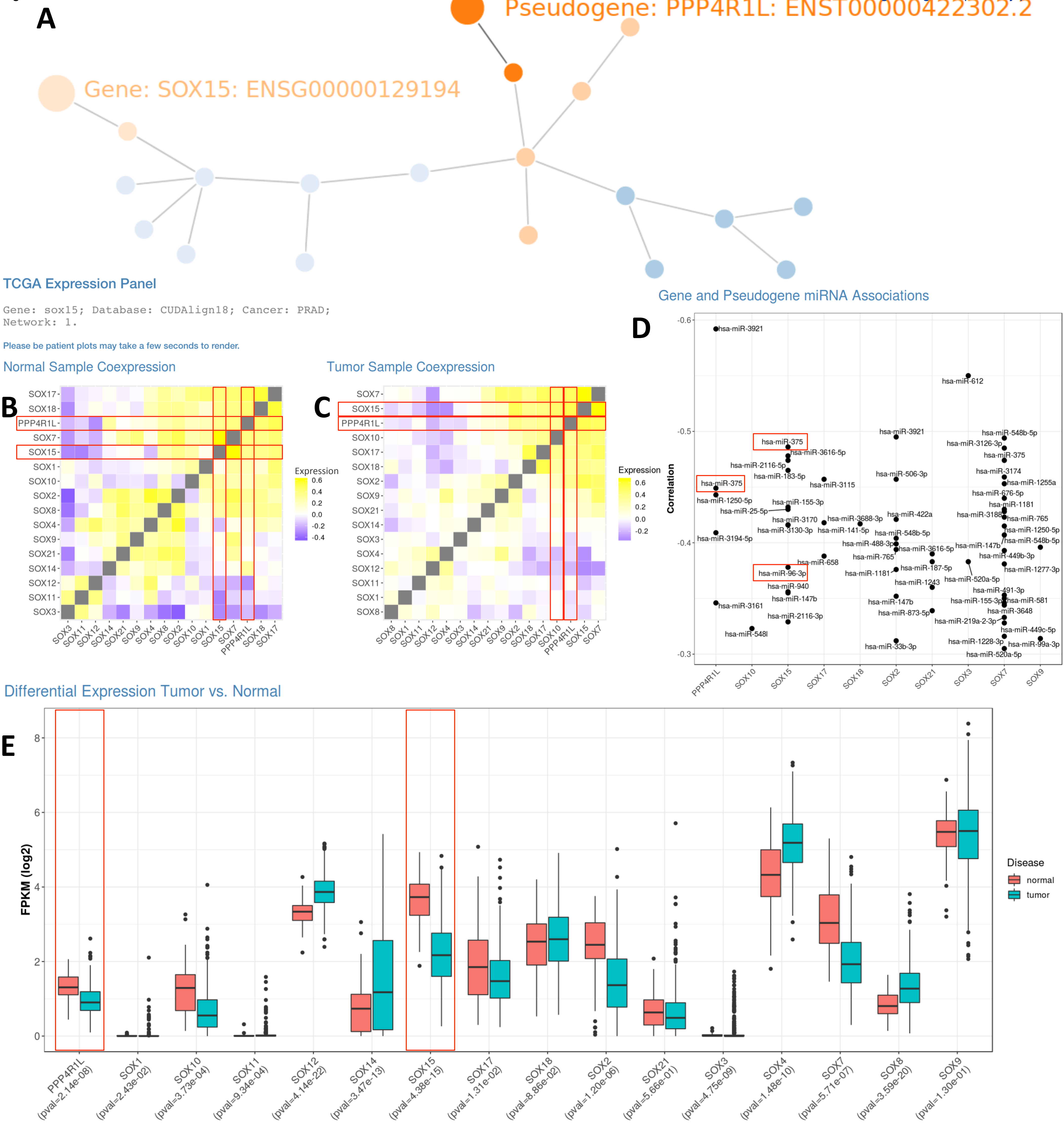

Click here to access/download
**Supplementary Material**
PseudoFuN_suppl_Giga_20181212.pdf

Personal Cover Click here to access/download;Personal Cover;YanZhang_CoverLetter_20181212_signed.pdf

THE OHIO STATE UNIVERSITY

Yan Zhang, Ph.D.
Assistant Professor
Department of Biomedical Informatics
College of Medicine
The Ohio State University
310-B Lincoln Tower, 1800 Cannon Drive
Columbus, OH 43210
Phone: (614) 688-9643
Email: Yan.Zhang@osumc.edu

December 12, 2018

Dear Editor,

We would like to submit our revision of the manuscript "PseudoFuN: Deriving functional potentials of pseudogenes from integrative relationships with genes and miRNAs across 32 cancers". We have thoroughly revised our manuscript and addressed all the points raised by the reviewers.

We thank you and the reviewers for all the great suggestions that have helped us make the project better. And we hope that this improved version will meet the journal's publication requirement. We look forward to your feedback.

Sincerely,

Yan Zhang

Yan Zhang, Ph.D.
Assistant Professor
Department of Biomedical Informatics
The Ohio State University

Response Letter
Click here to access/download;Personal Cover;Giga_reviews_20181212.pdf

# Response Letter

**We thank the reviewers for their insightful comments and believe that after addressing each comment the manuscript is stronger. Please see the reviewers' comments and our responses below to see specifically how all of the concerns were addressed. We have highlighted all our answers in red color. We also highlight in red color all the changes in the main text.**

Reviewer #1: The role of pseudogenes in the modulation of gene regulation is a burgeoning field that is ideally placed to benefit from integrative approaches that utilise "big data" that is currently available. A user friendly tool such as PseudoFun is therefore of use as a possible discovery mechanism for new relationships. Not having used PsuedoFun at this stage, it is difficult to fully evaluate its performance, though the approach described appears useful and the presentation of new relationships such as that suggested between PPP4RiL, SOX15 and miR-375 highlight a potential to identify new avenues for further investigation. I have only minor suggestions for improvement in presentation.

1) In Figure 5 (and much of the supplementary figures presented in a similar fashion), is the miRNA associated directly targeting the gene/pseudogene. Visually, only a correlative expression relationship is indicated.

**We appreciate the reviewer's feedback. In Figure 5 (currently Figure 4), the miRNAs associated with gene/pseudogene were determined by not only expression correlation but also miRNA target prediction databases: Miranda, PicTar and TargetScan. We downloaded the predictions from http://gdac.broadinstitute.org. According to the reviewer's comment, we have improved the miRNA section on the TCGA Expression panel of the website. The website now allows the user to select how many algorithms predict regulation of the gene/pseudogene by the miRNA. This value is used as a threshold for the displayed miRNAs. The default is 0 meaning that the miRNA and gene/pseudogene are significantly negatively correlated indicative of possible regulation. The value can be changed from 0-3 indicating the number of algorithms (Miranda, PicTar, and TargetScan) predict regulation of gene/pseudogene by the specified miRNA.**

2) Figure 4 does little to add clarity. If the goal is to highlight regulatory relationships, the ENSTxxx labelling does not lend for easy interpretation and the miRNAs are not shown. If the intended purpose is to illustrate a style at which data is outputted, perhaps this is better served by a user friendly series of screenshots illustrating a beginning to end data query - result flow?

**We agree with the reviewer that containing only ENSTxxx labelling does not facilitate illustration. We use easy-to-interpret gene names and links to other gene databases (e.g. GeneCards, Ensembl) to improve the usability of our PseudoFuN website (https://integrativeomics.shinyapps.io/pseudofun_app/), and we have improved the visualization according the reviewers' suggestions. The old Figure 4 is not from our public PseudoFuN version, instead it is from our supercomputer version located on the Ohio Supercomputer Center clusters and is meant mainly for research purposes. Since it is not exactly the version we mainly presented in the main text, which has much more user-friendly interface with interpretable gene names, we moved the old Figure 4 to the supplementary materials so that it does not detract from the usability of the main application freely available online.**

3) In Figure 5 and some supplementary figures, co-expression visually is not well represented by the colour scheme. ie: the tumour relationship between PPP4R1L and SOX15. The stats support this, the visual representation less so. Perhaps blanking out the 1:1 same gene : same gene diagonal would allow re-setting of the colour scheme to better represent co-expression?

**This insightful comment by the reviewer caused us to rethink our visualization. We changed the same gene/pseudogene correlation (1.0) along the diagonal line in the heatmap to NaN so that the**

**visualization ignores those values. This allows the range of color for the other correlations to be more diverse and more informative to the users.**

4) In paragraph 2 of the results, I was unclear what the "alignment score above 54" means... What degree of alignment is this? I found understanding this to be hard to gauge. Relating to this, could the authors comment more extensively on their findings of tremendous levels of alignment for some pseudogenes?

**We agree with the reviewer that we should more fully explain the alignment scores in the manuscript and as a result explain in more detail what the alignment scores represent in paragraph 2 of the results: "We evaluate alignment of pseudogenes to genes using the Smith-Watermann local pairwise alignment score[56] between a pseudogene and a gene. These scores indicate the highest score possible for two sequences based on their specific dynamic programing matrix which is solved by the Smith-Watermann algorithm. The cutoffs we use, 18, 54, 135, and 198, indicate the 97.50$^{th}$, 99.0$^{th}$, 99.90$^{th}$ and 99.99$^{th}$ percentiles of alignment scores in our alignment matrix between all pseudogenes and consensus sequences."**

**We have also performed more in-depth analysis on the high homology pseudogenes and described these findings in more detail in paragraph 2-3 of Results. Specifically, we found zinc finger pseudogenes and other domain binding patterns in the highest homology pseudogenes. We found large bodies of evidence that the high homology pseudogenes have either direct or indirect relationships with zinc finger genes.**
**In Results paragraph 2:**
**"Another feature of note is that there are some pseudogenes that align to many gene families (e.g., 9 pseudogenes, UBE2Q2P1, RP11-313J2.1, TPTEP1, BMS1P1, CTD-2245F17.3, SCAND2P, GTF2IP7, WHAMMP3, IGLV3-2, have alignment scores above 54 in 15,000 gene families and 571 pseudogenes, see Supplementary Table 2, have alignment scores above 54 in 1,000 gene families)."**
**In Results paragraph 3:**
**"Of the 9 highest homology pseudogenes (Supplementary Table 2), one, RP11-313J2.1, is a zinc finger pseudogene and two, CTD-2245F17.3 and SCAND2P, are located in the promoters of zinc finger genes. Four pseudogenes in the 9 highest homology pseudogenes (RP11-313J2.1, CTD-2245F17.3, SCAND2P, and WHAMMP3) also have 92-96% sequence identity with zinc finger genes (ZNF72P, ZNF518A, ZNF37A and ZNF788P/ZNF20 respectively) when BLAST searched against the human genome. Of the 571 highest homology pseudogenes (Supplementary Table 2), we found 27 zinc finger pseudogenes. Using EnrichR[59] we identified enrichment in GO Molecular Function GO:0004430 1-phosphatdylinositol 4-kinase activity (Fisher's exact test p-value = 0.001), and enrichment for GO Biological Process GO:0070475 rRNA base methylation (Fisher's exact test p-value = 0.003). In the ARCHS4 database[60] 324 transcription factors were significantly co-expressed (Benjamini-Hochberg adjusted Fisher's exact test p-value < 0.05) with members of the 571 highest homology pseudogenes. Of those 324 transcription factors, 228 were zinc finger genes. These findings show that the highest homology pseudogenes, like zinc finger genes, likely contain repetitive elements that align to many genomic loci."**

Reviewer #2: The authors have presented an overview of their new analysis and data resource to identify novel pseudogene-gene network interactions that could lead to new hypothesis around their role in regulation of cancer using TCGA cancer expression data and miRNA expression. The unique element of this analysis is using a consensus sequence representing gene families and examining the local alignment of pseudogenes against this consensus to identify new potential interactions.

The major criticism of the paper is that a thorough benchmarking evaluation of their different alignment cutoffs has not been clearly presented, to guide the user when interpreting the network data and deciding which pseudogene appearing in the different networks is worth looking into more depth or reject as being a false positive result. This probably could be done with their validated use cases example taken for the literature such as PTEN /PTENP1 etc.

**The reviewer brings up an important point and as a result we include a benchmarking analysis for 31 gene-pseudogene groups that are involved in cancer. We extracted the benchmark dataset from PMID: 26442270, PMID: 22726445, and PMID: 29240947. PMID:26442270 is a review of well documented pseudogenes and their functions by a well-known researcher Dr. Poliseno. PMID: 22726445 is a Cell article detailing expressed pseudogenes across 13 human cancers and their targets. PMID: 29240947 is a bench science paper about FTH1 regulation by its pseudogenes. This article also describes some of the other pseudogene-gene relationships described by the previous two papers.**

**We use this benchmarking experiment in place of Figure 4 because it contains much more information. We derived these associations from well-known studies on the subject and found that we can identify 87% of the groups using all databases, 65% using consensus sequences, and identify 3 benchmark gene-pseudogene pairs using consensus sequences that did not appear using BLAST. The examples found by the consensus sequence method but not by BLAST show that the CUDAlign method is useful. Since best practice would have a researcher try multiple databases, a researcher will identify most of the benchmarks. We believe it is also worth noting that we identified these relationships independently of known relationships. As a result, there will inevitably be subtle differences due to the data and methods used during the generation of different flavors of databases.**

**Benchmarking table**

| Gene | BLAST | CUDAlign18 | CUDAlign54 | CUDAlign135 | CUDAlign198 | PMID |
|---|---|---|---|---|---|---|
| PTEN | Yes | No | No | No | No | 26442270 |
| TUSC | No | No | No | No | No | 26442270 |
| INTS6 | Yes | No | No | No | No | 26442270 |
| OCT4 | Yes | Yes | Yes | Yes | Yes | 26442270 |
| HMGA1 | Yes | Yes | Yes | Yes | Yes | 26442270 |
| CYP4Z1 | No | No | No | No | No | 26442270 |
| BRAF | Yes | No | No | No | No | 26442270 |
| KLK4 | No | No | No | No | No | 22726445 |
| ATP8A2 | No | Yes | Yes | No | No | 22726445 |
| CXADR | No | Yes | Yes | Yes | Yes | 22726445 |
| CALM2 | Yes | Yes | Yes | Yes | Yes | 22726445 |
| TOMM40 | Yes | Yes | Yes | Yes | Yes | 22726445 |
| NONO | Yes | Yes | Yes | Yes | Yes | 22726445 |
| PERP | No | Yes | Yes | Yes | Yes | 22726445 |
| DUSP8 | Yes | Yes | No | No | No | 22726445 |
| YES1 | Yes | Yes | No | No | No | 22726445 |
| GJA1 | Yes | No | No | No | No | 22726445 |
| AURKA | Yes | Yes | Yes | Yes | Yes | 22726445 |
| RHOB | No | No | No | No | No | 22726445 |
| HMGB1 | Yes | Yes | Yes | Yes | Yes | 22726445 |
| EIF4A1 | Yes | Yes | No | No | No | 22726445 |
| EIF4H | Yes | Yes | Yes | Yes | Yes | 22726445 |
| SNRP6 | Yes | Yes | Yes | Yes | Yes | 22726445 |
| RAB1 | Yes | No | No | No | No | 22726445 |
| VDAC1 | Yes | Yes | No | No | No | 22726445 |
| RCC2 | Yes | No | No | No | No | 22726445 |
| PTMA | Yes | Yes | Yes | Yes | Yes | 22726445 |
| NDUFA9 | Yes | Yes | Yes | Yes | Yes | 22726445 |
| CES7 | Yes | No | No | No | No | 22726445 |
| EPCAM | Yes | Yes | Yes | Yes | Yes | 22726445 |
| FTH1 | Yes | Yes | Yes | Yes | Yes | 29240947 |

| | | | | | | |
|---|---|---|---|---|---|---|
| Hits | 24/31 | 20/31 | 16/31 | 15/31 | 15/31 | |
| Total hits | 27/31 | | | | | |

1) Pg 10 highlighted 9 pseudogenes aligned to 15000 gene families and could highlight potential errors in the annotation or if they are collagen-like pseudogenes or znf-pseudogenes with repetitive features that align everywhere would be interesting to highlight and give a list of the genes in a table.

**We agree with the reviewer on this point and as a result further elaborate upon the high homology pseudogenes, and described these findings in more detail in paragraph 2-3 of Results. Specifically, we found zinc finger pseudogenes and other domain binding patterns in the highest homology pseudogenes. We found large bodies of evidence that the high homology pseudogenes have either direct or indirect relationships with zinc finger genes.**

**In Results paragraph 2:**

**"Another feature of note is that there are some pseudogenes that align to many gene families (e.g., 9 pseudogenes, UBE2Q2P1, RP11-313J2.1, TPTEP1, BMS1P1, CTD-2245F17.3, SCAND2P, GTF2IP7, WHAMMP3, IGLV3-2, have alignment scores above 54 in 15,000 gene families and 571 pseudogenes, see Supplementary Table 2, have alignment scores above 54 in 1,000 gene families)."**

**In Results paragraph 3:**

**"Of the 9 highest homology pseudogenes (Supplementary Table 2), one, RP11-313J2.1, is a zinc finger pseudogene and two, CTD-2245F17.3 and SCAND2P, are located in the promoters of zinc finger genes. Four pseudogenes in the 9 highest homology pseudogenes (RP11-313J2.1, CTD-2245F17.3, SCAND2P, and WHAMMP3) also have 92-96% sequence identity with zinc finger genes (ZNF72P, ZNF518A, ZNF37A and ZNF788P/ZNF20 respectively) when BLAST searched against the human genome. Of the 571 highest homology pseudogenes (Supplementary Table 2), we found 27 zinc finger pseudogenes. Using EnrichR[59] we identified enrichment in GO Molecular Function GO:0004430 1-phosphatdylinositol 4-kinase activity (Fisher's exact test p-value = 0.001), and enrichment for GO Biological Process GO:0070475 rRNA base methylation (Fisher's exact test p-value = 0.003). In the ARCHS4 database[60] 324 transcription factors were significantly co-expressed (Benjamini-Hochberg adjusted Fisher's exact test p-value < 0.05) with members of the 571 highest homology pseudogenes. Of those 324 transcription factors, 228 were zinc finger genes. These findings show that the highest homology pseudogenes, like zinc finger genes, likely contain repetitive elements that align to many genomic loci."**

2) Fig 3 show the different CUDAlign cutoff and overlap with Pseudogene.org. However there is no detailed explanation why there are over 3500 pseudogenes are not detected by this method of alignment using blast or CDUAlign and is there anything specific about these pseudogenes, are they all 1:1 relationship with parent gene?

**The reviewer identifies an important area, which we have been working on since the initial submission. We have found that a significant portion of these pseudogenes/genes that are in the newer version of the Pseudogenes.org database are not contained in our GENCODEv25 annotation. These missing genes and pseudogenes account for 1030 of the 2458 pseudogene-gene pairs that are in Pseudogenes.org but not in our databases. If these are excluded we recreate 85% of the Pseudogenes.org pseudogene-gene pairs (1:1 relationships). Furthermore this 85% accuracy is similar to our benchmarking accuracy (87%) on genes whose annotation will likely not change drastically between annotation builds. Alternatively, since these genes and pseudogenes were from a different annotation version the sequences themselves could be slightly different causing differences between our database and Pseudogenes.org. These results can be found in the Results section "Direct comparison to pseudogene parents".**

**In Results paragraph 5:**

**"Our databases also generate a larger pool of possible interactions. It is worth noting that 391 pseudogenes and 152 genes in the new Pseudogene.org (GENCODEv10) are not present in the GENCODEv25 annotation used in our analysis. These genes and pseudogenes together account for 1030 edges that were used in our comparison. Accounting for these differences in the annotation, we are**

**able to reconstruct 85% of the pseudogene-gene relationships in the new Pseudogene.org database. Since these associations were generated without prior pseudogene-gene relationship information and the annotations have changed slightly since Pseudogenes.org, our methods prove to independently identify known and unknown pseudogene-gene relationships at a high rate."**

3) For the use case example, I do not fully understand why the CDUAlign18 was used for PPPARIL identification in sox15 and not detected in the CDUAlign54 or CDUAlign135. Looking at the sox15 network using CDUAlign135 an alternative pseudogene PIN2 pseudogene can be found. Can the authors explain why this is not also considered as potential regulator and why it does not appear in the TGCA expression panel with the rest of the sox genes ?

**We thank the reviewer for their insight and have further evaluated the SOX15 network in response. RP11-506B6.5, the pseudogene located next to PIN2, is retained in the more stringent databases (e.g, CUDAlign135) and as such should also be considered. However, RP11-506B6.5 lacks enough annotation from existing literature to make it a promising candidate. The PPPARIL gene has more supporting literature and is a larger more complex pseudogene containing 19 exons opposed to 1 exon in RP11-506B6.5.**

4) Since the usability of the web app is highlighted in the paper, I would recommend a direct link from the Ensembl Identifiers to Ensembl rather than Genecards eg ENST00000428294 does not have a Genecard entry but is classified as a transcribed unprocessed pseudogene by GENCODE/Ensembl.

**We thank the reviewer for this suggestion and as a result have added this functionality to the website. When a user selects a network node, a tab for GeneCards and a tab for Ensembl appears for the specified gene/pseudogene.**

5) Also the network in the webapp would be easier to navigate if the HGNC identifier was used as default name rather than the ENSG ID (as this should be relatively easy to code) and therefore recommend figure 4 be redrawn as looks extremely hard to interpret.

**We appreciate these suggestions and have focused on our main application that is available online (https://integrativeomics.shinyapps.io/pseudofun_app/). In this web application HGNC identifiers are used throughout. The old Figure 4 is from another application we developed for research purposes through the Ohio Supercomputer Center. As a result, we moved the old Figure 4 to the supplementary material so that it does not detract from the usability of the main application (which has much more improved user-friendly visualization) freely available online.**

6) Fig 4 should have details of the CDU align cut off used in the legend for the network graphs similar to fig 3

**We thank the reviewers for their concerns and have added this information to Figure 4, which has been moved to the supplementary material as Supplementary Figure 2. We feel that this figure is of less importance after running the benchmarking experiment, shown in Table 2.**

Minor issues:

* Pg12 line 12 "regulation" typo

* Pg 16 sentence should have "network" inserted before gene on line

**We appreciate the help from the reviewer for identifying language errors in the manuscript and have made the changes.**
