# Supplementary material for: PseudoFuN: Deriving functional potentials of pseudogenes from integrative relationships with genes and microRNAs across 32 cancers
Source: Gigascience. 2019 Apr 26;8(5):giz046. doi: 10.1093/gigascience/giz046 (PMC6486473; doi:10.1093/gigascience/giz046)
Supplement: Supplemental File [file giz046_supplemental_file.docx]

**PseudoFuN: Deriving functional potentials of pseudogenes from integrative relationships with genes and miRNAs across 32 cancers**

**Supplementary Materials**

***Data Sources***

TCGA^1^ data: <http://gdac.broadinstitute.org/>

dreamBase^2^: <https://www.synapse.org/#!Synapse:syn11461899/files/>

psiDr^3^ (“old” in text): <pseudogenes.org/psidr/similarity.dat>

PsiCube^4^ (“new” in text): <pseudogenes.org/psicube/data/gencode.v10.pseudogene.txt>

GENCODE^5^: <https://www.gencodegenes.org/releases/25.html>

Ensembl Biomart^6^ (Feb 2017): <ensembl.org/biomart/martview>

***Modeling ceRNA gene and pseudogene quantities***

We want to show the current understanding of how ceRNA networks including pseudogenes would affect mRNA abundance^7-10^. To show this we include i) a graphical presentation of a ceRNA network including crosstalk between pseudogenes and genes through competitive binding to miRNAs (Supplementary Figure 1A), ii) a cellular model of ceRNAs in the cell (Supplementary Figure 1B), and iii) a simplistic mathematical model of ceRNA abundances (Supplementary Figure 1C).

In the graphical ceRNA network view (Supplementary Figure 1A) we see that for every 1 miRNA there are three genes and two pseudogenes (just for this example) that can competitively bind to this miRNA. These numbers are hypothetical but are used because they are simple enough to directly show the mechanism. We suppose the genes have the same binding affinity to the miRNA, i.e., likelihood that an miRNA in proximity to the gene will bind and eventually degrade the gene. Similarly, we suppose the pseudogenes all have the same binding affinity, which can be the same or different than the gene binding affinity. Despite them most likely being different and considering the fact that the genes and pseudogenes in the example are highly homologous, the binding affinities are most likely not independent of each other. The binding affinities as described above are considered a probability. The miRNA in the graphical example will be the closest to one of the 5 transcripts (3 genes and 2 pseudogenes). The miRNA then has a probability of binding to that transcript and disposing of it. To increase the simplicity of the model we consider one step in a dynamic system where miRNA is in proximity to a transcript and has a probability of binding and disposing of that bound transcript. We also assume that two miRNAs are not closest in proximity to a single transcript. We recognize that in reality there would be rates of miRNA production, differential rates of binding, etc. However to denote the simplest ceRNA network model possible, which can be expanded upon, we use these assumptions.

In the cellular view of the model (Supplementary Figure 1B), the grey circle is the nucleus and the color-coded lines denote miRNAs, gene mRNAs, and psuedogene mRNAs with the same color coding as in Supplementary Figure 1A. We demonstrate the binding between miRNA and mRNAs by cross (X), and the proportions of molecules are consistent with those in Supplementary Figure 1A.

To take the simplest model of pseudogene ceRNA networks further, we then convert the graphical view and cellular view into equations for caculating the final number of unbound mRNA transcripts for gene and pseudogene respectively (Supplementary Figure 1C). Using these equations we do an exhaustive simulation. We assume there are between 1-5 miRNAs, 1-10 genes, 1-10 pseudogenes, 0.01-1.00 gene binding affinity, and 0.01-1.00 pseudogene binding affinity. We then generate the final number of genes and the final number of pseudogenes for each of these settings resulting in two 5-dimensional tensors for gene and pseudogene respectively.

Next we take the correlation between the final gene mRNA count tensor and final pseudogene mRNA count tensor across the 1-10 initial pseudogene amount. This process results in a 4-dimensional tensor of final gene-final pseudogene count correlations. If we plot a probability density function (PDF) of these correlations we find that they are all positive and most quite high (Supplementary Figure 1D). We also wanted to show that the starting amount of pseudogene modulates the change in gene – the reduction of unbound gene transcripts is nonlinear due to the amount of pseudogene (Supplementary Figure 1E). To generate these results we use a static gene amount of 5 transcripts, and a static miRNA amount of 1-5, with a dynamic pseudogene amount 1-10. We plot the change in gene count 5 minus the number of miRNA-gene bindings (y-axis) by the number of starting pseudogenes 1-10. The resultant plot has 5 lines (number of miRNAs) containing 10 points (the number of pseudogene transcripts). Note the change is not an integer but for simplicity we consider it as a hypothetical abundance. Future models will most likely use negative binomial or Poisson distribution to account for count data. Alternatively we may model the binding using more sophisticated differential equation models. In the results we see that the number of starting pseudogene transcripts modulates the effect of the miRNAs on the final number of gene transcripts (Supplementary Figure 1E).

***Ohio Supercomputing Center web application***

Through a partnership with the Ohio Supercomputing Center^11^ (OSC), we developed a web application using the OSC OnDemand^12^ platform so that users could easily query our databases and download both processed and raw results (Supplementary Figure 7). The resulting application can run both sequence and Ensembl gene ID queries against any of our databases (Supplementary Figure 7A). These queries return interactive minimal spanning trees that display the relationships within each PGG family network (Figure 4, Supplementary Figure 7B). The application also contains the Gene Ontology^13^ (GO) information for all of the genes in the PGG family networks. If the user needs to perform analyses on the raw PGG family alignment matrices, there is an option do download these alignment matrices as CSV files.

***Potential regulatory roles in cancer***

In Use Cases III (see main text), we have analyzed the list of DE parent genes (of pseudogenes) potentially regulated by miR-96 in prostate cancer. Although our DE genes were detected from prostate cancer^14^, we further compared them with DE pseudogenes identified in four other cancer types^15^. As a result, we identified 11 DE parent genes/pseudogenes in BRCA, 8 DE parent genes/pseudogenes in GBM, 10 DE parent genes/pseudogenes in LUSC, and 4 DE parent genes/pseudogenes in UCEC that were contained within PGG families (54 cutoff) regulated by miR-96 (Supplementary Table 1). The families containing these pseudogenes could be of interest due to the possible regulation occurring in the cancerous samples. Besides, we also found potential interaction between GBP1P1 and miR-96 (potentially compete for miRNA) from Han et al.^15^ (Supplementary Figure 8) We show GBP1P1 as a possible interacting pseudogene with miR-96 using PseudoFuN app (Figure 4D, pearson rho= -0.16 and FDR = 0.003)

**Supplementary Figures**

Supplementary Figure 1. Example of ceRNA network regulation of gene expression. A) A graphical view of how pseudogene expression can regulate gene expression. B) A cellular view of ceRNA network regulation. C) Equations used to model the correlation between gene and pseudogene expression in a ceRNA network. D) The distribution of the gene-pseudogene correlations based on the models in C. E) The effect that pseudogene expression has on the miRNA induced change in gene expression.

Supplementary Figure 2. Representative examples of our OSC OnDemand pseudogene query tool. Displayed are the network relationships from our databases for three common ceRNA network examples (queries: FTH1, KRAS, PTEN), and a relationship of interest (GBP1-GBP1P1) using the BlastDB for consistency.

Supplementary Figure 3. PseudoFuN online output for PTEN PGG family. A) Interactive graph visualization of the PTEN PGG network. B) TCGA breast cancer co-expression matrix for PTEN PGG family genes and pseudogenes across normal samples. C) TCGA breast cancer co-expression matrix for PTEN PGG family genes and pseudogenes across tumor samples. D) Negatively correlated miRNAs for all members of the PTEN PGG family in breast cancer. E) Differential gene and pseudogene expression for tumor and normal samples for each member of the PTEN PGG family in the prostate cancer TCGA dataset.

Supplementary Figure 4. PseudoFuN online output for HTR7 PGG family. A) Interactive graph visualization of the HTR7 PGG network. B) TCGA prostate co-expression matrix for HTR7 PGG family genes and pseudogenes across normal samples. C) TCGA prostate co-expression matrix for HTR7 PGG family genes and pseudogenes across tumor samples. D) Negatively correlated miRNAs for all members of the HTR7 PGG family. E) Differential gene and pseudogene expression for tumor and normal samples for each member of the HTR7 PGG family in the prostate cancer TCGA dataset.

Supplementary Figure 5. PseudoFuN online output for CNN2/TAGLN2 PGG family. A) Interactive graph visualization of the CNN2/TAGLN2 PGG network. B) TCGA prostate co-expression matrix for CNN2/TAGLN2 PGG family genes and pseudogenes across normal samples. C) TCGA prostate co-expression matrix for CNN2/TAGLN2 PGG family genes and pseudogenes across tumor samples. D) Negatively correlated miRNAs for all members of the CNN2/TAGLN2 PGG family. E) Differential gene and pseudogene expression for tumor and normal samples for each member of the CNN2/TAGLN2 PGG family in the prostate cancer TCGA dataset.

Supplementary Figure 6. PseudoFuN online output for MSN PGG family. A) Interactive graph visualization of the MSN PGG network. B) TCGA prostate co-expression matrix for MSN PGG family genes and pseudogenes across normal samples. C) TCGA prostate co-expression matrix for MSN PGG family genes and pseudogenes across tumor samples. D) Negatively correlated miRNAs for all members of the MSN PGG family. E) Differential gene and pseudogene expression for tumor and normal samples for each member of the MSN PGG family in the prostate cancer TCGA dataset.

Supplementary Figure 7. The PGG families in our network with the most DE genes after miR-96 treatment. The line weights indicate the sequence homology between members of the PGG family. Red nodes indicate miR-96 targets. Yellow nodes with names indicate other genes contained in the PGG family. Yellow nodes without names are pseudogenes contained within the network.

Supplementary Figure 8. The user interface of the OSC OnDemand web application. A) is the main query page where a user can search either sequences or ensemble gene IDs. B) is a representative output of one of the gene searches. This includes an interactive network and the GO information.


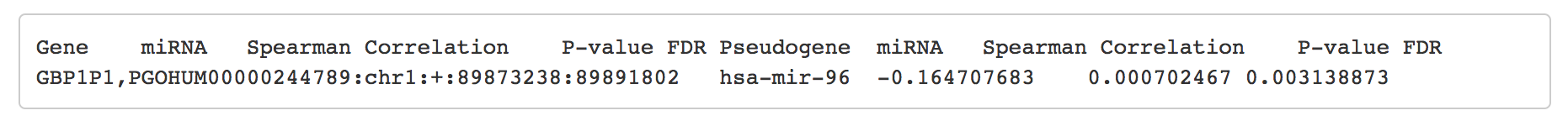


Supplementary Figure 9. GBP1P1 DE in TCGA prostate cancer (information retrieved from Han et al.).

**Supplementary Tables**

Supplementary Table 1. DE parent gene/pseudogenes potentially regulated by miR-96 in prostate cancer vs. TCGA derived DE pseudogenes.

| **Cancer** | **DE parent genes regulated by miR-96 derived from our previous study^14^** | **DE pseudogenes derived from TCGA^15^** |
| --- | --- | --- |
| BRCA | NAPSB  GBP1  CXCR2  CES1  ACTN4  LGALS1  ACTB  FNBP1  TRIM6  ANXA2  RIMKLB | NAPSB,PGOHUM00000263658,PGOHUM00000263659:chr19:-:50837053:50848024  GBP1P1,PGOHUM00000244789:chr1:+:89873238:89891802  CXCR2P1,PGOHUM00000240523:chr2:-:218923878:218930452  CES1P1,PGOHUM00000250686,PGOHUM00000250685,PGOHUM00000262340:chr16:+:55794460:55828070  ACTN4P1,PGOHUM00000246045:chr4:+:117519317:117520767  LGALS17A,PGOHUM00000234695:chr19:+:40170014:40177013  ACTBP11,PGOHUM00000244448:chr1:-:224051428:224052553  FNBP1P1,PGOHUM00000240689:chr2:+:74347807:74350349  TRIM60P18,PGOHUM00000233356:chr7:+:63815396:63816577  ANXA2P3,PGOHUM00000238817:chr10:+:66585317:66586338  RIMKLBP2,PGOHUM00000244422:chr1:-:219373254:219374040 |
| GBM | DUSP5  NAPSB  GBP1  ACTN4  ACTB  FNBP1  TRIM6  ANXA2 | DUSP5P1,PGOHUM00000245243:chr1:+:228744885:228788150  NAPSB,PGOHUM00000263658,PGOHUM00000263659:chr19:-:50837053:50848024  GBP1P1,PGOHUM00000244789:chr1:+:89873238:89891802  ACTN4P1,PGOHUM00000246045:chr4:+:117519317:117520767  ACTBP9,PGOHUM00000235085:chr18:+:60109242:60110359  ACTBP7,PGOHUM00000246771:chr15:-:44281259:44282382  ACTBP11,PGOHUM00000244448:chr1:-:224051428:224052553  FNBP1P1,PGOHUM00000240689:chr2:+:74347807:74350349  TRIM60P18,PGOHUM00000233356:chr7:+:63815396:63816577  ANXA2P3,PGOHUM00000238817:chr10:+:66585317:66586338 |
| LUSC | DUSP5  NAPSB  GBP1  CXCR2  CES1  ACTN4  ACTB  FNBP1  TRIM6  ANXA2 | DUSP5P1,PGOHUM00000245243:chr1:+:228744885:228788150  NAPSB,PGOHUM00000263658,PGOHUM00000263659:chr19:-:50837053:50848024  GBP1P1,PGOHUM00000244789:chr1:+:89873238:89891802  CXCR2P1,PGOHUM00000240523:chr2:-:218923878:218930452  CES1P1,PGOHUM00000250686,PGOHUM00000250685,PGOHUM00000262340:chr16:+:55794460:55828070  ACTN4P1,PGOHUM00000246045:chr4:+:117519317:117520767  ACTBP11,PGOHUM00000244448:chr1:-:224051428:224052553  ACTBP9,PGOHUM00000235085:chr18:+:60109242:60110359  FNBP1P1,PGOHUM00000240689:chr2:+:74347807:74350349  TRIM60P18,PGOHUM00000233356:chr7:+:63815396:63816577  ANXA2P3,PGOHUM00000238817:chr10:+:66585317:66586338 |
| UCEC | NAPSB  GBP1  CES1  TRIM6 | NAPSB,PGOHUM00000263658,PGOHUM00000263659:chr19:-:50837053:50848024  GBP1P1,PGOHUM00000244789:chr1:+:89873238:89891802  CES1P1,PGOHUM00000250686,PGOHUM00000250685,PGOHUM00000262340:chr16:+:55794460:55828070  TRIM60P18,PGOHUM00000233356:chr7:+:63815396:63816577 |

Supplementary Table 2. High homology pseudogenes with many alignments over Smith-Watermann pairwise alignment score of 54 (99th percentile of all alignment scores).

|  | **Pseudogene symbols** |
| --- | --- |
| **Top 9 pseudogenes (over 15,000 alignments above 54)** | UBE2Q2P1, RP11-313J2.1, TPTEP1, BMS1P1, CTD-2245F17.3, SCAND2P, GTF2IP7, WHAMMP3, IGLV3-2 |
| **Top 571 pseudogenes (over 1,000 alignments above 54)** | NPAP1P2, ARHGAP42P4, RPL30P11, AC009967.3, MTHFD2P1, RP11-486A14.1, FAM92A1P2, RP11-648O15.1, RP11-270M14.1, ACTG1P17, ARHGAP42P5, RP11-1016B18.1, COX4I1P2, NPAP1P3, CTD-2311M21.2, ZIK1P1, AC110926.4, FTLP10, HNRNPA1P40, RP11-791G16.2, OLA1P2, RP11-473P24.2, INGX, RPS11P6, RP11-333E13.4, AMD1P4, RP11-346I3.2, RPLP0P2, LRRC37A9P, RP11-480I12.9, RP11-347J14.4, RP11-820K3.3, RPL7P41, HSP90AB7P, Z97634.3, RP11-10O17.1, RP11-531F16.3, LINC01296, SHC1P2, HNRNPA1P54, CTD-2651B20.5, RP11-791G16.2, RP4-701O16.5, EIF3EP2, RP13-130D24.1, KB-1980E6.2, RP11-44M6.3, AC012454.4, RP11-288H12.3, MTHFD2P1, LINC01057, AC073065.3, AC083899.1, RP11-597G23.1, RP11-209M4.1, CCDC144CP, RP11-34P1.1, OR11J5P, LINC01296, RPSAP36, RP11-614F17.1, AC016712.1, AC098614.2, RP11-208P4.1, MTND1P10, RP11-15G8.1, ZNF702P, RP11-229P13.2, ZNF37BP, RP11-570J4.2, RPS2P39, PGAM1P12, AC006509.7, RP11-597D13.7, AC093838.4, HMGB1P8, RPL21P135, GUSBP5, RP11-617J18.1, MRPS17P1, GPAA1P2, IARS2P1, RPL29P25, RP11-820K3.2, RP5-961K14.2, FNBP1P1, ZNF90P3, RP11-501C14.7, RP11-570L14.1, UNGP1, RP11-365F18.3, MTATP8P2, RP11-616L12.4, RP11-364B6.2, LRRC37A7P, ZNF969P, AC013474.4, RP11-386I23.1, DUXAP9, VENTXP4, RP11-331G2.6, TCEB1P33, LINC01296, TDGF1P3, RP11-74M13.4, PSMD10P2, AC074019.2, DDX50P1, AC019109.1, RP11-266L9.1, RP11-730A19.5, RP11-390M11.1, SLC9A7P1, RPL23AP87, HSPE1P10, RP5-1000K24.2, AC073551.1, RP1-283E3.4, RP11-804M7.1, HMGN2P46, RP11-307A17.2, CTD-2583A14.11, CCNB3P1, CEP164P1, RP3-499B10.3, POM121L7, AC010886.2, OLA1P3, HMGB1P39, RPS20P22, FTLP6, RP11-809N15.3, AC005154.7, LINC01296, GAPDHP72, CCDC144CP, ZNF37BP, ZNF321P, RPS2P45, E2F3P2, CTC-543D15.1, CTD-2521M24.4, KLF2P4, IPO5P1, BTBD7P1, DUXAP9, FAM74A4, RP5-859M6.1, YWHAQP5, AC008132.13, NIFKP4, RP11-1348G14.1, ODCP, RP11-58E21.1, ETF1P2, LINC01296, RP11-805L22.2, RP11-2I17.1, DUXAP9, RP1-34L19.1, RP11-483G21.3, RP11-382D8.5, PSMD10P2, RBM22P2, LDHBP1, HTR7P1, RP4-569D19.5, RP11-354K4.1, CTD-2547L16.3, MKRN9P, CENPBD1P1, BNIP3P37, RP11-762H8.2, BNIP3P28, CICP27, AC111200.2, CTC-539A10.7, RP11-169L17.3, RP11-266I3.1, RP1-296G17.3, RPL21P122, METTL21EP, RRN3P3, GVINP1, ZNF826P, HERC2P5, CYP4F30P, ABCA17P, MTND2P12, NBR2, SKINT1L, CTC-559E9.6, TPTEP1, OR6K1P, LINC01347, ADAM5, APOC1P1, NSUN5P1, ABCA17P, HERC2P5, UBE2Q2P1, LINC00674, FAM197Y3, CTB-167G5.6, GTF2H2B, FLJ42102, RRP7BP, TP73-AS1, RP11-568K15.1, CRYBB2P1, TPTEP1, SNX29P2, AP005901.1, AC010980.2, SUGT1P3, LINC00643, NSUN5P2, GSTTP1, TP73-AS1, RP11-252A24.2, DPY19L1P1, CTD-2026D20.3, RP3-449O17.1, PI4KAP1, AC027612.3, RSU1P2, PI4KAP1, SUZ12P1, NOS2P3, NSUN5P2, RP11-33B1.1, ADGRE4P, PI4KAP2, RP1-274L7.4, LPAL2, CTD-2542C24.2, CELP, PI4KAP2, RPL23AP82, FAM197Y9, RP3-449O17.1, ZNF826P, ANKRD36BP2, CTSLP2, CARM1P1, NBR2, RP11-680E19.1, GYG2P1, ADAM3A, HERC2P4, RPL32P3, GTF2IP13, CTD-2554C21.2, RP11-313J2.1, FAR2P1, RP11-153M7.3, NSUN5P2, TPTE2P2, MRPS31P5, KRT42P, STAG3L2, CH17-472G23.2, RP11-465B22.3, LINC00888, CSPG4P12, RRP7BP, CYP2B7P, GTF2IP12, RP11-10G12.2, RP11-291L22.9, PI4KAP2, USP32P2, POLR2J4, HERC2P8, HERC2P9, FAM197Y7P, DNM1P46, ZNF271P, CRYBB2P1, RP11-760D2.13, SRP54-AS1, RP4-800G7.2, CTB-159G17.3, GTF2IP20, CH17-472G23.2, PRORSD1P, SEPT7P2, HERC2P3, CTD-2245F17.3, RPL32P3, SPATA31C1, MGC2752, RSU1P2, RP11-114G11.3, RP3-449O17.1, FAM86DP, GTF2H2B, RP11-551L14.4, FAM197Y2P, CIDECP, CCDC162P, RP11-423H2.1, CH17-125A10.1, RP11-483E23.2, PMCHL2, ZNF826P, NBEAP1, RP11-61L23.2, CCDC144B, WHAMMP2, RP11-231P20.2, FAR2P4, ANAPC1P1, TPTE2P2, CROCCP2, HSD17B7P2, AP000347.2, STAG3L3, CATSPER2P1, GOLGA8IP, RP11-566K19.12, LRRC37A4P, PMCHL2, AC016582.2, RP11-206L10.2, OR7E94P, TP73-AS1, STAG3L5P, FKBP9P1, RPS6P20, SEPT7P2, AC005176.2, TP73-AS1, TCAM1P, PLEKHM1P1, KRT42P, HLA-V, CTC-281F24.3, CYP4F30P, CIDECP, ABC7-42418200C9.1, PLEKHM1P1, CTB-134H23.3, CCDC144B, GTF2IRD2P1, CMB9-22P13.1, ANKRD30BP2, TPTEP1, RP11-480I12.5, RP11-113D6.10, RP11-339B9.1, SMPD4P1, CD99P1, LRRC37BP1, USP9YP4, LRRC37A5P, MED15P9, FAM197Y6, CRYBB2P1, HERC2P3, RRN3P1, RP4-673D20.3, PI4KAP2, ZNRD1ASP, RP11-381O7.3, RP11-33B1.1, PCDHGB8P, CYP2B7P, PKD1P6, AC141586.5, SUZ12P1, PNLIPP1, GS1-124K5.2, SUZ12P1, ULK4P1, FER1L4, KRT42P, AC136289.1, ABCC13, ADAMTS7P4, LINC00933, ZNF542P, EMC3-AS1, CROCCP3, RP11-726G1.1, FAM86B3P, PMCHL1, FAM86B3P, PRKY, USP32P1, ZNF252P, GTF2IP1, TBC1D27, TPTE2P6, RP11-646I6.3, AC074141.3, LINC00888, RP11-23E10.4, RP11-61L23.2, ZNF962P, GNRHR2, CCT6P3, CRYBB2P1, CCDC144B, APOC1P1, TPTEP1, USP32P1, GNRHR2, MTMR9LP, AC011380.8, GTF2IRD2P1, AC009237.8, TMPRSS11BNL, HLA-L, FAR2P4, AC092933.4, NSUN5P1, ZNF542P, GUSBP3, GOLGA2P5, AC129778.2, RP11-586D19.1, TPTEP1, ZNF542P, RP11-483E23.2, BTN2A3P, FER1L4, CEACAM22P, RP11-124D2.7, BMS1P1, CATSPER2P1, CTD-2245F17.3, PI4KAP1, ALOX12P2, OR13I1P, RP11-113D6.10, RPL32P3, ZNF542P, RPL32P3, FAR2P3, AFG3L1P, TMPRSS11BNL, CD99P1, SCAND2P, RP11-480I12.5, OR4F13P, AC016582.2, CASP16P, SPATA1, NANOGP1, EGFEM1P, WHAMMP3, CTC-281F24.3, STAG3L3, DMBT1P1, FER1L4, CROCCP2, CTD-2026D20.3, FER1L4, GUSBP2, GTF2IP7, ACTBP13, PDCD6IPP2, STAG3L5P, DSTNP2, MTCO3P21, SULT1C2P1, ULK4P3, RP11-606M12.1, RP11-231P20.6, STAG3L3, CHIAP2, GTF2IP11, TBC1D27, FMO9P, PCDHB19P, SCART1, KRT42P, RP11-44F14.1, DGCR5, SUZ12P1, NSUN5P1, TUBA3FP, ZNF252P, FAM157A, CLCN3P1, BCRP2, RRN3P1, MGAT4EP, HERC2P8, MTND4P30, ADAM1B, MTCO1P25, CROCCP3, ZDHHC8P1, ZDHHC8P1, RASA4CP, SEPT7P2, WHAMMP3, AOC4P, TP73-AS1, RP11-848P1.9, USP9YP3, PLEKHM1P1, PARGP1, RP11-252A24.2, CTB-159G17.4, RP11-460N11.2, CTD-2014N11.1, WHAMMP2, AC093642.5, NSUN5P1, OR4A14P, USP32P2, RPL32P3, EMC3-AS1, RBMY2FP, CATSPER2P1, STAG3L2, TPTE2P1, ZNF876P, FAM21EP, SEC1P, GTF2IP1, PI4KAP2, AC105339.1, RP11-492D6.3, FAM157A, AC016582.2, AC091493.1, RP11-44D5.1, CLUHP3, STAG3L3, PI4KAP1, RP4-800G7.2, FGD5P1, UBE2Q2P2, PDXDC2P, PGM5P2, AC009237.8, ANKRD30BP2, AOC4P, CPHL1P, CYP4F62P, RP11-260N14.1, IGLV3-31, IGKV2-4, FCGR2C, IGHV3-25, IGHVIII-67-3, AL691477.1, IGHVII-60-1, 257-685, IGHVII-49-1, IGKV2OR22-4, IGHVII-31-1, TRBV26OR9-2, RP11-4L24.4, IGKV1-13, AC007325.3, RP5-875O13.1, AL353898.1, IGLV3-2, IGKV2D-23 |

**References**

1. Cancer Genome Atlas Research N, Weinstein JN, Collisson EA, et al. The Cancer Genome Atlas Pan-Cancer analysis project. *Nat Genet.* 2013;45(10):1113-1120.

2. Zheng LL, Zhou KR, Liu S, et al. dreamBase: DNA modification, RNA regulation and protein binding of expressed pseudogenes in human health and disease. *Nucleic Acids Res.* 2018;46(D1):D85-D91.

3. Pei B, Sisu C, Frankish A, et al. The GENCODE pseudogene resource. *Genome Biology.* 2012;13(9):R51.

4. Sisu C, Pei B, Leng J, et al. Comparative analysis of pseudogenes across three phyla. *Proc Natl Acad Sci U S A.* 2014;111(37):13361-13366.

5. Harrow J, Frankish A, Gonzalez JM, et al. GENCODE: the reference human genome annotation for The ENCODE Project. *Genome Res.* 2012;22(9):1760-1774.

6. Zerbino DR, Achuthan P, Akanni W, et al. Ensembl 2018. *Nucleic Acids Res.* 2018;46(D1):D754-D761.

7. Chan JJ, Tay Y. Noncoding RNA:RNA Regulatory Networks in Cancer. *Int J Mol Sci.* 2018;19(5).

8. Poliseno L, Pandolfi PP. PTEN ceRNA networks in human cancer. *Methods.* 2015;77-78:41-50.

9. Xu J, Feng L, Han Z, et al. Extensive ceRNA-ceRNA interaction networks mediated by miRNAs regulate development in multiple rhesus tissues. *Nucleic Acids Res.* 2016;44(19):9438-9451.

10. Yang C, Wu D, Gao L, et al. Competing endogenous RNA networks in human cancer: hypothesis, validation, and perspectives. *Oncotarget.* 2016;7(12):13479-13490.

11. Center OS. Ohio Supercomputer Center. Columbus OH: Ohio Supercomputer Center; 1987.

12. Hudak D, Johnson D, Chalker A, et al. Open OnDemand: A web-based client portal for HPC centers.

13. Ashburner M, Ball CA, Blake JA, et al. Gene Ontology: tool for the unification of biology. *Nature genetics.* 2000;25(1):25.

14. Long MD, Singh PK, Russell JR, et al. The miR-96 and RARgamma signaling axis governs androgen signaling and prostate cancer progression. *Oncogene.* 2018.

15. Han L, Yuan Y, Zheng S, et al. The Pan-Cancer analysis of pseudogene expression reveals biologically and clinically relevant tumour subtypes. *Nat Commun.* 2014;5:3963.
