# Supplementary material for: PseudoFuN: Deriving functional potentials of pseudogenes from integrative relationships with genes and microRNAs across 32 cancers
Source: Gigascience. 2019 Apr 26;8(5):giz046. doi: 10.1093/gigascience/giz046 (PMC6486473; doi:10.1093/gigascience/giz046)
Supplement: Response_to_Reviewer_Comments_Original_Submission.pdf [file giz046_response_to_reviewer_comments_original_submission.pdf]

Author's Response To Reviewer Comments

Close

Response Letter (We recommend reading the pdf version Giga_reviews_20181212.pdf for clearer format.)

We thank the reviewers for their insightful comments and believe that after addressing each comment the manuscript is stronger. Please see the reviewers' comments and our responses below to see specifically how all of the concerns were addressed. We have highlighted all our answers in red color. We also highlight in red color all the changes in the main text.

Reviewer #1: The role of pseudogenes in the modulation of gene regulation is a burgeoning field that is ideally placed to benefit from integrative approaches that utilise "big data" that is currently available. A user friendly tool such as PseudoFun is therefore of use as a possible discovery mechanism for new relationships. Not having used PsuedoFun at this stage, it is difficult to fully evaluate its performance, though the approach described appears useful and the presentation of new relationships such as that suggested between PPP4RiL, SOX15 and miR-375 highlight a potential to identify new avenues for further investigation. I have only minor suggestions for improvement in presentation.
1) In Figure 5 (and much of the supplementary figures presented in a similar fashion), is the miRNA associated directly targeting the gene/pseudogene. Visually, only a correlative expression relationship is indicated.

Answer: We appreciate the reviewer's feedback. In Figure 5 (currently Figure 4), the miRNAs associated with gene/pseudogene were determined by not only expression correlation but also miRNA target prediction databases: Miranda, PicTar and TargetScan. We downloaded the predictions from http://gdac.broadinstitute.org. According to the reviewer's comment, we have improved the miRNA section on the TCGA Expression panel of the website. The website now allows the user to select how many algorithms predict regulation of the gene/pseudogene by the miRNA. This value is used as a threshold for the displayed miRNAs. The default is 0 meaning that the miRNA and gene/pseudogene are significantly negatively correlated indicative of possible regulation. The value can be changed from 0-3 indicating the number of algorithms (Miranda, PicTar, and TargetScan) predict regulation of gene/pseudogene by the specified miRNA.

2) Figure 4 does little to add clarity. If the goal is to highlight regulatory relationships, the ENSTxxx labelling does not lend for easy interpretation and the miRNAs are not shown. If the intended purpose is to illustrate a style at which data is outputted, perhaps this is better served by a user friendly series of screenshots illustrating a beginning to end data query - result flow?

Answer: We agree with the reviewer that containing only ENSTxxx labelling does not facilitate illustration. We use easy-to-interpret gene names and links to other gene databases (e.g. GeneCards, Ensembl) to improve the usability of our PseudoFuN website (https://integrativeomics.shinyapps.io/pseudofun_app/), and we have improved the visualization according the reviewers' suggestions. The old Figure 4 is not from our public PseudoFuN version, instead it is from our supercomputer version located on the Ohio Supercomputer Center clusters and is meant mainly for research purposes. Since it is not exactly the version we mainly presented in the main text, which has much more user-friendly interface with interpretable gene names, we moved the old Figure 4 to the supplementary materials so that it does not detract from the usability of the main application freely available online.

3) In Figure 5 and some supplementary figures, co-expression visually is not well represented by the colour scheme. ie: the tumour relationship between PPP4R1L and SOX15. The stats support this, the visual representation less so. Perhaps blanking out the 1:1 same gene : same gene diagonal would allow re-setting of the colour scheme to better represent co-expression?

Answer: This insightful comment by the reviewer caused us to rethink our visualization. We changed the same gene/pseudogene correlation (1.0) along the diagonal line in the heatmap to NaN so that the

visualization ignores those values. This allows the range of color for the other correlations to be more diverse and more informative to the users.

4) In paragraph 2 of the results, I was unclear what the "alignment score above 54" means... What degree of alignment is this? I found understanding this to be hard to gauge. Relating to this, could the authors comment more extensively on their findings of tremendous levels of alignment for some pseudogenes?

Answer: We agree with the reviewer that we should more fully explain the alignment scores in the manuscript and as a result explain in more detail what the alignment scores represent in paragraph 2 of the results:
“We evaluate alignment of pseudogenes to genes using the Smith-Watermann local pairwise alignment score56 between a pseudogene and a gene. These scores indicate the highest score possible for two sequences based on their specific dynamic programing matrix which is solved by the Smith-Watermann algorithm. The cutoffs we use, 18, 54, 135, and 198, indicate the 97.50th, 99.0th, 99.90th and 99.99th percentiles of alignment scores in our alignment matrix between all pseudogenes and consensus sequences.”

We have also performed more in-depth analysis on the high homology pseudogenes and described these findings in more detail in paragraph 2-3 of Results. Specifically, we found zinc finger pseudogenes and other domain binding patterns in the highest homology pseudogenes. We found large bodies of evidence that the high homology pseudogenes have either direct or indirect relationships with zinc finger genes.
In Results paragraph 2:
“Another feature of note is that there are some pseudogenes that align to many gene families (e.g., 9 pseudogenes, UBE2Q2P1, RP11-313J2.1, TPTEP1, BMS1P1, CTD-2245F17.3, SCAND2P, GTF2IP7, WHAMMP3, IGLV3-2, have alignment scores above 54 in 15,000 gene families and 571 pseudogenes, see Supplementary Table 2, have alignment scores above 54 in 1,000 gene families).”
In Results paragraph 3:
“Of the 9 highest homology pseudogenes (Supplementary Table 2), one, RP11-313J2.1, is a zinc finger pseudogene and two, CTD-2245F17.3 and SCAND2P, are located in the promoters of zinc finger genes. Four pseudogenes in the 9 highest homology pseudogenes (RP11-313J2.1, CTD-2245F17.3, SCAND2P, and WHAMMP3) also have 92-96% sequence identity with zinc finger genes (ZNF72P, ZNF518A, ZNF37A and ZNF788P/ZNF20 respectively) when BLAST searched against the human genome. Of the 571 highest homology pseudogenes (Supplementary Table 2), we found 27 zinc finger pseudogenes. Using EnrichR59 we identified enrichment in GO Molecular Function GO:0004430 1-phosphatdylinositol 4-kinase activity (Fisher’s exact test p-value = 0.001), and enrichment for GO Biological Process GO:0070475 rRNA base methylation (Fisher’s exact test p-value = 0.003). In the ARCHS4 database60 324 transcription factors were significantly co-expressed (Benjamini-Hochberg adjusted Fisher’s exact test p-value < 0.05) with members of the 571 highest homology pseudogenes. Of those 324 transcription factors, 228 were zinc finger genes. These findings show that the highest homology pseudogenes, like zinc finger genes, likely contain repetitive elements that align to many genomic loci.”

Reviewer #2: The authors have presented an overview of their new analysis and data resource to identify novel pseudogene-gene network interactions that could lead to new hypothesis around their role in regulation of cancer using TCGA cancer expression data and miRNA expression. The unique element of this analysis is using a consensus sequence representing gene families and examining the local alignment of pseudogenes against this consensus to identify new potential interactions.

The major criticism of the paper is that a thorough benchmarking evaluation of their different alignment cutoffs has not been clearly presented, to guide the user when interpreting the network data and deciding which pseudogene appearing in the different networks is worth looking into more depth or reject as being a false positive result. This probably could be done with their validated use cases example taken for the literature such as PTEN /PTENP1 etc.

Answer: The reviewer brings up an important point and as a result we include a benchmarking analysis for 31 gene-pseudogene groups that are involved in cancer. We extracted the benchmark dataset from PMID: 26442270, PMID: 22726445, and PMID: 29240947. PMID:26442270 is a review of well documented pseudogenes and their functions by a well-known researcher Dr. Poliseno. PMID: 22726445 is a Cell article detailing expressed pseudogenes across 13 human cancers and their targets. PMID: 29240947 is a bench science paper about FTH1 regulation by its pseudogenes. This article also describes some of the other pseudogene-gene relationships described by the previous two papers.

We use this benchmarking experiment in place of Figure 4 because it contains much more information. We derived these associations from well-known studies on the subject and found that we can identify 87% of the groups using all databases, 65% using consensus sequences, and identify 3 benchmark gene-pseudogene pairs using consensus sequences that did not appear using BLAST. The examples found by the consensus sequence method but not by BLAST show that the CUDAlign method is useful. Since best practice would have a researcher try multiple databases, a researcher will identify most of the benchmarks. We believe it is also worth noting that we identified these relationships independently of known relationships. As a result, there will inevitably be subtle differences due to the data and methods used during the generation of different flavors of databases.

Benchmarking table
Gene BLAST CUDAlign18 CUDAlign54 CUDAlign135 CUDAlign198 PMID
PTEN Yes No No No No 26442270
TUSC No No No No No 26442270
INTS6 Yes No No No No 26442270
OCT4 Yes Yes Yes Yes Yes 26442270
HMGA1 Yes Yes Yes Yes Yes 26442270
CYP4Z1 No No No No No 26442270
BRAF Yes No No No No 26442270
KLK4 No No No No No 22726445
ATP8A2 No Yes Yes No No 22726445
CXADR No Yes Yes Yes Yes 22726445
CALM2 Yes Yes Yes Yes Yes 22726445
TOMM40 Yes Yes Yes Yes Yes 22726445
NONO Yes Yes Yes Yes Yes 22726445
PERP No Yes Yes Yes Yes 22726445
DUSP8 Yes Yes No No No 22726445
YES1 Yes Yes No No No 22726445
GJA1 Yes No No No No 22726445
AURKA Yes Yes Yes Yes Yes 22726445
RHOB No No No No No 22726445
HMGB1 Yes Yes Yes Yes Yes 22726445
EIF4A1 Yes Yes No No No 22726445
EIF4H Yes Yes Yes Yes Yes 22726445
SNRP6 Yes Yes Yes Yes Yes 22726445
RAB1 Yes No No No No 22726445
VDAC1 Yes Yes No No No 22726445
RCC2 Yes No No No No 22726445
PTMA Yes Yes Yes Yes Yes 22726445
NDUFA9 Yes Yes Yes Yes Yes 22726445
CES7 Yes No No No No 22726445
EPCAM Yes Yes Yes Yes Yes 22726445
FTH1 Yes Yes Yes Yes Yes 29240947
Hits 24/31 20/31 16/31 15/31 15/31
Total hits 27/31

1) Pg 10 highlighted 9 pseudogenes aligned to 15000 gene families and could highlight potential errors in the annotation or if they are collagen-like pseudogenes or znf-pseudogenes with repetitive features that align everywhere would be interesting to highlight and give a list of the genes in a table.

Answer: We agree with the reviewer on this point and as a result further elaborate upon the high homology pseudogenes, and described these findings in more detail in paragraph 2-3 of Results. Specifically, we found zinc finger pseudogenes and other domain binding patterns in the highest homology pseudogenes. We found large bodies of evidence that the high homology pseudogenes have either direct or indirect relationships with zinc finger genes.
In Results paragraph 2:
“Another feature of note is that there are some pseudogenes that align to many gene families (e.g., 9 pseudogenes, UBE2Q2P1, RP11-313J2.1, TPTEP1, BMS1P1, CTD-2245F17.3, SCAND2P, GTF2IP7, WHAMMP3, IGLV3-2, have alignment scores above 54 in 15,000 gene families and 571 pseudogenes, see Supplementary Table 2, have alignment scores above 54 in 1,000 gene families).”
In Results paragraph 3:
“Of the 9 highest homology pseudogenes (Supplementary Table 2), one, RP11-313J2.1, is a zinc finger

pseudogene and two, CTD-2245F17.3 and SCAND2P, are located in the promoters of zinc finger genes. Four pseudogenes in the 9 highest homology pseudogenes (RP11-313J2.1, CTD-2245F17.3, SCAND2P, and WHAMMP3) also have 92-96% sequence identity with zinc finger genes (ZNF72P, ZNF518A, ZNF37A and ZNF788P/ZNF20 respectively) when BLAST searched against the human genome. Of the 571 highest homology pseudogenes (Supplementary Table 2), we found 27 zinc finger pseudogenes. Using EnrichR59 we identified enrichment in GO Molecular Function GO:0004430 1-phosphatdylinositol 4-kinase activity (Fisher's exact test p-value = 0.001), and enrichment for GO Biological Process GO:0070475 rRNA base methylation (Fisher's exact test p-value = 0.003). In the ARCHS4 database60 324 transcription factors were significantly co-expressed (Benjamini-Hochberg adjusted Fisher's exact test p-value < 0.05) with members of the 571 highest homology pseudogenes. Of those 324 transcription factors, 228 were zinc finger genes. These findings show that the highest homology pseudogenes, like zinc finger genes, likely contain repetitive elements that align to many genomic loci."

2) Fig 3 show the different CUDAlign cutoff and overlap with Pseudogene.org. However there is no detailed explanation why there are over 3500 pseudogenes are not detected by this method of alignment using blast or CDUAlign and is there anything specific about these pseudogenes, are they all 1:1 relationship with parent gene?

Answer: The reviewer identifies an important area, which we have been working on since the initial submission. We have found that a significant portion of these pseudogenes/genes that are in the newer version of the Pseudogenes.org database are not contained in our GENCODEv25 annotation. These missing genes and pseudogenes account for 1030 of the 2458 pseudogene-gene pairs that are in Pseudogenes.org but not in our databases. If these are excluded we recreate 85% of the Pseudogenes.org pseudogene-gene pairs (1:1 relationships). Furthermore this 85% accuracy is similar to our benchmarking accuracy (87%) on genes whose annotation will likely not change drastically between annotation builds. Alternatively, since these genes and pseudogenes were from a different annotation version the sequences themselves could be slightly different causing differences between our database and Pseudogenes.org. These results can be found in the Results section "Direct comparison to pseudogene parents".
In Results paragraph 5:
"Our databases also generate a larger pool of possible interactions. It is worth noting that 391 pseudogenes and 152 genes in the new Pseudogene.org (GENCODEv10) are not present in the GENCODEv25 annotation used in our analysis. These genes and pseudogenes together account for 1030 edges that were used in our comparison. Accounting for these differences in the annotation, we are able to reconstruct 85% of the pseudogene-gene relationships in the new Pseudogene.org database. Since these associations were generated without prior pseudogene-gene relationship information and the annotations have changed slightly since Pseudogenes.org, our methods prove to independently identify known and unknown pseudogene-gene relationships at a high rate."

3) For the use case example, I do not fully understand why the CDUAlign18 was used for PPPARIL identification in sox15 and not detected in the CDUAlign54 or CDUAlign135. Looking at the sox15 network using CDUAlign135 an alternative pseudogene PIN2 pseudogene can be found. Can the authors explain why this is not also considered as potential regulator and why it does not appear in the TGCA expression panel with the rest of the sox genes ?

Answer: We thank the reviewer for their insight and have further evaluated the SOX15 network in response. RP11-506B6.5, the pseudogene located next to PIN2, is retained in the more stringent databases (e.g, CUDAlign135) and as such should also be considered. However, RP11-506B6.5 lacks enough annotation from existing literature to make it a promising candidate. The PPPARIL gene has more supporting literature and is a larger more complex pseudogene containing 19 exons opposed to 1 exon in RP11-506B6.5.

4) Since the usability of the web app is highlighted in the paper, I would recommend a direct link from the Ensembl Identifiers to Ensembl rather than Genecards eg ENST00000428294 does not have a Genecard entry but is classified as a transcribed unprocessed pseudogene by GENCODE/Ensembl.

Answer: We thank the reviewer for this suggestion and as a result have added this functionality to the website. When a user selects a network node, a tab for GeneCards and a tab for Ensembl appears for the specified gene/pseudogene.

5) Also the network in the webapp would be easier to navigate if the HGNC identifier was used as default name rather than the ENSG ID (as this should be relatively easy to code) and therefore recommend

figure 4 be redrawn as looks extremely hard to interpret.

Answer: We appreciate these suggestions and have focused on our main application that is available online (https://integrativeomics.shinyapps.io/pseudofun_app/). In this web application HGNC identifiers are used throughout. The old Figure 4 is from another application we developed for research purposes through the Ohio Supercomputer Center. As a result, we moved the old Figure 4 to the supplementary material so that it does not detract from the usability of the main application (which has much more improved user-friendly visualization) freely available online.

6) Fig 4 should have details of the CDU align cut off used in the legend for the network graphs similar to fig 3

Answer: We thank the reviewers for their concerns and have added this information to Figure 4, which has been moved to the supplementary material as Supplementary Figure 2. We feel that this figure is of less importance after running the benchmarking experiment, shown in Table 2.

Minor issues:
* Pg12 line 12 "regulation" typo

* Pg 16 sentence should have "network" inserted before gene on line

Answer: We appreciate the help from the reviewer for identifying language errors in the manuscript and have made the changes.

Close
