# Supplementary material for: PseudoFuN: Deriving functional potentials of pseudogenes from integrative relationships with genes and microRNAs across 32 cancers
Source: Gigascience. 2019 Apr 26;8(5):giz046. doi: 10.1093/gigascience/giz046 (PMC6486473; doi:10.1093/gigascience/giz046)
Supplement: Response_to_Reviewer_Comments_Revision_1.pdf [file giz046_response_to_reviewer_comments_revision_1.pdf]

Author's Response To Reviewer Comments

Close

Response Letter

Response to editorial issues:
1) Please include a citation to your new GigaDB repository to the bibliography, and cite it from the "data availability" section (the same way as you have done it for the GitHub site).

The citation is:

[xx] Johnson T; Li S; Franz E; Huang Z; Li SD; Campbell MJ; Huang K; Zhang Y Supporting data for "PseudoFuN: Deriving functional potentials of pseudogenes from integrative relationships with genes and miRNAs across 32 cancers" GigaScience Database 2019. https://urldefense.proofpoint.com/v2/url?u=http-3A__dx.doi.org_10.5524_100577&d=DwIGaQ&c=k9MF1d71ITtkuJx-PdWme51dKbmfPEvxwt8SFEkBfs4&r=ekJXXuaVVww1UTqb5FGVAqKm1D8j8S7Zredd9hAkY7k&m=-JC_daUTsqZc2BJqT0Y7Gk63PpDti3noA1ESRxQKWG4&s=SyHXIQfP5-vHlv7kVDFTMlWU8RPnS3jS-u9bzUGKalg&e=

In the data availability section, please write, for example, "Supporting data and an archival copy of the code are availablwe via the GigaScience database, GigaDB [xx]", or similar wording.

Authors' answer: Done.

2) Please include a section "Availability of supporting source code and requirements", formatted as described in our Instructions for authors.

List the following:

Project name: e.g. My bioinformatics project
Project home page: e.g. https://urldefense.proofpoint.com/v2/url?u=https-3A__github.com_ISA-2Dtools&d=DwIGaQ&c=k9MF1d71ITtkuJx-PdWme51dKbmfPEvxwt8SFEkBfs4&r=ekJXXuaVVww1UTqb5FGVAqKm1D8j8S7Zredd9hAkY7k&m=-JC_daUTsqZc2BJqT0Y7Gk63PpDti3noA1ESRxQKWG4&s=NtadpIgDdzem-mFJrMEk2A_FtIP9KrILUbNh9_7DiCc&e=
Operating system(s): e.g. Platform independent
Programming language: e.g. Java
Other requirements: e.g. Java 1.3.1 or higher, Tomcat 4.0 or higher
License: e.g. GNU GPL, FreeBSD etc.
RRID: if applicable, e.g. RRID: SCR_014986

==
Please register any new software application in the https://urldefense.proofpoint.com/v2/url?u=http-3A__SciCrunch.org&d=DwIGaQ&c=k9MF1d71ITtkuJx-PdWme51dKbmfPEvxwt8SFEkBfs4&r=ekJXXuaVVww1UTqb5FGVAqKm1D8j8S7Zredd9hAkY7k&m=-JC_daUTsqZc2BJqT0Y7Gk63PpDti3noA1ESRxQKWG4&s=ctsWxoeEpsMQfIAvLUT27I_ssqxLZZ_7mE5J68Ke8hA&e= database to receive a RRID (Research Resource Identification Initiative ID) number, and include this in your manuscript. This will facilitate tracking, reproducibility and re-use of your tool.

=
Related to software availability, you write "we provide another interactive web application hosted on Ohio Supercomputer Center (OSC) OnDemand, which supports querying novel sequences against any of our PGG databases and visualization of the resulting PGG networks." - please add details in the manuscript how this web application can be accessed.

Authors' answer: All done. We have added a section and made a Research Resource Identification Initiative ID, SCR_017095.

3) Please remove any highlighting in red that was made for the purpose of peer review.

Authors' answer: Done.

4) Please also note the minor comment of the reviewer (below)

Authors' answer: Yes. We have added the missing highlighting color back into Table 2.

5) Please submit your revised version of the text in Word /LaTex (whatever you used to prepare the document) - we only seem to have a PDF version at the moment.

Authors' answer: We will submit Word text with this revision.

PS: We added one affiliation for the corresponding author, and modified the Acknowledgements in the main text.

Response to reviewer reports:
Reviewer #2: I appreciate the effort that the authors put in to addressing my comments and added the additional benchmarking table 2.
One minor comment concerning table 2 is that the highlighting described in the figure could not be seen in the table, otherwise I have no further amendments.

Authors' answer: We appreciate the great suggestions from the reviewers. We have added the missing highlighting color back into Table 2.

Close
