# Supplementary material for: PseudoFuN: Deriving functional potentials of pseudogenes from integrative relationships with genes and microRNAs across 32 cancers
Source: Gigascience. 2019 Apr 26;8(5):giz046. doi: 10.1093/gigascience/giz046 (PMC6486473; doi:10.1093/gigascience/giz046)
Supplement: Reviewer_1_Report_Original_Submission -- Cameron Bracken — 10/21/2018 Reviewed [file giz046_reviewer_1_report_original_submission.pdf]

Reviewer Report

Title: PseudoFuN: Deriving functional potentials of pseudogenes from integrative relationships with genes and miRNAs across 32 cancers

Version: Original Submission **Date:** 10/21/2018

Reviewer name: Cameron Bracken

Reviewer Comments to Author:

The role of pseudogenes in the modulation of gene regulation is a burgeoning field that is ideally placed to benefit from integrative approaches that utilise "big data" that is currently available. A user friendly tool such as PseudoFun is therefore of use as a possible discovery mechanism for new relationships. Not having used PsuedoFun at this stage, it is difficult to fully evaluate its performance, though the approach described appears useful and the presentation of new relationships such as that suggested between PPP4RiL, SOX15 and miR-375 highlight a potential to identify new avenues for further investigation. I have only minor suggestions for improvement in presentation.
1) In Figure 5 (and much of the supplementary figures presented in a similar fashion), is the miRNA associated directly targeting the gene/pseudogene. Visually, only a correlative expression relationship is indicated.
2) Figure 4 does little to add clarity. If the goal is to highlight regulatory relationships, the ENSTxxx labelling does not lend for easy interpretation and the miRNAs are not shown. If the intended purpose is to illustrate a style at which data is outputted, perhaps this is better served by a user friendly series of screenshots illustrating a beginning to end data query - result flow?
3) In Figure 5 and some supplementary figures, co-expression visually is not well represented by the colour scheme. ie: the tumour relationship between PPP4R1L and SOX15. The stats support this, the visual representation less so. Perhaps blanking out the 1:1 same gene : same gene diagonal would allow re-setting of the colour scheme to better represent co-expression?
4) In paragraph 2 of the results, I was unclear what the "alignment score above 54" means... What degree of alignment is this? I found understanding this to be hard to gauge. Relating to this, could the authors comment more extensively on their findings of tremendous levels of alignment for some pseudogenes?

Level of Interest

Please indicate how interesting you found the manuscript: Choose an item.

Quality of Written English

Please indicate the quality of language in the manuscript: Choose an item.

Declaration of Competing Interests

Please complete a declaration of competing interests, considering the following questions:

- Have you in the past five years received reimbursements, fees, funding, or salary from an organisation that may in any way gain or lose financially from the publication of this manuscript, either now or in the future?
- Do you hold any stocks or shares in an organisation that may in any way gain or lose financially from the publication of this manuscript, either now or in the future?
- Do you hold or are you currently applying for any patents relating to the content of the manuscript?
- Have you received reimbursements, fees, funding, or salary from an organization that holds or has applied for patents relating to the content of the manuscript?
- Do you have any other financial competing interests?
- Do you have any non-financial competing interests in relation to this paper?

If you can answer no to all of the above, write 'I declare that I have no competing interests' below. If your reply is yes to any, please give details below.

I have no competing interests

I agree to the open peer review policy of the journal. I understand that my name will be included on my report to the authors and, if the manuscript is accepted for publication, my named report including any attachments I upload will be posted on the website along with the authors' responses. I agree for my report to be made available under an Open Access Creative Commons CC-BY license (http://creativecommons.org/licenses/by/4.0/). I understand that any comments which I do not wish to be included in my named report can be included as confidential comments to the editors, which will not be published.

I agree to the open peer review policy of the journal

To further support our reviewers, we have joined with Publons, where you can gain additional credit to further highlight your hard work (see: https://publons.com/journal/530/gigascience). On publication of this paper, your review will be automatically added to Publons, you can then choose whether or not to claim your Publons credit. I understand this statement.

Yes Choose an item.
