# Supplementary material for: PseudoFuN: Deriving functional potentials of pseudogenes from integrative relationships with genes and microRNAs across 32 cancers
Source: Gigascience. 2019 Apr 26;8(5):giz046. doi: 10.1093/gigascience/giz046 (PMC6486473; doi:10.1093/gigascience/giz046)
Supplement: Reviewer_2_Report_Original_Submission -- Jennifer Harrow — 11/10/2018 Reviewed [file giz046_reviewer_2_report_original_submission.pdf]

Reviewer Report

Title: PseudoFuN: Deriving functional potentials of pseudogenes from integrative relationships with genes and miRNAs across 32 cancers

Version: Original Submission **Date:** 11/10/2018

Reviewer name: Jennifer Harrow

Reviewer Comments to Author:

The authors have presented an overview of their new analysis and data resource to identify novel pseudogene-gene network interactions that could lead to new hypothesis around their role in regulation of cancer using TCGA cancer expression data and miRNA expression. The unique element of this analysis is using a consensus sequence representing gene families and examining the local alignment of pseudogenes against this consensus to identify new potential interactions.

The major criticism of the paper is that a thorough benchmarking evaluation of their different alignment cutoffs has not been clearly presented, to guide the user when interpreting the network data and deciding which pseudogene appearing in the different networks is worth looking into more depth or reject as being a false positive result.T his probably could be done with their validated use cases example taken for the literature such as PTEN /PTENP1 etc.

* Pg 10 highlighted 9 pseudogenes aligned to 15000 gene families and could highlight potential errors in the annotation or if they are collagen-like pseudogenes or znf-pseudogenes with repetitive features that align everywhere would be interesting to highlight and give a list of the genes in a table.
* Fig 3 show the different CUDAlign cutoff and overlap with pseudogene.org. However there is no detailed explanation why there are over 3500 pseudogenes are not detected by this method of alignment using blast or CDUAlign and is there anything specific about these pseudogenes, are they all 1:1 relationship with parent gene?
* For the use case example, I do not fully understand why the CDUAlign18 was used for PPPARIL identification in sox15 and not detected in the CDUAlign54 or CDUAlign135. Looking at the sox15 network using CDUAlign135 an alternative pseudogene PIN2 pseudogene can be found. Can the authors explain why this is not also considered as potential regulator and why it does not appear in the TGCA expression panel with the rest of the sox genes ?
* Since the usability of the web app is highlighted in the paper, I would recommend a direct link from the Ensembl Identifiers to Ensembl rather than Genecards eg ENST00000428294 does not have a Genecard entry but is classified as a transcribed unprocessed pseudogene by GENCODE/Ensembl.
* Also the network in the webapp would be easier to navigate if the HGNC identifier was used as default name rather than the ENSG ID (as this should be relatively easy to code) and therefore recommend figure 4 be redrawn as looks extremely hard to interpret.
* Fig 4 should have details of the CDU align cut off used in the legend for the network graphs similar to fig 3

Minor issues:

* Pg12 line 12 "regulation" typo
* Pg 16 sentence should have "network" inserted before gene on line

Level of Interest

Please indicate how interesting you found the manuscript: Choose an item.

Quality of Written English

Please indicate the quality of language in the manuscript: Choose an item.

Declaration of Competing Interests

Please complete a declaration of competing interests, considering the following questions:

- Have you in the past five years received reimbursements, fees, funding, or salary from an organisation that may in any way gain or lose financially from the publication of this manuscript, either now or in the future?
- Do you hold any stocks or shares in an organisation that may in any way gain or lose financially from the publication of this manuscript, either now or in the future?
- Do you hold or are you currently applying for any patents relating to the content of the manuscript?
- Have you received reimbursements, fees, funding, or salary from an organization that holds or has applied for patents relating to the content of the manuscript?
- Do you have any other financial competing interests?
- Do you have any non-financial competing interests in relation to this paper?

If you can answer no to all of the above, write 'I declare that I have no competing interests' below. If your reply is yes to any, please give details below.

I declare I have no competing interest

I agree to the open peer review policy of the journal. I understand that my name will be included on my report to the authors and, if the manuscript is accepted for publication, my named report including any attachments I upload will be posted on the website along with the authors' responses. I agree for my report to be made available under an Open Access Creative Commons CC-BY license (http://creativecommons.org/licenses/by/4.0/). I understand that any comments which I do not wish to be included in my named report can be included as confidential comments to the editors, which will not be published.

I agree to the open peer review policy of the journal

To further support our reviewers, we have joined with Publons, where you can gain additional credit to further highlight your hard work (see: https://publons.com/journal/530/gigascience). On publication of this paper, your review will be automatically added to Publons, you can then choose whether or not to claim your Publons credit. I understand this statement.

Yes Choose an item.
